# Systematic evaluation of CRISPR-Cas systems reveals design principles for genome editing in human cells

**DOI:** 10.1186/s13059-018-1445-x

**Published:** 2018-05-29

**Authors:** Yuanming Wang, Kaiwen Ivy Liu, Norfala-Aliah Binte Sutrisnoh, Harini Srinivasan, Junyi Zhang, Jia Li, Fan Zhang, Charles Richard John Lalith, Heyun Xing, Raghuvaran Shanmugam, Jia Nee Foo, Hwee Ting Yeo, Kean Hean Ooi, Tore Bleckwehl, Yi Yun Rachel Par, Shi Mun Lee, Nur Nadiah Binte Ismail, Nur Aidah Binti Sanwari, Si Ting Vanessa Lee, Jan Lew, Meng How Tan

**Affiliations:** 1 School of Chemical and Biomedical Engineering, Nanyang Technological University, Singapore, 637459 Singapore; 2 Genome Institute of Singapore, Agency for Science Technology and Research, Singapore, 138672 Singapore; 3 School of Biological Sciences, Nanyang Technological University, Singapore, 637551 Singapore; 4 Lee Kong Chian School of Medicine, Nanyang Technological University, Singapore, 636921 Singapore; 5 School of Applied Science, Republic Polytechnic, Singapore, 738964 Singapore; 6 School of Life Sciences and Chemical Technology, Ngee Ann Polytechnic, Singapore, 599489 Singapore

**Keywords:** CRISPR, Genome editing, Non-homologous end joining (NHEJ), Homology-directed repair (HDR), Cas9 nucleases, Cpf1 nucleases

## Abstract

**Background:**

While CRISPR-Cas systems hold tremendous potential for engineering the human genome, it is unclear how well each system performs against one another in both non-homologous end joining (NHEJ)-mediated and homology-directed repair (HDR)-mediated genome editing.

**Results:**

We systematically compare five different CRISPR-Cas systems in human cells by targeting 90 sites in genes with varying expression levels. For a fair comparison, we select sites that are either perfectly matched or have overlapping seed regions for Cas9 and Cpf1. Besides observing a trade-off between cleavage efficiency and target specificity for these natural endonucleases, we find that the editing activities of the smaller Cas9 enzymes from *Staphylococcus aureus* (SaCas9) and *Neisseria meningitidis* (NmCas9) are less affected by gene expression than the other larger Cas proteins. Notably, the Cpf1 nucleases from *Acidaminococcus* sp. BV3L6 and *Lachnospiraceae* bacterium ND2006 (AsCpf1 and LbCpf1, respectively) are able to perform precise gene targeting efficiently across multiple genomic loci using single-stranded oligodeoxynucleotide (ssODN) donor templates with homology arms as short as 17 nucleotides. Strikingly, the two Cpf1 nucleases exhibit a preference for ssODNs of the non-target strand sequence, while the popular Cas9 enzyme from *Streptococcus pyogenes* (SpCas9) exhibits a preference for ssODNs of the target strand sequence instead. Additionally, we find that the HDR efficiencies of Cpf1 and SpCas9 can be further improved by using asymmetric donors with longer arms 5′ of the desired DNA changes.

**Conclusions:**

Our work delineates design parameters for each CRISPR-Cas system and will serve as a useful reference for future genome engineering studies.

**Electronic supplementary material:**

The online version of this article (10.1186/s13059-018-1445-x) contains supplementary material, which is available to authorized users.

## Background

The CRISPR (clustered regularly interspaced short palindromic repeats)-Cas system is a versatile tool that has been successfully used to modify the genome of myriad organisms [1–10]. In this system, an endonuclease (typically Cas9 or Cpf1) is recruited to a specific genomic locus by a chimeric single guide RNA (sgRNA), which comprises both a crRNA spacer that recognizes the target site by reverse complementary base pairing and a stem loop-containing scaffold for the nuclease [11–13]. Additionally, the target locus must carry a short sequence signature, known as the protospacer adjacent motif (PAM), before DNA cleavage can occur. Hence, the PAM places a constraint on which parts of a genome may be cut by a particular Cas nuclease. Nevertheless, Cas enzymes from different bacteria species generally recognize different PAMs. Therefore, by incorporating various CRISPR-Cas systems into our engineering toolbox, we can expand the range of sites to target in a genome.

Distinct endogenous DNA repair pathways are harnessed to achieve desired genome engineering outcomes [12, 13]. Typically, after the Cas endonuclease cleaves the DNA at the target site, the double-stranded break is repaired by either the non-homologous end joining (NHEJ) pathway or the homology-directed repair (HDR) pathway. The former is activated in the absence of a repair template. Being error-prone, it frequently introduces insertions or deletions (indels) during the repair process, which may result in frameshift mutations and gene knockouts. On the other hand, the latter is activated when a homologous repair template is supplied. Precise DNA changes are specified in the template and are hence incorporated into the target locus with high fidelity by the HDR pathway. However, NHEJ is the dominant repair pathway in higher eukaryotes. Consequently, precise genome editing via homologous recombination (HR) usually occurs at a very low frequency.

Several CRISPR-Cas systems from different bacterial species have been deployed for genome engineering in human cells [1, 2, 4, 14–17]. So far, the vast majority of studies utilize SpCas9 for both NHEJ-mediated and HDR-mediated genome editing largely because it is the first Cas endonuclease to be successfully used in human cells [1, 2, 4] and is also the best characterized enzyme to date. Additionally, the nuclease’s relatively simple NGG PAM requirement contributes to its popularity for genome engineering. However, a major disadvantage of SpCas9 is its relatively large size (1368 amino acids), which hinders certain in vivo therapeutic applications. Some Cas9 homologs, including SaCas9 (1053 amino acids) and NmCas9 (1082 amino acids), help alleviate the size issue, but they typically require more complex PAMs, such as NNGRRT for SaCas9 and NNNNGATT for NmCas9 [18].

Given the widespread occurrence of CRISPR-Cas in bacteria, users are currently uncertain about the relative performance of each system in engineering mammalian genomes. Despite the abundant literature available, it is difficult to directly compare the studies that employ different Cas nucleases due to inconsistencies in cellular context, target sites, protein expression levels, and other experimental conditions. Two recent reports attempted to assess SpCas9 with Cpf1 nucleases, but they were limited in scope and focused mainly on the specificities of Cpf1 [19, 20]. Here, we rigorously characterize three Cas9 nucleases (SpCas9, SaCas9, and NmCas9) and two Cpf1 nucleases (also known as Cas12a) from *Acidaminococcus* sp. BV3L6 and *Lachnospiraceae* bacterium ND2006 (AsCpf1 and LbCpf1, respectively) across a plethora of target sites. Our data provide much-needed guidance to others who are keen to leverage the CRISPR-Cas technology to perform genome editing in human cells and potentially also in other organisms.

## Results

### Framework for fair evaluation of different CRISPR-Cas systems

We sought to establish an evaluation framework that allowed an unbiased assessment of the five Cas endonucleases (SpCas9, SaCas9, NmCas9, AsCpf1, and LbCpf1). Every protein was fused to two nuclear localization signals (NLS) and an identical V5 epitope tag. Additionally, we expressed each enzyme and its cognate sgRNA from the same plasmid backbone. The CAG or the EF1α promoter was used to drive the expression of the Cas nuclease, while the same U6 promoter was used to drive the expression of the sgRNA. After cloning, we verified the activity of each construct using target sites that were known to be edited robustly by the respective nucleases (Additional file 1: Figure S1 and Tables S1 and S2).

The various Cas enzymes should ideally be targeting identical genomic loci in order for the results to be comparable. As each endonuclease requires a different PAM for efficient cleavage and the PAMs for SaCas9 and NmCas9 are incompatible, we initially selected 61 matched target sites that are flanked by TTTN (PAM for AsCpf1 and LbCpf1) and either NGGRRT (combined PAM for SpCas9 and SaCas9) or NGGNGATT (combined PAM for SpCas9 and NmCas9) (Additional file 1: Table S3). The sites ranged in length from 17 to 23 nucleotides (nt). Additionally, since each Cas nuclease may be differentially affected by chromatin accessibility, we targeted genes with varying expression levels in HEK293T cells because gene transcription is largely controlled by the underlying chromatin architecture [21]. Based on our RNA-seq data, the chosen genes showed more than 4000-fold difference in expression (Additional file 1: Figure S2a). Consistently, we also observed higher levels of H3K27ac at the promoters of more actively transcribed genes (Additional file 1: Figure S2b).

We asked whether our evaluation studies may be influenced by the choice of promoter used to express the Cas enzymes. We checked the expression of each endonuclease from either the CAG or the EF1α promoter by quantitative real-time PCR (qRT-PCR) and found that transcript levels were approximately 1.5-fold higher under the latter promoter (Additional file 1: Figure S2c). However, the cleavage efficiencies of Cas nucleases expressed under the CAG promoter were highly correlated with the cleavage efficiencies of the enzymes expressed under the EF1α promoter, regardless of whether T7 endonuclease I (T7E1) mismatch cleavage assays or Illumina deep sequencing experiments were used to measure the rate of indel formation (Pearson R^2^ = 0.75 or 0.96, respectively) (Additional file 1: Figure S2d). The data obtained from the CAG promoter was also not significantly different from the data obtained from the EF1α promoter (*P* > 0.5, Wilcoxon rank sum test; Additional file 1: Figure S2e). This may be because both promoters were strong enough to produce sufficient amounts of Cas proteins, so that enzyme concentration in the cells was no longer a limiting factor. Hence, we pooled the data obtained using the CAG promoter with the data obtained using the EF1α promoter to perform a combined analysis.

### Performance of CRISPR-Cas in NHEJ-mediated genome editing

**Fig. 1:**
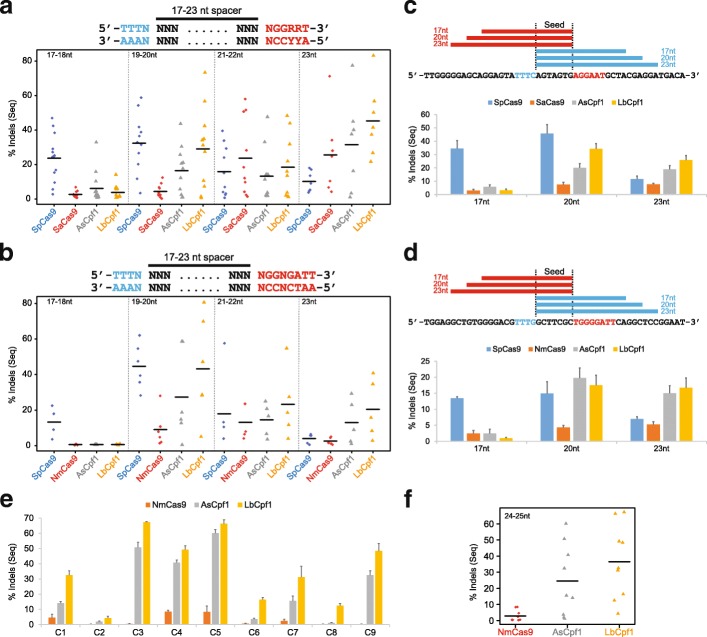
Evaluation of various CRISPR-Cas systems in NHEJ-mediated genome editing using perfectly matched spacers or spacers with overlapping seeds. **a, b** Summary of matched target site activities (see Additional file 1: Figure S4) for SpCas9, either **a** SaCas9 or **b** NmCas9, AsCpf1, and LbCpf1 based on deep sequencing. Each *horizontal bar* indicates the mean of the editing activities for the indicated enzyme and range of spacer lengths. **c, d** Extent of genome modifications at a target locus in the **c** CACNA1D or **d** PPP1R12C gene whereby the Cas9 and Cpf1 nucleases had overlapping seed regions. Three different spacer lengths (17, 20, and 23 nt) were tested. The editing efficiencies were determined by deep sequencing. The cells were harvested 24 h after transfection. Data represent mean ± standard error of the mean (s.e.m.; *n* ≥ 6). **e** The editing activity of NmCas9 and the two Cpf1 nucleases at nine new target sites (C1–C9) of the form TTTN-N_24-25_-NNNNGATT (see Additional file 1: Table S5). The cells were harvested 24 h after transfection and then the editing frequencies were quantified by deep sequencing. Data represent mean ± s.e.m. (*n* ≥ 4). **f** Strip chart summarizing the editing efficiencies of NmCas9, AsCpf1, and LbCpf1 at perfectly matched target sites of longer lengths (24–25 nt)

We first examined the editing activities of the CRISPR-Cas systems without any repair template. Both the T7E1 cleavage assays (Additional file 1: Figure S3) and Illumina deep sequencing experiments (Additional file 1: Figure S4) were used to assess activity at the 61 selected genomic loci in HEK293T cells. Overall, SpCas9 exhibited the highest cleavage efficiencies for spacer lengths between 17 and 20 nt inclusive (Fig. 1a, b and Additional file 1: Figure S5a, b). In particular, it was the only nuclease that was consistently active with short 17-nt spacers, which we further confirmed in other cell lines (Additional file 1: Figure S6). In contrast, SaCas9 and LbCpf1 gave the highest amount of genome modifications for spacer lengths between 21 and 23 nt inclusive (Fig. 1a, b and Additional file 1: Figure S5a, b). Similar results were obtained regardless of whether the matched target sites were present in introns (Additional file 1: Figures S3a–e and S4a–e) or in protein-coding regions (Additional file 1: Figures S3f–h and S4f–h). We also noted that the PAM-proximal seed region of the DNA target is more critical for proper recruitment of the CRISPR-Cas system, but the PAMs for Cpf1 and Cas9 are on opposite sides of each protospacer. Hence, we selected new target sites where the Cpf1 and Cas9 nucleases had overlapping seed regions (PAM-proximal 7 nt; Fig. 1c, d and Additional file 1: Figure S5c, d and Table S4). However, we still observed similar trends with these new sites.

Notably, NmCas9 performed poorly at most of the target sites irrespective of spacer lengths, with editing frequencies considerably lower than the other nucleases (Fig. 1b and Additional file 1: Figure S5b). We also observed that with our chimeric sgRNAs, NmCas9 did not show a preference for longer spacer lengths, consistent with a recent study on the usage of NmCas9 in mammalian genome editing [22]. Nevertheless, since the length of naturally occurring crRNA spacers in *N. meningitides* was found to be 24 nt [23], we selected nine new 24 nt- or 25 nt-long target sites that are flanked by the PAMs for Cpf1 and NmCas9 (Additional file 1: Figure S7a, b and Table S5). Moreover, these sites are in highly expressed genes to ensure accessibility of the chromatin. When we quantified editing efficiencies at these new genomic loci by T7E1 cleavage assays (Additional file 1: Figure S7c, d) and Illumina deep sequencing experiments (Fig. 1e, f) in HEK293T cells, we again found that the editing activity of NmCas9 was lower than those of both AsCpf1 and LbCpf1 at all nine matched target sites. We further verified the poorer performance of NmCas9 in other cell lines (Additional file 1: Figure S8). Collectively, our results suggest that NmCas9 might not be an ideal Cas nuclease for many genome editing applications, such as multiplex gene targeting.

**Fig. 2:**
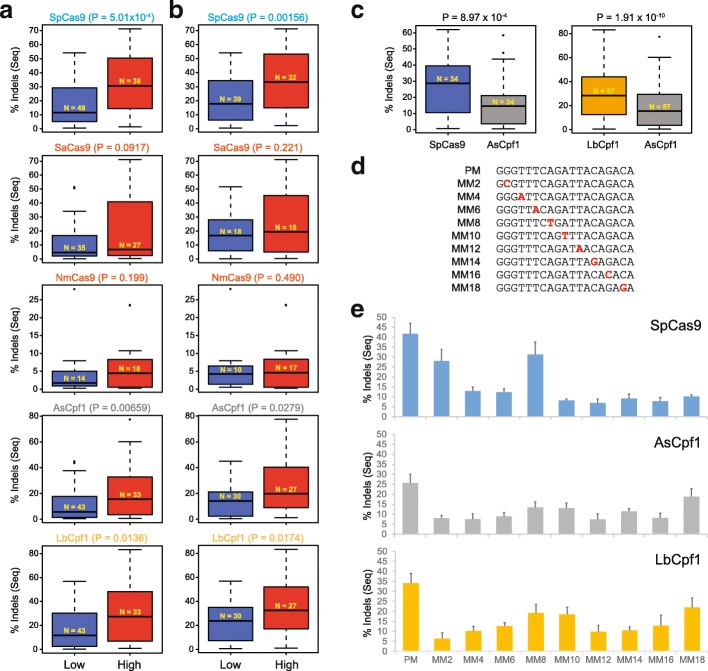
Relationship of DNA cleavage efficiency with gene expression and target specificity. **a** Impact of gene expression on editing efficiency. We divided the target sites into those that occur in lowly expressed genes (FPKM < 25, *blue boxplots*) and those that occur in highly expressed genes (FPKM ≥ 25, *red bloxplots*) using our RNA-seq data. The FPKM value of 25 was chosen to divide the target sites into two groups of roughly equal sizes for the five Cas nucleases. Here, all sgRNAs were considered in the analysis. Overall, we found from our deep sequencing experiments that SpCas9, AsCpf1, and LbCpf1 were able to edit highly expressed genes more efficiently than lowly expressed genes (*P* < 0.05, Wilcoxon rank sum test). In contrast, the two smaller nucleases, SaCas9 and NmCas9, were less affected by gene expression. **b** Similar analysis to **a**, except that only sgRNAs of optimal lengths were considered. In the current study, we set the optimal lengths of SpCas9 as 17–22 nt inclusive, SaCas9 as ≥ 21 nt, NmCas9 as ≥ 19 nt (based on our results in Fig. 1b and Additional file 1: Figure S5b as well as a previous report [22]), AsCpf1 as ≥ 19 nt, and LbCpf1 as ≥ 19 nt. **c** Comparison of AsCpf1 with either SpCas9 (*left boxplot*) or LbCpf1 (*right boxplot*). Only sgRNAs of the optimal lengths for SpCas9 and the Cpf1 nucleases (19–22 nt inclusive) were considered. From deep sequencing analysis, we found that the editing activity of AsCpf1 was significantly lower than that of both SpCas9 and LbCpf1 (*P* < 0.001, Wilcoxon rank sum test). **d** To assess the specificities of SpCas9, AsCpf1, and LbCpf1, we examined the tolerance of these enzymes to single mismatches along the spacer targeting the A17 site in the NF1 gene. *Red letters* indicate the mutated bases. **e** Using the spacers indicated in **d**, we determined the editing activities of SpCas9, AsCpf1, and LbCpf1 by deep sequencing. The cells were harvested 24 h after transfection. For all three nucleases, we observed an increased tolerance to mismatches around the middle of the spacer. Importantly, while SpCas9 and LbCpf1 exhibited higher cleavage efficiencies than AsCpf1 with a perfect matched (PM) spacer, they also showed an overall higher tolerance to mismatches between the spacer and the target DNA. Data represent mean ± standard error of the mean (*n* ≥ 4)

Next, we asked whether the editing efficiency of each Cas endonuclease may be affected by local chromatin context. To increase the statistical power of our analysis, we selected 18 additional target sites that contain NGGRRT at their 3′ ends and are of length 21 nt, which is within the optimal spacer lengths for both SpCas9 and SaCas9 (Additional file 1: Table S6). Six of these sites are in lowly expressed genes, while the remaining 12 sites are in highly expressed genes (Additional file 1: Figure S9). We assayed the activity of each enzyme by the T7E1 assay and by deep sequencing the targeted loci (Additional file 1: Figure S10). When we considered all the selected sites together, we found that the editing efficiencies of SpCas9, AsCpf1, and LbCpf1 were significantly affected by the expression of the targeted genes (*P* < 0.05, Wilcoxon rank sum test; Fig. 2a and Additional file 1: Figure S11a), consistent with previous studies that showed that chromatin structure may influence the efficacy of CRISPR-mediated genome editing [24–27]. The same results were obtained when we restricted our analysis to only the sgRNAs of optimal lengths for every enzyme (Fig. 2b and Additional file 1: Figure S11b). Interestingly, however, the efficacy of SaCas9 and NmCas9 in human cells appeared to be unaffected by gene expression levels, especially when we considered only the sgRNAs of optimal lengths (Fig. 2b and Additional file 1: Figure S11b), possibly because they are smaller enzymes and hence may be able to access nucleosome-bound DNA or heterochromatin more easily.

While AsCpf1 performed generally well in our NHEJ-mediated genome editing experiments, it was usually surpassed by some other enzyme at most target sites, regardless of whether they are located in lowly expressed or highly expressed genes. When we carried out a four-way comparison of the different Cas nucleases using spacers that were either perfectly matched or contained matched seed regions, we found that AsCpf1 was the best performing enzyme at only a minority of the sites, even for optimal spacer lengths (Additional file 1: Figure S12). When we carried out a pairwise comparison of AsCpf1 with either SpCas9 or LbCpf1 alone, focusing only on the sgRNAs of optimal lengths for both enzymes under consideration, we also found that AsCpf1 exhibited significantly lower cleavage efficiencies than the other two nucleases (*P* < 0.05, Wilcoxon rank sum test; Fig. 2c and Additional file 1: Figure S11c). Nevertheless, despite its overall weaker editing activity, AsCpf1 showed the lowest tolerance to single mismatches between the sgRNA and the target DNA (Fig. 2d, e and Additional file 1: Figure S11d, e). Hence, our results suggest that there is a compromise between cleavage efficiency and specificity of naturally occurring Cas endonucleases.

### Performance of CRISPR-Cas in HDR-mediated genome editing with single-stranded oligodeoxynucleotide donor

**Fig. 3:**
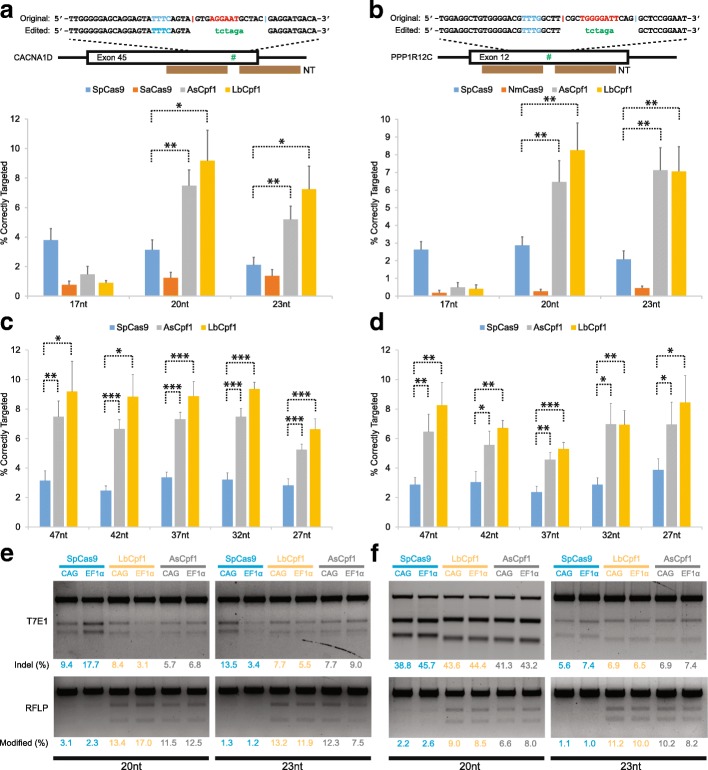
Evaluation of various CRISPR-Cas systems in HDR-mediated genome editing using symmetric ssODN donor templates and spacers with overlapping seeds. **a, b** Extent of XbaI restriction site (depicted in *green*) insertion into a coding exon of the **a** CACNA1D or **b** PPP1R12C gene. The *brown horizontal bars* represent the 47-nt homology arms of the donor template and *NT* indicates that the donor is of the non-target strand sequence. Three different spacer lengths (17, 20, and 23 nt) were tested. The cells were harvested 72 h after transfection and the gene-targeting efficiencies were determined by Illumina deep sequencing. Data represent mean ± standard error of the mean (s.e.m.; *n* ≥ 4). *P < 0.05, **P < 0.01; Student’s *t*-test. **c, d** Extent of precise gene editing by SpCas9, AsCpf1, and LbCpf1 when ssODNs of different homology arm lengths (27–47 nt) were used together with 20-nt spacers targeting **c** CACNA1D or **d** PPP1R12C. The cells were harvested 72 h after transfection and the gene targeting efficiencies were determined by Illumina deep sequencing. Data represent mean ± s.e.m. (*n* ≥ 4). *P < 0.05, **P < 0.01, ***P < 0.001; Student’s *t*-test. **e, f** Concurrent T7E1 assays and RFLP analysis of cells co-transfected with the indicated CRISPR plasmids and donor ssODNs containing 47-nt long homology arms. We used 20 or 23 nt long spacers targeting either **e** CACNA1D or **f** PPP1R12C. The cells were harvested 72 h after transfection. Overall, the cleavage efficiencies of SpCas9 were comparable to those of AsCpf1 and LbCpf1, as determined by the T7E1 assays. However, the extent of XbaI integration into the target sites was lower for SpCas9 compared to AsCpf1 and LbCpf1, as determined by RFLP analysis

We sought to determine how well the various CRISPR-Cas systems perform in HDR-mediated precise genome editing. We again targeted the two genomic loci containing matched seeds for Cas9 and Cpf1 nucleases, but here we co-transfected donor single-stranded oligodeoxynucleotide (ssODN) with our CRISPR plasmids in order to introduce a XbaI restriction site between the cleavage sites of Cas9 and Cpf1 (Fig. 3a, b and Additional file 1: Figure S13a, b). Every ssODN contained the restriction site flanked by 47 nt of homology on each side. The donor templates were also complementary to the target strands. Expectedly, restriction fragment length polymorphism (RFLP) analysis revealed that only SpCas9 was able to consistently insert the XbaI site when the spacer length was just 17 nt. However, for spacers that were 20 or 23 nt long, both AsCpf1 and LbCpf1 gave significantly more digested products than SpCas9 (*P* < 0.05, Student’s *t*-test; Additional file 1: Figure S13a, b). SaCas9 and NmCas9 yielded almost no detectable shorter fragments after restriction digest regardless of spacer lengths, possibly because they cleaved less efficiently than the other Cas nucleases at the two targeted loci (Fig. 1c, d and Additional file 1: Figure S5c, d). We further confirmed the results by deep sequencing to ensure that the restriction site was correctly inserted (Fig. 3a, b). When we reduced the homology arm length of the donor template from 47 to 27 nt, the editing efficiency of each enzyme was unaffected and the Cpf1 nucleases continued to exhibit significantly higher HDR frequencies than SpCas9 (*P* < 0.05, Student’s *t*-test; Fig. 3c, d and Additional file 1: Figure S13c, d). Comparable results were obtained when we varied the amount of donor templates between 100 and 300 ng (Additional file 1: Figure S14). Additionally, we observed that the HDR efficiencies of all Cas nucleases increased with time after transfection (Additional file 1: Figure S15). Moreover, although we detected a small amount of incorrect XbaI integration from our sequencing data, it was, on average, 6.4-fold and 12.4-fold lower than the rate of correct integration at the CACNA1D and PPP1R12C loci, respectively (Additional file 1: Figure S16).

**Fig. 4:**
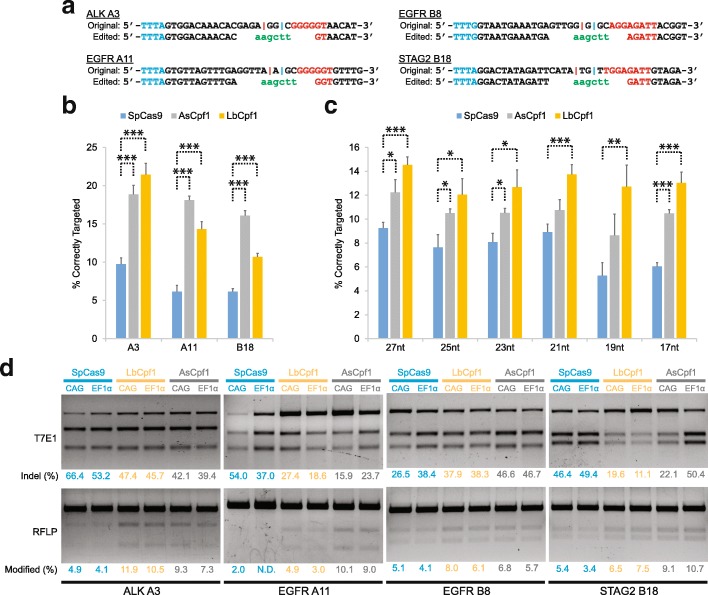
Evaluation of various CRISPR-Cas systems in HDR-mediated genome editing using symmetric ssODN donor templates and perfectly matched spacers. **a** Intended DNA changes at the A3 (in ALK), A11 (in EGFR), B8 (in EGFR), and B18 (in STAG2) target sites. Each *red vertical line* indicates the cleavage site of Cas9 nucleases, which occurs 3 bp upstream of their PAM. Each *blue vertical line* indicates the cleavage site of Cpf1 nucleases on one DNA strand, which occurs 18 nt downstream of their PAM. The HindIII restriction site is indicated in *green*. **b** Extent of correctly incorporating the HindIII recognition sequence into the A3, A11, or B18 target locus. Donor ssODNs with 27-nt homology arm lengths were used. The donor templates were complementary to the target DNA strand. Cells were harvested for deep sequencing analysis 72 h post-transfection. Both the Cpf1 endonucleases consistently exhibited higher levels of precise gene targeting than SpCas9. Data represent mean ± standard error of the mean (s.e.m.; *n* ≥ 5). ***P < 0.001; Student’s *t*-test. **c** Extent of precise gene editing by SpCas9, AsCpf1, and LbCpf1 at the B8 locus when ssODNs of different homology arm lengths (17–27 nt inclusive) were used. The donor templates were complementary to the target DNA strand. Cells were harvested 72 h after transfection and the gene targeting efficiencies were determined by Illumina deep sequencing. Data represent mean ± s.e.m. (*n* ≥ 3). *P < 0.05, **P < 0.01, ***P < 0.001; Student’s *t*-test. **d** Concurrent T7E1 assays and RFLP analysis of cells co-transfected with the indicated CRISPR plasmids and donor ssODNs containing 27 nt long homology arms for the A3, A11, B8, and B18 target sites. Overall, the cleavage efficiencies of SpCas9 were comparable to those of AsCpf1 and LbCpf1, as determined by the T7E1 assays. However, the extent of HindIII integration into the target sites was lower for SpCas9 compared to AsCpf1 and LbCpf1, as determined by RFLP analysis

Subsequently, we selected six perfectly matched target sites, namely A3, A11, A12, B4, B8, and B18, that could be cleaved robustly by at least SpCas9, AsCpf1, and LbCpf1 (Additional file 1: Figures S3 and S4) to perform additional HDR-mediated genome editing experiments with ssODNs as donor templates. For A12 and B4, the ssODNs contained 47-nt homology arms flanking either a XbaI or HindIII recognition sequence, while for the remaining target sites, the ssODNs contained 27-nt homology arms instead. Moreover, for the B8 target site, we also tested extra donor templates with even shorter homology arms (27, 25, 23, 21, 19, and 17 nt). All donor templates were of the non-target strand sequence. Overall, we observed that the Cpf1 nucleases exhibited significantly higher HDR efficiencies at all the six target sites than SpCas9 in RFLP assays and deep sequencing experiments (*P* < 0.05, Student’s *t*-test; Fig. 4a–c and Additional file 1: Figures S17 and S18). The frequency of erroneous restriction site integrations was much lower than the rate of correct integrations (Additional file 1: Figure S19). Since the six additional sites are located in genes of varying expression levels, the higher HDR efficiency exhibited by Cpf1 appears to be independent of the underlying chromatin architecture. Importantly, the editing efficiency of each Cas endonuclease at the B8 locus was not compromised even when we reduced the homology arm length down to 17 nt. This result is consistent with a previous study that found that zinc finger nucleases could perform precise gene editing with templates containing only around 30–40 total bases of homology [28].

We wondered whether the results from our HDR-mediated editing experiments might be due to differences in cleavage efficiencies. After co-transfecting ssODNs with our CRISPR plasmids, we performed T7E1 assays and RFLP analysis on the same genomic DNA samples. Overall, we observed that SpCas9 generated indels as efficiently as AsCpf1 and LbCpf1 in the T7E1 assays, but yet it produced weaker cleavage bands than the Cpf1 nucleases after restriction digest with XbaI or HindIII (Figs. 3e, f and 4d). Additionally, we sequenced the targeted genomic loci and examined the sequencing reads. Strikingly, SpCas9 produced random indels at least as efficiently as AsCpf1 and LbCpf1 at all the tested loci (Additional file 1: Figure S20), but clearly fewer sequencing reads had the desired restriction site correctly incorporated (Additional file 1: Figure S21). Hence, the lower efficiency of precise genome editing exhibited by SpCas9 compared to the Cpf1 nucleases when ssODNs of non-target strand sequences were used was not simply due to a poorer ability to cut the target sites.

### Optimization of ssODN donor templates

The design of the ssODN donor template can influence HDR efficiency [29–32]. So far, all our experiments had relied on symmetric ssODNs of the non-target strand sequence. Hence, we first sought to explore the extent to which the editing activity of each CRISPR-Cas system may be influenced by the orientation of the donor template. To this end, we targeted the CACNA1D and PPP1R12C loci as well as the A3, A11, B8, and B18 loci using ssODNs that were complementary to either the target or the non-target strand. All the ssODNs contained 27-nt homology arms. We also tested ssODNs with 17-nt arms for the B8 locus. Surprisingly, we did not detect a consistent strand bias for each Cas nuclease by deep sequencing experiments (Additional file 1: Figure S22) or by RFLP analysis (Additional file 1: Figure S23). Instead, at five out of the six targeted sites, we observed a trend for the editing activity of all the enzymes to change in the same direction when we altered the orientation of the donor template, thereby suggesting that each genomic locus may have an inherent ssODN strand preference. For example, at the PPP1R12C locus, the HDR frequencies of all the enzymes showed an increase when we switched from the original ssODN template that was of the non-target strand sequence (NT) to a new donor that was of the target strand sequence (T), although this increase was much larger for SpCas9 (Additional file 1: Figures S22b and S23b). Conversely, at the A11 locus, the HDR frequencies of SpCas9, AsCpf1, and LbCpf1 all decreased when we used T ssODNs in place of the original NT ssODNs, although this reduction was more significant for the Cpf1 nucleases (Additional file 1: Figures S22d and S23d). Furthermore, the changes in HDR frequencies were not simply due to differences in cleavage rates as every nuclease yielded similar amounts of indels in the presence of either the NT or the T ssODNs (Additional file 1: Figure S24).

**Fig. 5:**
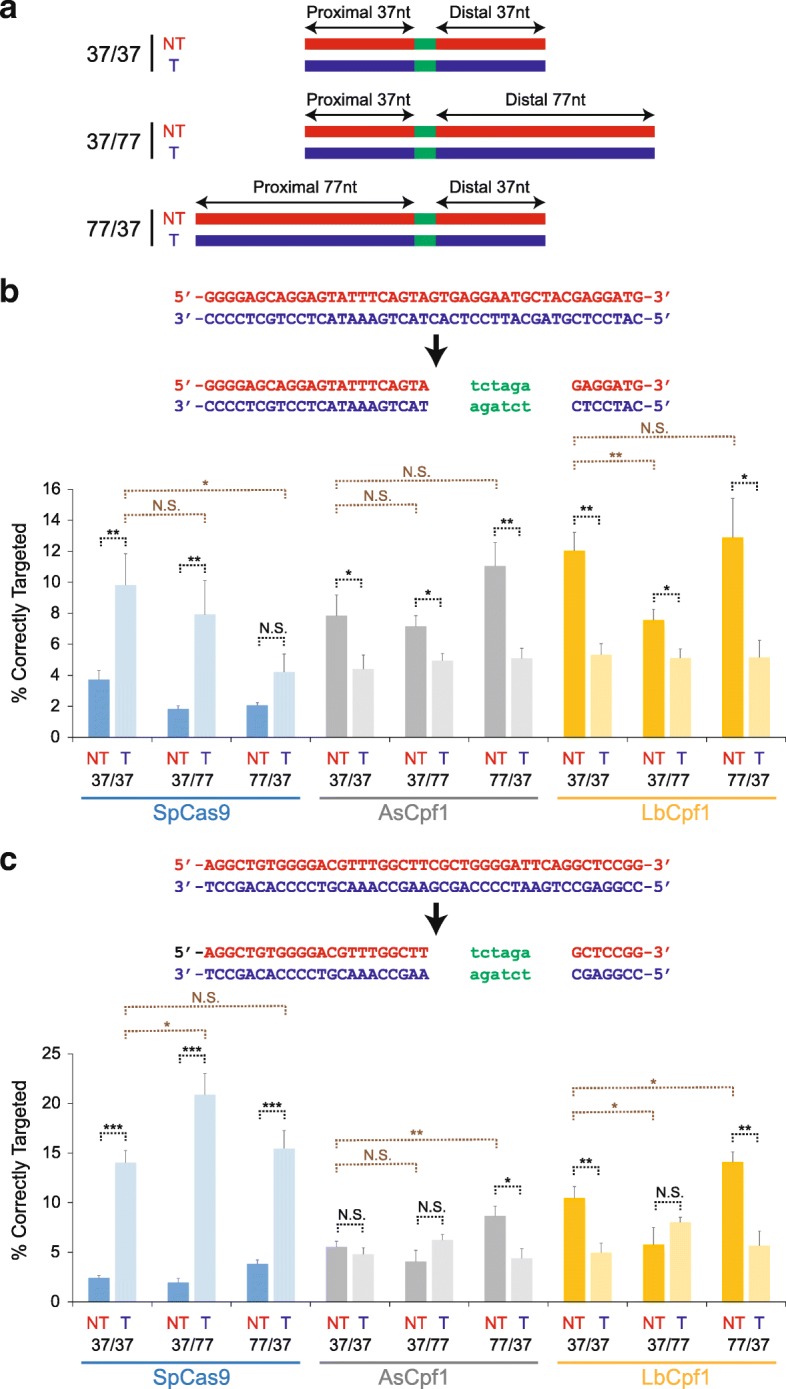
Evaluation of multiple symmetric and asymmetric ssODN donor designs used in combination with different CRISPR-Cas systems. **a** Various types of ssODN donor templates tested. Each single-strand DNA donor is complementary to either the target strand (and hence is of the non-target strand sequence and is denoted “*NT*”) or the non-target strand (and hence is of the target strand sequence and is denoted “*T*”). The NT ssODN donor is colored *red*, while the T ssODN donor is colored *blue*. The *green box* between the homology arms indicates the restriction site to be integrated into the target genomic locus. The 37/77 T ssODN has previously been found to be optimal for SpCas9-induced HDR [31]. **b, c** Extent of precise gene editing by SpCas9, AsCpf1, and LbCpf1 at the **b** CACNA1D or **c** PPP1R12C locus. Cells were harvested 72 h after transfection and the gene targeting efficiencies were determined by Illumina deep sequencing. Data represent mean ± standard error of the mean (s.e.m.; *n* ≥ 4). *P < 0.05, **P < 0.01, ***P < 0.001, *N.S.* not significant; Student’s *t*-test

An earlier study showed that the strand bias of SpCas9 at an AAVS1 genomic locus became more obvious with longer donor templates [30]. Hence, to better detect any such bias, we next used ssODNs with 37-nt homology arm lengths to edit the CACNA1D and PPP1R12C loci. In agreement with previous work [29–31], we found from deep sequencing experiments (Fig. 5) and RFLP analysis (Additional file 1: Figure S25) that SpCas9 exhibited significantly higher HDR efficiencies at both genomic loci when donor DNA complementary to the non-target strand was used (*P* < 0.05, Student’s *t*-test). In contrast, we also now observed that the NT ssODNs were consistently more effective than the T ssODNs at introducing precise edits at both loci for the Cpf1 nucleases. Hence, Cas9 and Cpf1 prefer donor templates of opposite orientations.

Subsequently, we sought to determine whether the structure of the ssODN could further impact on the editing efficiency of the Cas enzymes. A previous study demonstrated that homology-directed editing by SpCas9 could be enhanced by using asymmetric donor templates [31]. Here, to create such asymmetric donors, we extended either the PAM-proximal or the PAM-distal side of each ssODN from 37 to 77 nt (Fig. 5a). Again, we tested donor DNA that was complementary to either the target or the non-target strand of the CACNA1D or PPP1R12C locus. Consistent with the published report [31], we found that for SpCas9, extending the homology arm at the PAM-distal side of the T ssODN could improve HDR efficiency, while extending the homology arm at the PAM-proximal side was either neutral or detrimental to the performance of the enzyme (Fig. 5b, c and Additional file 1: Figure S25). In contrast, we discovered that for the Cpf1 nucleases, extending the homology arm at the PAM-proximal side of the NT ssODN instead led to an increase in HDR frequency, while extending the homology arm at the PAM-distal side decreased the rate of HDR. Overall, LbCpf1 still exhibited a higher HDR efficiency than SpCas9 at the CACNA1D locus when all possible types of donor DNA had been considered, but at the PPP1R12C locus, the HDR rate exhibited by SpCas9 with its optimal ssODN template was significantly higher than that exhibited by LbCpf1 with its optimal donor template (*P* < 0.05, Student’s *t*-test). Taken together, our results indicate that both SpCas9 and LbCpf1 may be used for ssODN-mediated editing, but strand preferences of the genomic locus and the enzyme as well as the structure of the donor template need to be carefully considered.

### Enhancement of error-prone repair with long single-stranded DNA

**Fig. 6:**
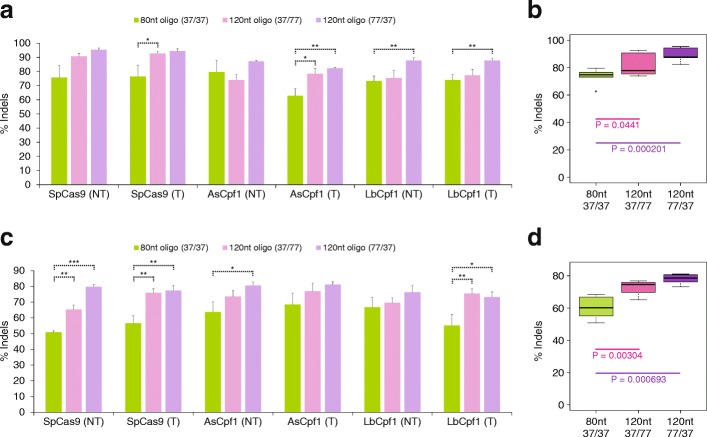
Gene disruption efficiencies in the presence of different single-stranded DNA donors. **a, c** Extent of indel formation at the **a** CACNA1D or **c** PPP1R12C genomic locus when various ssODN donor templates were used in combination with SpCas9, AsCpf1, or LbCpf1. The rates were quantified by Illumina deep sequencing. *NT* and *T* indicate donors of non-target and target strand sequences, respectively. Data represent mean ± standard error of the mean (*n* ≥ 4). *P < 0.05, **P < 0.01, ***P < 0.001; Student’s *t*-test. **b, d** Boxplot summarizing the rates of indel formation at the **b** CACNA1D or **d** PPP1R12C locus for oligonucleotides of different lengths and structures. There was no significant difference in the indel frequencies between NT and T ssODN donors and hence their data were pooled

Our deep sequencing data afforded us an opportunity to examine the cleavage efficiencies of the Cas enzymes in the presence of various types of donor DNA. Overall, the presence of symmetric ssODNs with homology arm lengths ranging from 17 to 47 nt (corresponding to single-stranded DNA of lengths 40 to 100 nt) did not affect the frequency of indel formation significantly (Additional file 1: Figures S20, S24, S26). However, we observed that the rate of such error-prone repair outcomes tended to increase when we used the longer asymmetric ssODNs, whose total length was 120 nt. This increase was observed at both the CACNA1D (Fig. 6a, b) and the PPP1R12C (Fig. 6c, d) loci for all the Cas nucleases and appeared to be more significant for ssODNs with a longer PAM-proximal homology arm (Fig. 6b, d). Additionally, the higher indel frequencies were unlikely to account for the increased HDR rates achieved with optimized ssODN donor templates (Fig. 5 and Additional file 1: Figure S25) because suboptimal asymmetric donors that caused a decrease in HDR rates could also boost the frequencies of indel formation. We further noted from a previous study that even non-homologous 127-mer single-stranded DNA could stimulate gene disruption by SpCas9 [33]. Collectively, our results suggest a strategy whereby the efficiency of gene knockout may be enhanced by introducing a long ssODN donor that contains a frameshift or a nonsense mutation flanked by asymmetric homology arms, so that the target gene could be inactivated not only by a stimulated error-prone repair pathway but also by the HDR pathway using an optimized single-stranded DNA donor.

### Performance of CRISPR-Cas in HDR-mediated genome editing with plasmid donor

**Fig. 7:**
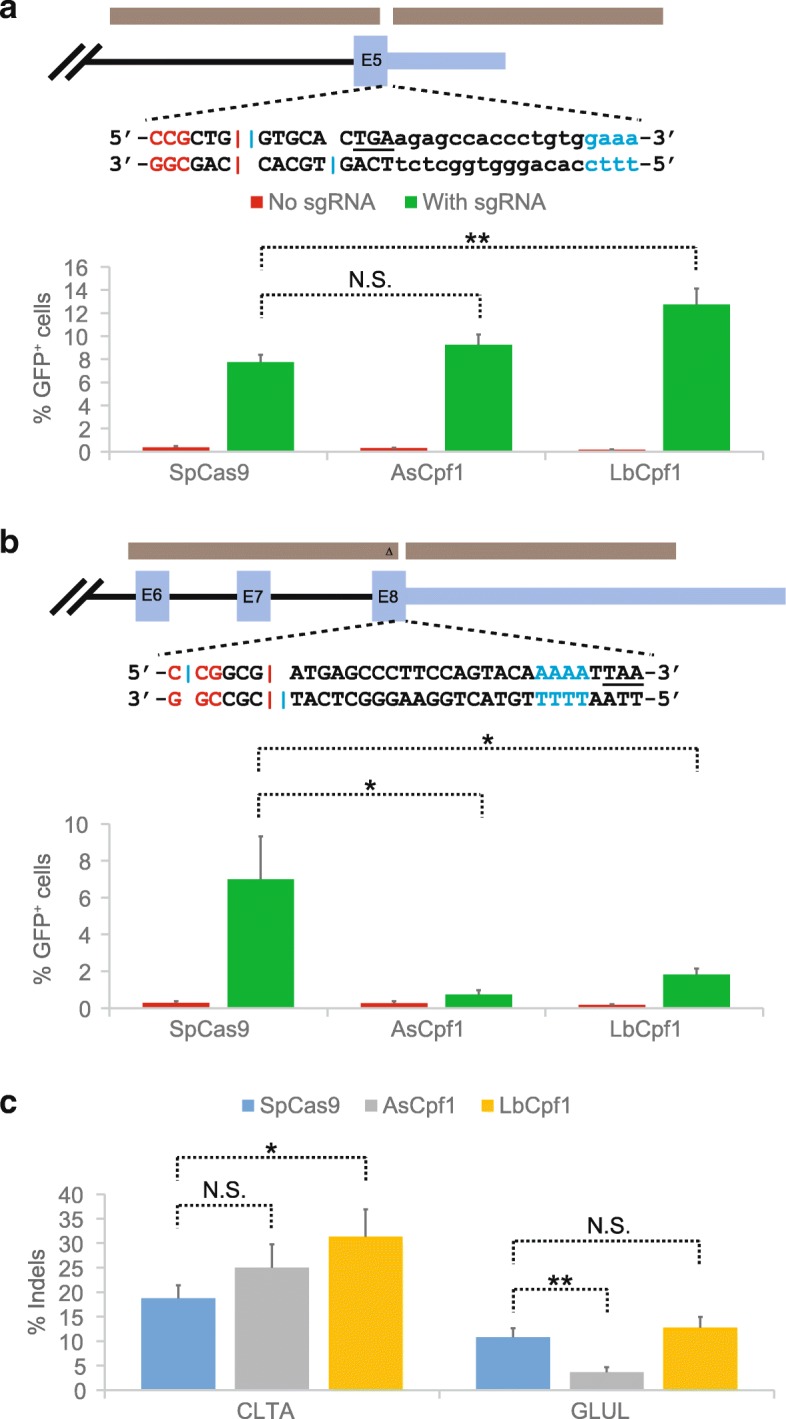
Evaluation of various CRISPR-Cas systems in HDR-mediated genome editing using linearized plasmid donor templates. **a, b** Fusing eGFP to the C-terminus of **a** CLTA and **b** GLUL via a P2A self-cleaving peptide. In the schematics of the targeted loci, the *blue boxes* depict the exons (E), the *brown horizontal bars* indicate the homology arms (1000–1500 nt each), and the *small triangle* in the left homology arm of the GLUL donor indicates that there are several third base pair (bp) mutations towards the end of the coding region to prevent re-targeting by the Cas nucleases. Within the nucleotide sequences, the *red vertical lines* indicate the Cas9 cleavage sites, while the *blue vertical lines* indicate the Cpf1 cleavage sites. Expectedly, the percentages of GFP-positive cells were very low when no sgRNA was transfected (*red bars*), but showed a clear increase in the presence of the appropriate sgRNA for all three Cas nucleases (*green bars*). Data represent mean ± standard error of the mean (s.e.m.; *n* ≥ 5). *P < 0.05, **P < 0.01, *N.S.* not significant; Student’s *t*-test. **c** Rate of indel formation as determined by T7E1 assays. The cleavage efficiencies observed could largely explain the rates of eGFP knockin shown in **a, b**. Data represent mean ± s.e.m. (*n* ≥ 8). *P < 0.05, **P < 0.01, *N.S.* not significant; Student’s *t*-test

Finally, we asked how well SpCas9 would perform against AsCpf1 and LbCpf1 in HDR-mediated genome editing with a linearized plasmid donor, which is commonly used to integrate an epitope tag into an endogenous target locus. Here, we aimed to fuse enhanced green fluorescent protein (eGFP) to the C-terminus of CLTA and GLUL, which were selected because the SpCas9 and Cpf1 nucleases could theoretically cleave both genes at overlapping sites close to the translation stop codon (Fig. 7a, b). FACS analysis revealed that Cpf1 did not give a higher rate of eGFP integration than SpCas9 when differences in cleavage efficiencies (Fig. 7c) were taken into account. For CLTA, the relative HDR efficiency of the three Cas endonucleases paralleled the relative cleavage efficiency observed in T7E1 assays. For GLUL, SpCas9 exhibited a significantly higher knockin rate than both AsCpf1 and LbCpf1 because AsCpf1 cleaved significantly more poorly than SpCas9 at this target site (*P* < 0.05, Student’s *t*-test) and also possibly because the blunt cut created by SpCas9 is overall nearer to the stop codon than the staggered cut created by Cpf1 and CRISPR-facilitated gene tagging is known to be more efficient closer to the break site. Similar results were obtained when we varied the amount of donor plasmids between 300 and 900 ng (Additional file 1: Figure S27). We further confirmed by PCR the correct integration of eGFP into the CLTA and GLUL genomic loci regardless of the Cas nuclease used (Additional file 1: Figure S28). Collectively, our results indicate that SpCas9 performs favorably compared to the Cpf1 enzymes in precision genome engineering when linearized plasmids are used as donor templates.

## Discussion

**Fig. 8:**
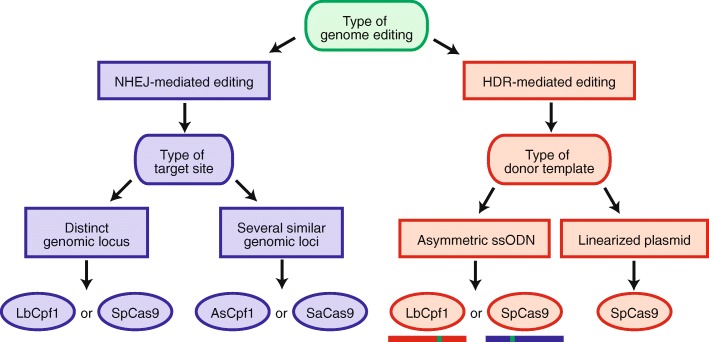
Flowchart to guide CRISPR-Cas users in selecting the appropriate system for their experiments. The choice of Cas endonuclease depends on several considerations, such as the type of genome modification desired or the type of repair template to be utilized. For HDR-mediated editing with ssODNs, we recommend asymmetric donors that are complementary to either the target or non-target strand when used in combination with LbCpf1 or SpCas9, respectively

CRISPR-Cas is a powerful technology for engineering the complex genomes of plants and animals, but users are frequently befuddled by which particular system to adopt. Direct comparisons of published studies performed by different laboratories are unreliable due to numerous confounding factors, including differences in cellular contexts and target sites. Consequently, most users tend to rely on SpCas9 as a default. Here, we conducted a survey of five distinct CRISPR-Cas systems across numerous target loci to obtain a comprehensive view of how each Cas endonuclease performs against one another in both NHEJ- and HDR-mediated genome editing. From our extensive evaluation study, we derived a set of guidelines to help users of the CRISPR-Cas technology select the most appropriate system for their applications (Fig. 8). Comfortingly, SpCas9 did exhibit the highest cleavage efficiency at more target sites than the other nucleases when the spacer length was constrained to 17–20 nt (Additional file 1: Figure S12). Furthermore, when we compared SpCas9 with LbCpf1 using only the sgRNAs of optimal lengths for the two enzymes, we found that SpCas9 and LbCpf1 generated indels at comparable frequencies in both lowly expressed and highly expressed genes (Additional file 1: Figure S29). Hence, for the purpose of generating routine gene knockouts via the NHEJ pathway, we would recommend utilizing either SpCas9 or LbCpf1. However, there may be situations where target specificity is an issue. For example, one may want to target a repetitive genomic region or a gene with several close paralogs. In such cases, we would recommend AsCpf1 due to its lower tolerance for mismatches between the sgRNA and the target DNA (Fig. 2d, e and Additional file 1: Figure S11d, e). Alternatively, SaCas9 may be used as it requires longer spacers (at least 21 nt) for activity.

The ideal CRISPR-Cas system is one with both high cleavage efficiency and high target specificity. Two recent studies reported that AsCpf1 and LbCpf1 appeared to satisfy both these criteria [19, 20], suggesting that they may be the model Cas endonucleases to pursue in future applications. However, our analysis indicates that there may be a compromise between editing activity and target specificity in naturally occurring Cas enzymes (Fig. 2c–e and Additional file 1: Figure S11c–e). Specifically, both SpCas9 and LbCpf1 showed more robust editing activity than AsCpf1, but they also exhibited higher tolerance for mismatches between their sgRNA and the target DNA. It may be possible that the genome-wide methods used in the two recent studies [19, 20] have some limitations that preclude comprehensive detection of all off-target sites cleaved by the Cpf1 nucleases. Digenome-seq [34, 35] requires very high sequencing depth to capture cleavage sites, while GUIDE-seq [36] requires the incorporation of blunt double-stranded oligodeoxynucleotides (dsODNs) into DNA breakage sites, which may inherently be biased against the staggered cuts generated by Cpf1. Indeed, the efficiency of tag integration for AsCpf1 and LbCpf1 was found to be lower than that for SpCas9 [19]. Hence, additional work is needed to fully investigate the relationship between editing activity and target specificity of all promising natural CRISPR-Cas systems. Newer and more sensitive methods of detecting off-target effects, such as CIRCLE-seq [37] and SITE-Seq [38], may help to resolve the issue. We further note that one may also evolve natural Cas enzymes into variants that achieve both high editing efficiency and targeting specificity, as demonstrated recently for SpCas9 [39].

HDR-mediated precise genome editing typically occurs at low frequencies. This roadblock needs to be overcome before CRISPR-Cas can realize its full potential in gene therapy, whereby accurate correction of disease-causing mutations can lead to a permanent cure. As a result, there have been numerous efforts over the past few years to improve its efficiency [29, 31, 40–46]. Our data indicate that AsCpf1 and LbCpf1 are able to introduce precise genome edits in human cells efficiently when ssODNs are used as donor templates (Figs. 3, 4 and 5 and Additional file 1: Figures S13, S17, S18, S22, S23, S25). We found that the Cpf1 nucleases prefer single-stranded DNA donors that are complementary to the target strand, in contrast to SpCas9, which prefers donor templates that are complementary to the non-target strand instead. In addition, we observed that asymmetric donors with a longer PAM-proximal homology arm could further improve Cpf1-mediated editing. From these results, we propose that Cpf1 may asymmetrically release the 3′ end of the cleaved target strand, thereby allowing the shorter arm of the optimized donor template to anneal and consequently enabling the longer arm of the template to invade and displace the base-paired non-target DNA strand at the other side of the break. We further note that our data, which were obtained in human cells, are in agreement with another recent study performed in zebrafish embryos [32].

It is tempting to speculate that the Cpf1 nucleases should perform precise genome editing more efficiently than SpCas9. First, Cpf1 generates a staggered cut that may facilitate HDR, while Cas9 generates a blunt cut. Second, Cpf1 cleaves outside the critical seed region and hence repeated targeting may occur, while Cas9 cleaves within the seed region and hence re-targeting is less likely to happen because indel mutations will prevent any subsequent recognition by the enzyme [14]. Indeed, our results showed that even when we used ssODNs of the optimal strand sequence, both AsCpf1 and LbCpf1 still yielded higher HDR frequencies than SpCas9 at five out of the six target sites tested (Additional file 1: Figures S22 and S23). However, we note that there might be other confounding factors. For example, our data indicate the presence of an inherent strand bias at each target genomic locus, possibly due to native chromatin context. This localized bias can play in favor of SpCas9’s preference for a ssODN template that is complementary to the non-target strand, which is what we observed at the PPP1R12C locus. Furthermore, we found that SpCas9 and Cpf1 performed comparably when linearized plasmids were used as donor templates (Fig. 7), suggesting that it is not the nature of the cut per se that affects HR efficiency. While this may be explained by the fact that ssODN-mediated editing and plasmid-mediated editing are resolved through different DNA repair pathways, further work is needed to carefully dissect the underlying mechanisms.

## Conclusions

We systematically assessed the ability of different CRISPR-Cas systems to perform NHEJ- and HDR-mediated genome editing in human cells. We targeted numerous genomic loci with matched spacers or matched seeds to obtain a clearer and fairer picture of how the various Cas enzymes compared against one another. Our extensive survey enabled us to formulate a set of rules and design parameters that others may follow to carry out their genome editing experiments with CRISPR-Cas (Fig. 8). We anticipate that the guidelines will evolve with time as the technology matures and more Cas nucleases are discovered and characterized in the future.

## Methods

### Cell culture and transfection

All cell lines were cultured in Dulbecco’s modified Eagle medium (DMEM) supplemented with 10% FBS, 2 mM L-glutamine, and 1% penicillin/streptomycin. Cells were incubated at 37 °C in a humidified 5% CO_2_ air incubator. The cell lines were routinely checked by PCR for mycoplasma contamination using the following primers: forward, GGG AGC AAA CAG GAT TAG ATA CCC T; reverse, TGC ACC ATC TGT CAC TCT GTT AAC CTC.

Transfections were performed using either Turbofect (Thermo Scientific), jetPRIME (Polyplus), or Lipofectamine 2000 (Life Technologies), according to the manufacturers’ instructions. We seeded 350,000 or 120,000 cells in each well of a 12-well plate or a 24-well plate, respectively, one day prior to transfection, so that the cells would be approximately 60% confluent the next day. For NHEJ-mediated editing experiments, HEK293T cells were transfected with 500 ng CRISPR plasmids in 12-well plates and sorted 24 h post-transfection for fluorescent cells. For HDR-mediated editing experiments with ssODNs, HEK293FT cells were co-transfected with 300 ng CRISPR plasmids and 300 ng ssODNs, which were purchased from Integrated DNA Technologies, in 24-well plates and sorted 3 days post-transfection for fluorescent cells. Sequences of the donor ssODNs are provided in Additional file 1: Appendix S1. For HDR-mediated editing experiments with donor plasmids, HEK293FT cells were co-transfected with 300 ng CRISPR plasmids and 300 ng donor templates, which were linearized with either KpnI or SalI, in 24-well plates and analyzed by flow cytometry for GFP-positive cells 9 days post-transfection.

### T7E1 assay and RFLP analysis

Genomic DNA was isolated using QuickExtract (Epicentre) according to the manufacturer’s instructions. The loci-of-interest were then amplified using Q5 High-Fidelity DNA Polymerase (New England Biolabs) and the following PCR parameters: 98 °C for 30s, 98 °C for 10s, 63–65 °C for 30s, 72 °C for 20s (repeat from step 2 for 34 more cycles), and 72 °C for 2 min. Sequences of the primers used are provided in Additional file 1: Table S7. Subsequently, the PCR products were purified using the GeneJET Gel Extraction Kit (Thermo Scientific).

For the T7E1 assay, 200 ng PCR products was incubated at 95 °C for 5 min in 1× NEBuffer 2 and then slowly cooled at a rate of − 0.1 °C/second. After annealing, 5 U T7 endonuclease I (New England Biolabs) was added to each sample and the reactions were incubated at 37 °C for 50 min. The T7E1-digested products were separated on a 2.5% agarose gel stained with GelRed (Biotium) and the gel bands were quantified using ImageJ. For the RFLP analysis, 200 ng PCR products were digested overnight with either XbaI or HindIII-HF (New England Biolabs) in CutSmart buffer. The reactions were separated on a 2% agarose gel stained with GelRed (Biotium) and the gel bands were quantified using ImageJ.

### FACS analysis

Flow cytometry was performed on FACSAriaIII (Becton Dickinson). FSC and SSC were used to separate singlets from cell aggregates. Subsequently, RFP- and GFP-positive cells were gated relative to untransfected control cells. Data analysis was performed using FACSDiva (Becton Dickinson) and FlowJo.

### Illumina deep sequencing analysis

Sequencing libraries were constructed as previously described [47]. Sequences of the PCR primers used to amplify the loci-of-interest are provided in Additional file 1: Table S8. To process the data, we first built a local reference library comprising the amplicon sequences of the targeted genes. The sequenced reads were then mapped against this reference library with BWA-MEM (mismatch penalty = 2 and clipping penalty = 8). The uniquely mapped reads with mapping quality ≥ 20 were sorted and assigned group information using Picard. GATK toolkit was used to perform local realignment and recalibration. The ‘CIGAR’ string of the BAM file was used to classify the reads as ‘Insertion’, ‘Deletion’, and ‘Match’. We used only the portions of the reads within 40 bp upstream and downstream of the spacer for the calculation of indel percentages. Randomly selected reads were also aligned with the relevant reference sequences using the Needleman-Wunsch algorithm. These alignments were then manually compared with our script outputs to ensure the accuracy of our analysis.

For the characterization of HDR-mediated genome editing, we built another local reference library consisting of the modified genes. The procedures described above were repeated, but the mapping was done to the newly built reference library (consisting of the modified genes). The reads were then classified into three categories for calculating the HDR incorporation percentages: ‘Correct incorporation’, when the read is tagged as a match and the restriction site is present; ‘Wrong incorporation’, when the read is tagged as an insertion or deletion and the restriction site is present; ‘No incorporation’, when the restriction site is not present.

### Quantitative real-time PCR

First, RNA was isolated using Direct-zol RNA Miniprep kit (Zymo Research) according to manufacturer’s instructions. cDNA was then synthesized using qScript cDNA Supermix (QuantaBio). Finally, PCR was performed using Perfecta SYBR Fastmix (QuantaBio) according to manufacturer’s instructions and the following primers: OFP set 1 forward, TGA GCA AAA ACG TGA GCG TG; OFP set 1 reverse, ACC ATA CTG AAA TGC CGT GGT; OFP set 2 forward, AAA CGG GGT TCT TGT TGG CT; OFP set 2 reverse, TCA GTC TGC TCA ACC GTC TT.

### Statistical analysis

Statistical tests, including Student’s *t*-test, Wilcoxon rank sum test, and Kolmogorov-Smirnov test, were performed as described in the figure captions. All *P* values were calculated with either the R software package or Microsoft Excel.

## Additional file

Additional file 1: Supplementary figures, tables, and appendix. (PDF 5236 kb)
