# Supplementary material for: Systematic evaluation of CRISPR-Cas systems reveals design principles for genome editing in human cells
Source: Genome Biol. 2018 May 29;19:62. doi: 10.1186/s13059-018-1445-x (PMC5972437; doi:10.1186/s13059-018-1445-x)
Supplement: Supplementary file 1 — Supplementary figures, tables, and appendix. (PDF 5236 kb) [file 13059_2018_1445_MOESM1_ESM.pdf]

SUPPLEMENTARY INFORMATION

Systematic evaluation of CRISPR-Cas systems reveals design principles for genome editing in human cells

Yuanming Wang[1,2,7], Kaiwen Ivy Liu[2,7], Norfala-Aliah Binte Sutrisnoh[2,3], Harini Srinivasan[1,2], Junyi Zhang[1,2], Jia Li[1,2], Fan Zhang[2], Charles Richard John Lalith[2], Heyun Xing[1], Raghuvaran Shanmugam[1,2], Jia Nee Foo[2,4], Hwee Ting Yeo[2,3], Kean Hean Ooi[1,3], Tore Bleckwehl[1], Yi Yun Rachel Par[2,5], Shi Mun Lee[1,2], Nur Nadiah Binte Ismail[2,6], Nur Aidah Binti Sanwari[2,6], Si Ting Vanessa Lee[2,6], Jan Lew[2,6], Meng How Tan[1,2,*]

[1]School of Chemical and Biomedical Engineering, Nanyang Technological University, Singapore 637459, Singapore

[2]Genome Institute of Singapore, Agency for Science Technology and Research, Singapore 138672, Singapore

[3]School of Biological Sciences, Nanyang Technological University, Singapore 637551, Singapore

[4]Lee Kong Chian School of Medicine, Nanyang Technological University, Singapore 636921, Singapore

[5]School of Applied Science, Republic Polytechnic, Singapore 738964, Singapore

[6]School of Life Sciences and Chemical Technology, Ngee Ann Polytechnic, Singapore 599489, Singapore

[7]These authors contributed equally to this work.

[*]Correspondence: mh.tan@ntu.edu.sg or tanmh@gis.a-star.edu.sg

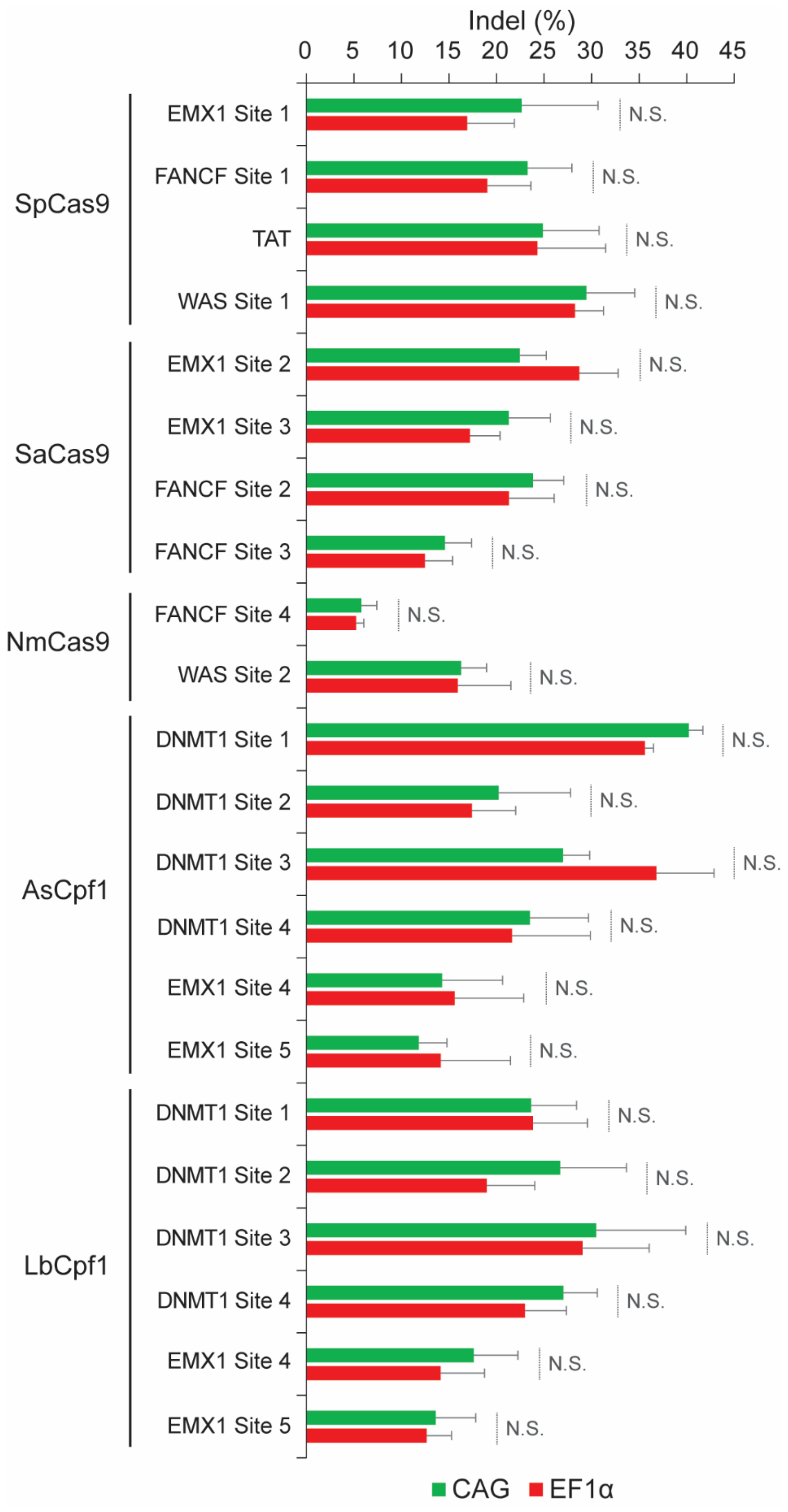

Indel (%)
0
5
10
15
20
25
30
35
40
45
SpCas9
EMX1 Site 1
FANCF Site 1
TAT
WAS Site 1
SaCas9
EMX1 Site 2
EMX1 Site 3
FANCF Site 2
FANCF Site 3
NmCas9
FANCF Site 4
WAS Site 2
AsCpf1
DNMT1 Site 1
DNMT1 Site 2
DNMT1 Site 3
DNMT1 Site 4
EMX1 Site 4
EMX1 Site 5
LbCpf1
DNMT1 Site 1
DNMT1 Site 2
DNMT1 Site 3
DNMT1 Site 4
EMX1 Site 4
EMX1 Site 5
N.S.
CAG
EF1α

Figure S1 Confirmation of cleavage activities of our CRISPR constructs.

We cloned each Cas endonuclease and its cognate sgRNA into the same plasmid backbone and then tested the new constructs for cleavage activities. As determined by T7E1 assays, we observed robust editing activities for all the five nucleases, namely SpCas9, SaCas9, NmCas9, AsCpf1, and LbCpf1. The targeted sites and the primers used for the assays are given in Tables S1, S2 respectively. Data represent mean ± s.e.m ($n \geq 3$ per construct). There was no significant difference in indel rates between CAG-expressed enzymes and EF1α-expressed enzymes at all the genomic loci tested. (N.S.: not significant; Student's t-test)

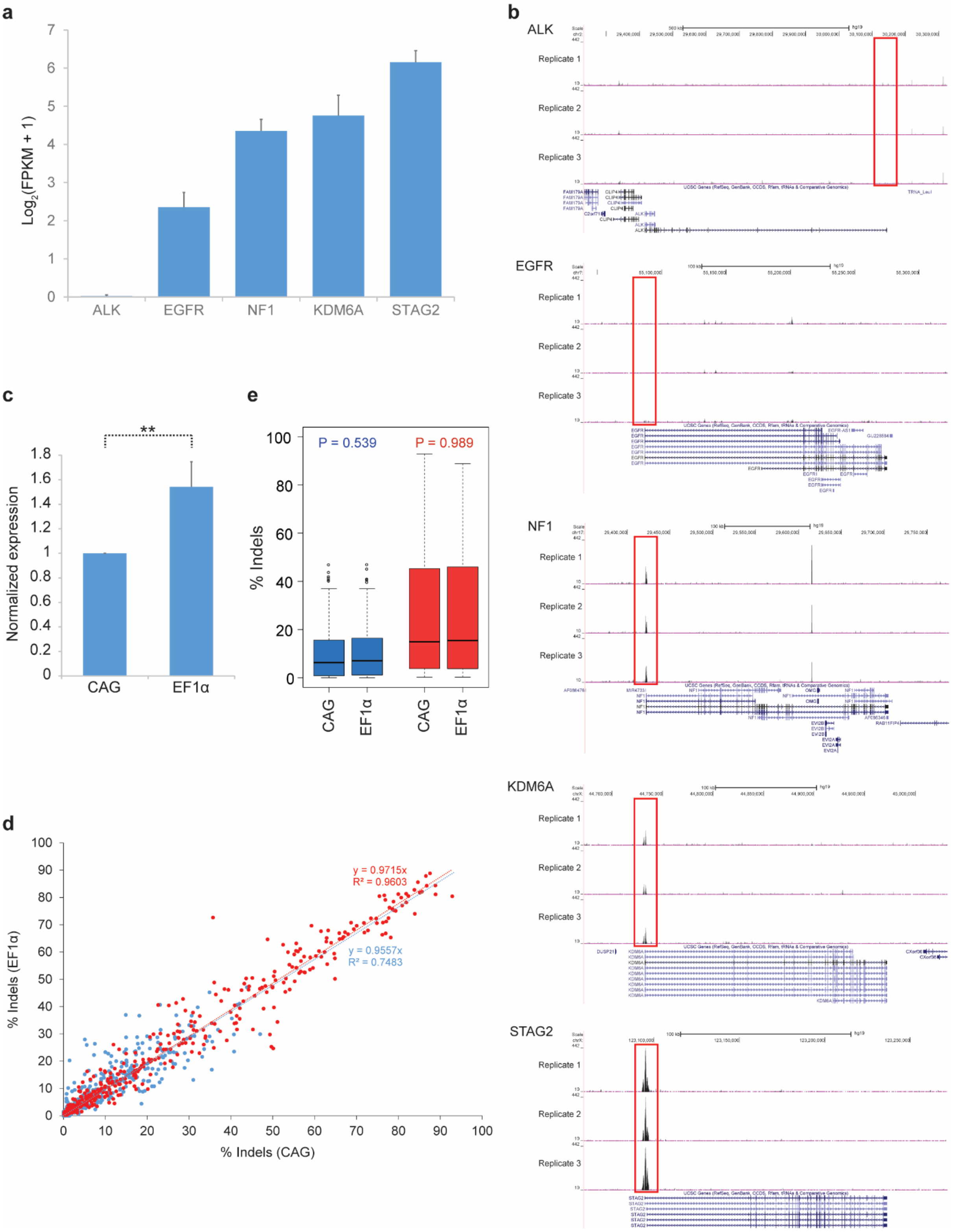
a
Log2(FPKM + 1)
0
1
2
3
4
5
6
7
ALK
EGFR
NF1
KDM6A
STAG2
b
ALK
EGFR
NF1
KDM6A
STAG2
Replicate 1
Replicate 2
Replicate 3
c
**
Normalized expression
0
0.2
0.4
0.6
0.8
1
1.2
1.4
1.6
1.8
CAG
EF1α
e
P = 0.539
P = 0.989
% Indels
0
20
40
60
80
100
CAG
EF1α
CAG
EF1α
d
y = 0.9715x
R² = 0.9603
y = 0.9557x
R² = 0.7483
% Indels (EF1α)
% Indels (CAG)
0
10
20
30
40
50
60
70
80
90
100

Figure S2 Selection of target genes and cleavage efficiencies at chosen sites in the HEK293T cell line.

a Expression levels of five selected genes (ALK, EGFR, NF1, KDM6A, and STAG2) based on RNA-seq experiments. Data represent mean ± s.e.m (n = 6).

b H3K27ac ChIP-seq data for the five selected genes. Red boxes indicate the start of each gene. We observed larger ChIP-seq peaks for genes that were more highly expressed.

c Transcript levels of Cas enzymes under the control of two different promoters. Data represent mean ± s.e.m (n = 4 per enzyme). (** $P < 0.01$, Student's t-test)

d Scatterplot of cleavage efficiencies at all target sites. Blue dots represent data obtained from T7E1 assays, while red dots represent data obtained from Illumina deep sequencing experiments.

e Boxplot summarizing the rates of indel formation quantified by T7E1 assays (blue) or deep sequencing experiments (red). P-values were calculated using the Wilcoxon rank sum test.

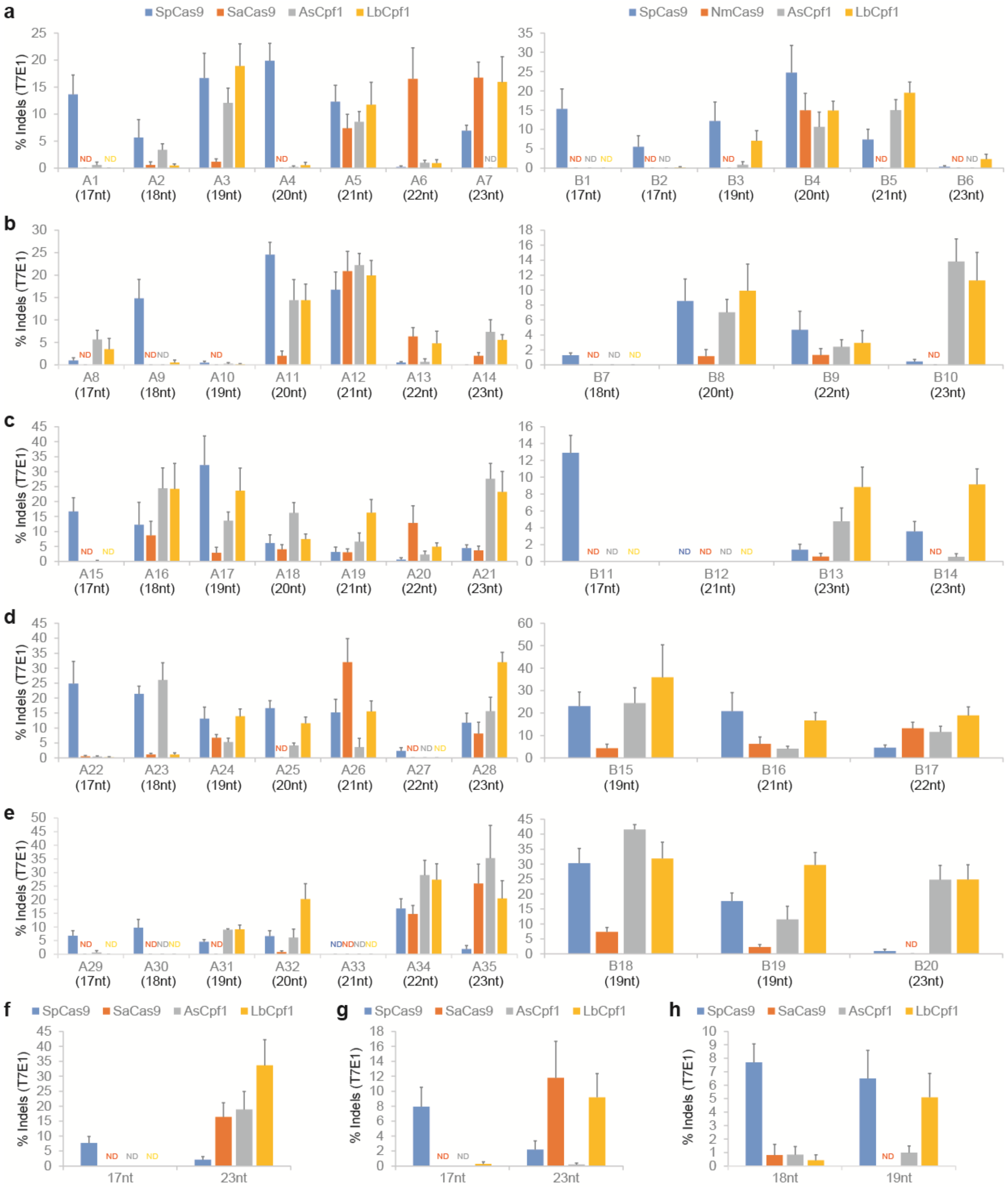
a
SpCas9 SaCas9 AsCpf1 LbCpf1
SpCas9 NmCas9 AsCpf1 LbCpf1
% Indels (T7E1)
ND
A1 (17nt) A2 (18nt) A3 (19nt) A4 (20nt) A5 (21nt) A6 (22nt) A7 (23nt)
B1 (17nt) B2 (17nt) B3 (19nt) B4 (20nt) B5 (21nt) B6 (23nt)
b
A8 (17nt) A9 (18nt) A10 (19nt) A11 (20nt) A12 (21nt) A13 (22nt) A14 (23nt)
B7 (18nt) B8 (20nt) B9 (22nt) B10 (23nt)
c
A15 (17nt) A16 (18nt) A17 (19nt) A18 (20nt) A19 (21nt) A20 (22nt) A21 (23nt)
B11 (17nt) B12 (21nt) B13 (23nt) B14 (23nt)
d
A22 (17nt) A23 (18nt) A24 (19nt) A25 (20nt) A26 (21nt) A27 (22nt) A28 (23nt)
B15 (19nt) B16 (21nt) B17 (22nt)
e
A29 (17nt) A30 (18nt) A31 (19nt) A32 (20nt) A33 (21nt) A34 (22nt) A35 (23nt)
B18 (19nt) B19 (19nt) B20 (23nt)
f
g
h
17nt 23nt
18nt 19nt

Figure S3 Extent of genome modifications as determined by T7E1 assays.

To ensure a fair comparison, matched target sites flanked by optimal PAMs for different Cas endonucleases were selected. Spacer lengths from 17nt to 23nt inclusive were tested. The cells were harvested 24 hours after transfection. Data represent mean ± s.e.m ($n \geq 6$ per target site). (N.D.: not detected)

a – e Intronic sites were selected in genes with varying expression levels. The genes were **a** ALK, **b** EGFR, **c** NF1, **d** KDM6A, and **e** STAG2. We noted that SaCas9 and NmCas9 required incompatible PAMs and hence had to be evaluated separately. The left panels (group A) contained genomic loci targeted by SpCas9, SaCas9, AsCpf1, and LbCpf1. The right panels (group B) contained genomic loci targeted by SpCas9, NmCas9, AsCpf1, and LbCpf1.

f – h Additional target sites in protein-coding regions were selected in **f** APC, **g** ATM, and **h** KDM5C (left panel, 18nt) and ALK (right panel, 19nt).

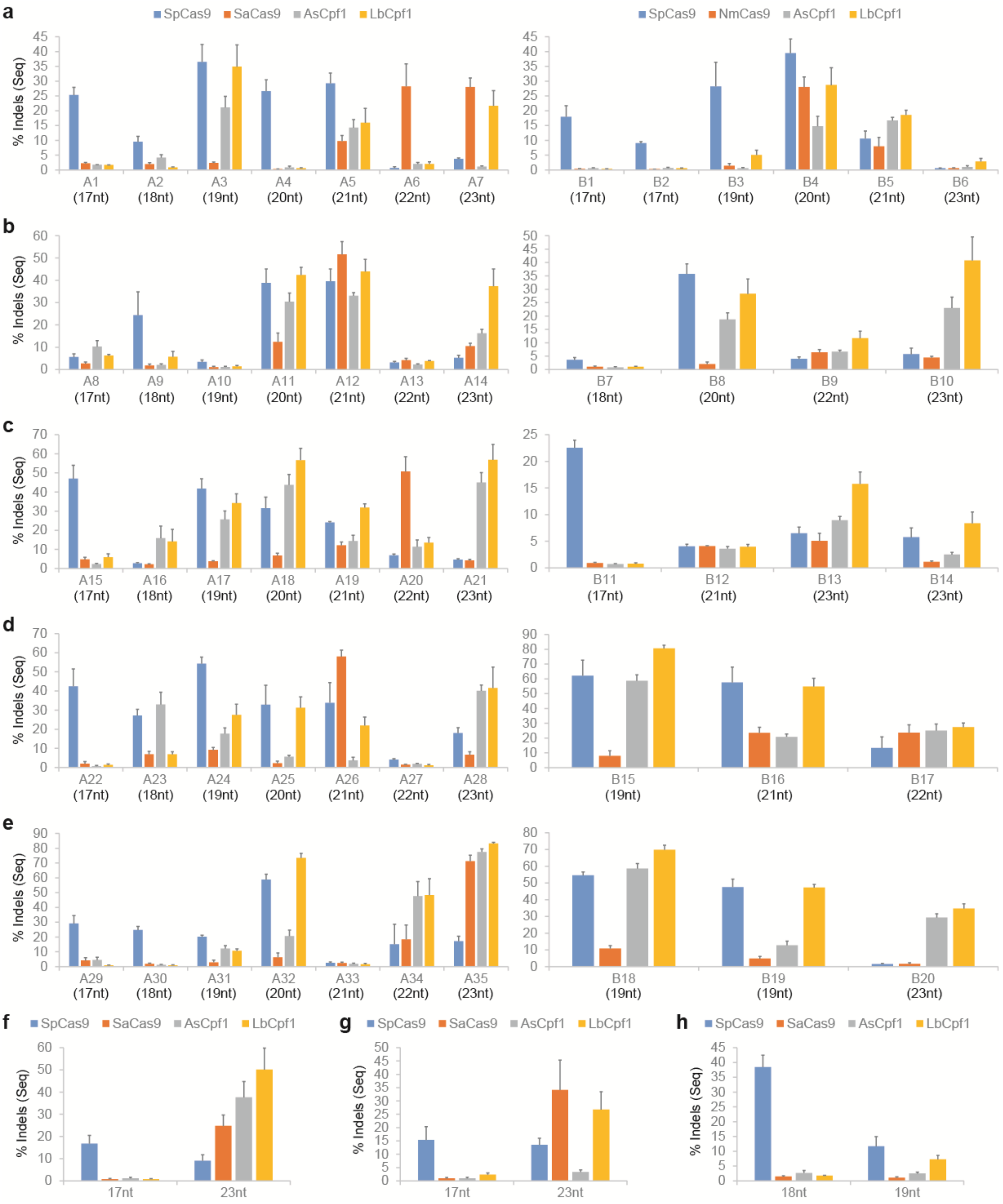

Figure S4 Extent of genome modifications as determined by Illumina deep sequencing. The same genome loci as those in Figure S3 were targeted, with the rate of indel formation measured by deep sequencing instead. The cells were harvested 24 hours after transfection. Data represent mean ± s.e.m (n ≥ 2 per target site).

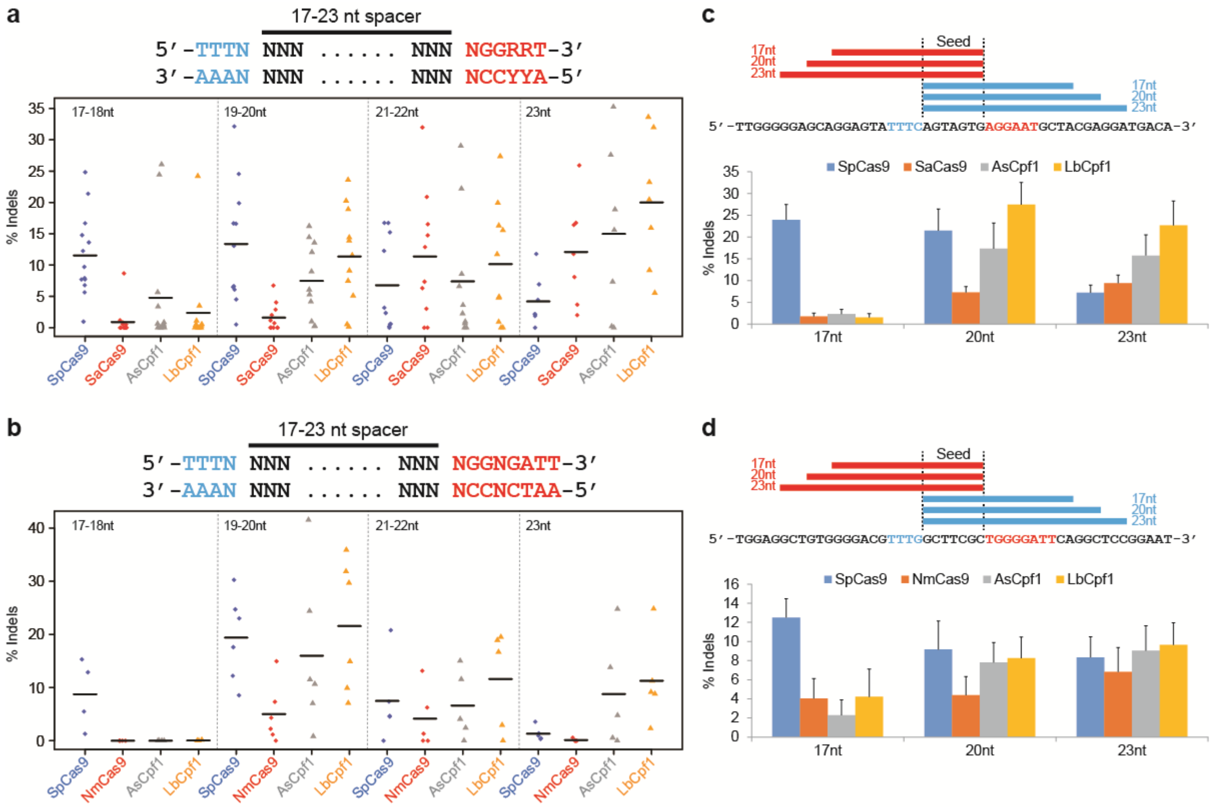

Figure S5 Evaluation of various CRISPR-Cas systems in NHEJ-mediated genome editing using matched spacers or matched seeds.

a, b Summary of matched target site activities (see Figure S3) for SpCas9, either **a** SaCas9 or **b** NmCas9, AsCpf1, and LbCpf1 based on T7E1 assays. Each horizontal bar indicates the mean of the editing activities for the indicated enzyme and range of spacer lengths.

c, d Extent of genome modifications at a target locus in the **c** CACNA1D or **d** PPP1R12C gene whereby the Cas9 and Cpf1 nucleases had overlapping seed regions. Three different spacer lengths (17nt, 20nt, and 23nt) were tested. The cells were harvested 24 hours after transfection and the editing efficiencies were determined by T7E1 assays. Data represent mean ± s.e.m (n ≥ 6).

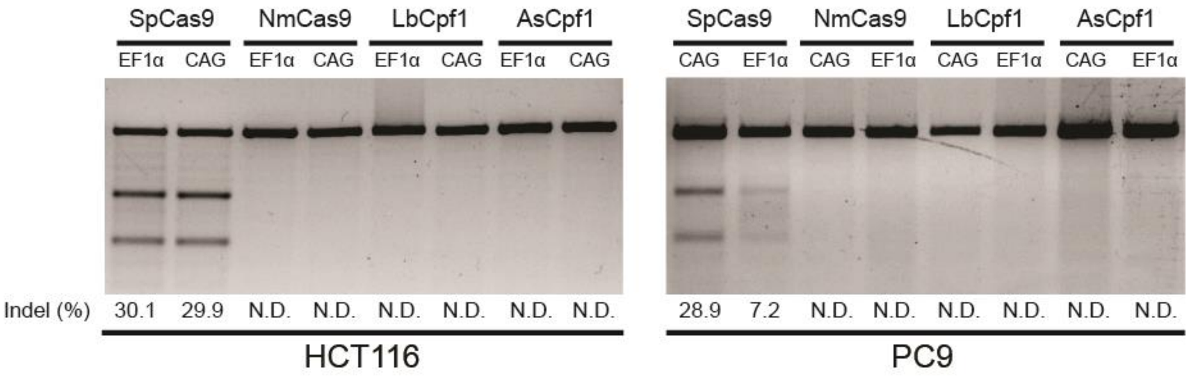

Figure S6 We observed in HEK293T cells that SpCas9 was the only Cas nuclease that exhibited robust editing activity with short 17nt spacers. To confirm the results, we targeted the B1 (17nt) site located within the ALK gene in other cell lines, namely HCT116 and PC9. All cells were harvested at 24 hours after transfection. From T7E1 cleavage assays, we again found that only SpCas9 was able to modify the genome robustly at this genomic locus, thereby verifying our observation in the HEK293T cell line. (N.D.: not detected)

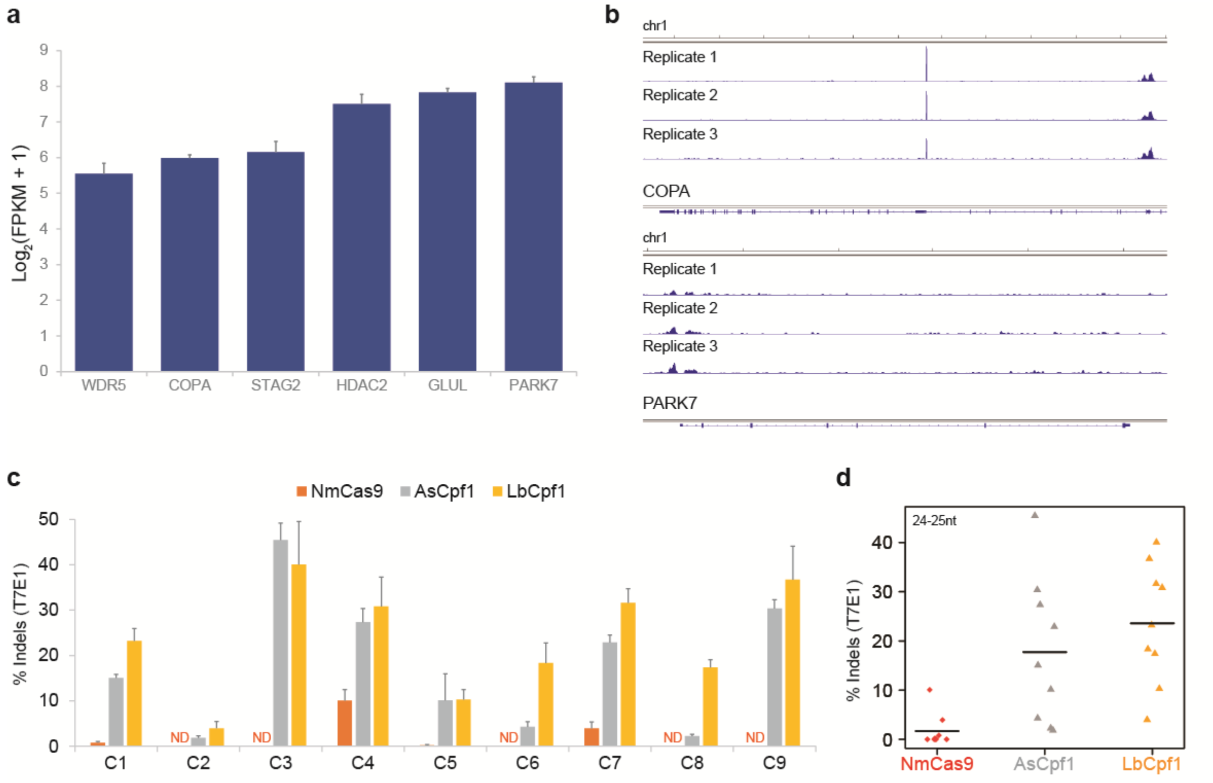

Figure S7 Editing activities of NmCas9 and Cpf1 nucleases at target sites of lengths 24-25nt.

a Nine additional target sites (group C) were selected in six different highly expressed genes (WDR5, COPA, STAG2, HDAC2, GLUL, and PARK7) based on RNA-seq experiments. Data represent mean ± s.e.m (n = 6).

b H3K27ac ChIP-seq data for the COPA and PARK7 genes. The ChIP-seq peaks for STAG2 are shown in Figure S2b, while the peaks for the remaining genes (WDR5, HDAC2, and GLUL) are provided in Figure S9c.

c Bar graph showing the editing activity of NmCas9 and the two Cpf1 nucleases at the nine newly selected target sites, which take the form TTTN-$N_{24\text{-}25}$-NNNNGATT (see Table S5). The cells were harvested 24 hours after transfection and then the editing frequencies were quantified by T7E1 assays. Data represent mean ± s.e.m (n ≥ 5). (N.D.: not detected)

d Strip chart summarizing the editing efficiencies of NmCas9, AsCpf1, and LbCpf1 at perfectly matched target sites of longer lengths (24-25nt).

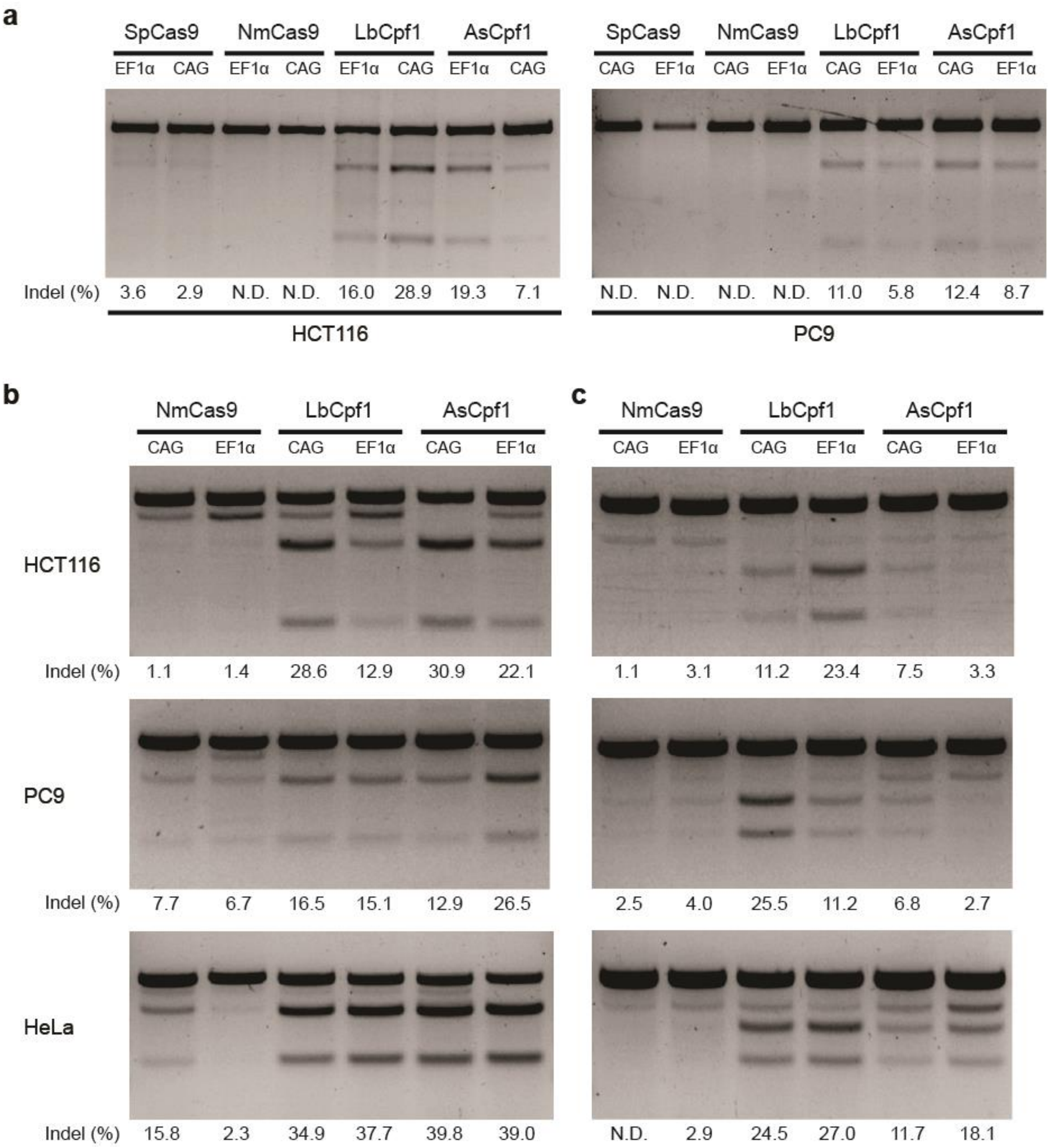

Figure S8 Confirming the low editing activity of NmCas9 in multiple human cell lines. We found that NmCas9 failed to edit the HEK293T genome at many of the tested sites, while other nucleases were able to generate indels. To confirm the results, we targeted three distinct sites in various alternative cell lines, namely **a** B5 (21nt) in the ALK gene, **b** C4 (24nt) in the STAG2 gene, and **c** C1 (24nt) in the WDR5 gene. From T7E1 assays, we observed that NmCas9 again produced weaker cleavage bands than the other Cas enzymes at the three sites in all the additional cell lines tested, thereby verifying our earlier results in HEK293T cells.

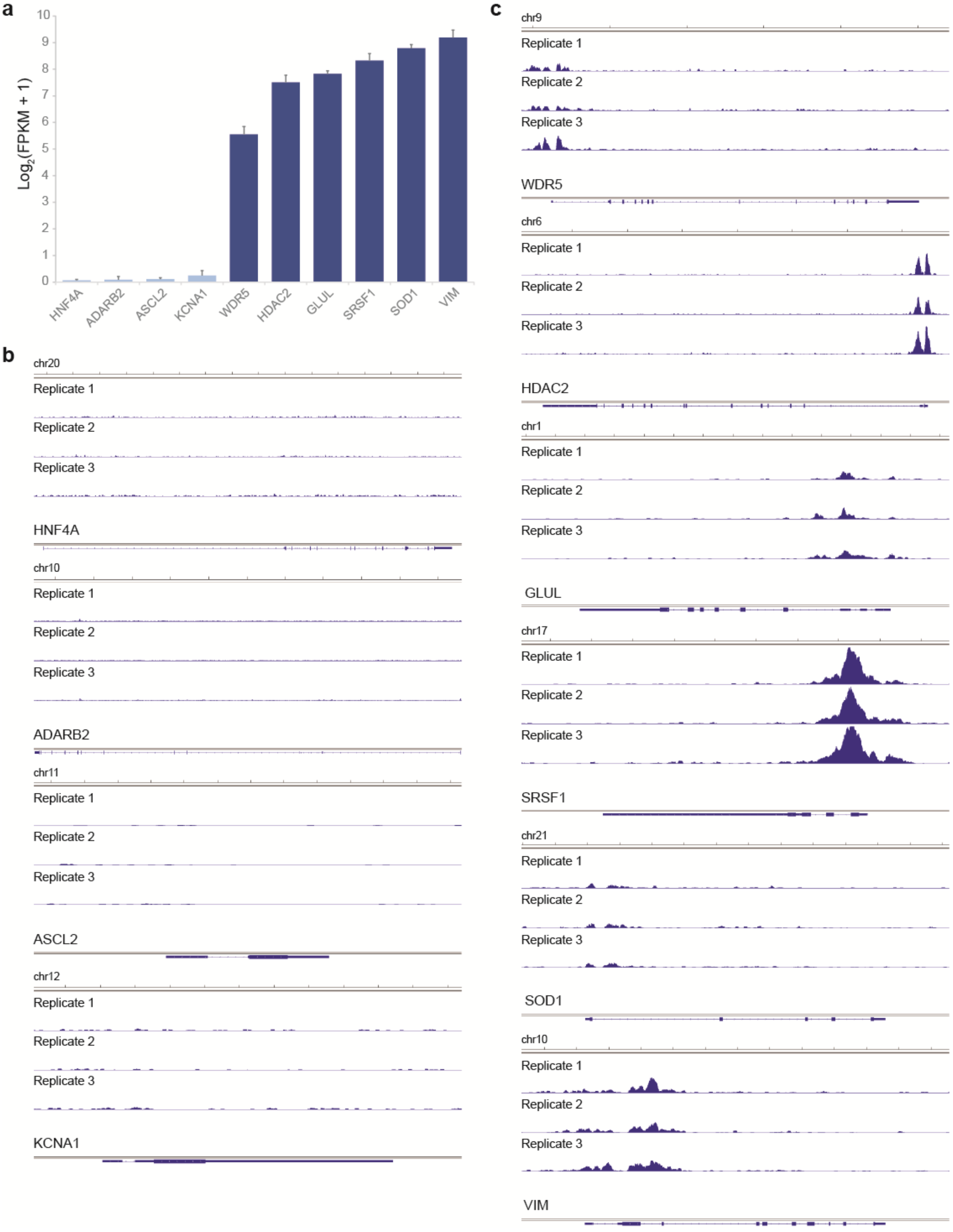
a
b
c
Log2(FPKM + 1)
HNF4A
ADARB2
ASCL2
KCNA1
WDR5
HDAC2
GLUL
SRSF1
SOD1
VIM
chr20
chr10
chr11
chr12
chr9
chr6
chr1
chr17
chr21
chr10
Replicate 1
Replicate 2
Replicate 3

Figure S9 Selection of new N_{21}NGGRRT target sites (group D).

a To better characterize the editing activity of SaCas9, we selected six new target sites in four lowly expressed genes (HNF4A, ADARB2, ASCL2, and KCNA1) (light blue bars) as well as 12 new target sites in six highly expressed genes (WDR5, HDAC2, GLUL, SRSF1, SOD1, and VIM) (dark blue bars). The expression levels of all the nine genes were obtained from RNA-seq experiments. Data represent mean ± s.e.m (n = 6).

b H3K27ac ChIP-seq data for the four lowly expressed genes. No obvious peak could be seen in the plots.

c H3K27ac ChIP-seq data for the six highly expressed genes. We observed clear ChIP-seq peaks for all these genes.

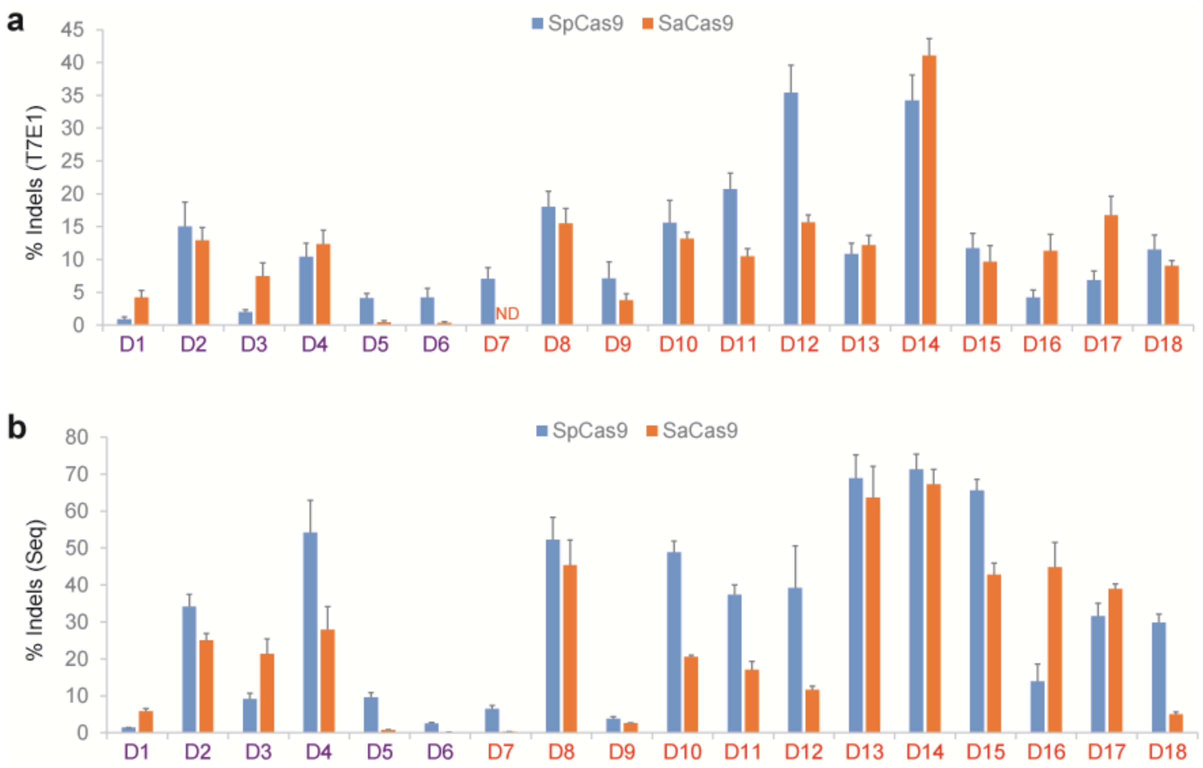

Figure S10 Editing efficiencies at group D target sites.

The activity of SpCas9 and SaCas9 was measured either by **a** the T7E1 cleavage assay or by **b** Illumina deep sequencing experiments 24 hours post-transfection. D1-D6 are in lowly expressed genes, while D7-D18 are in highly expressed genes (see Table S6). Data represent mean ± s.e.m ($n \geq 4$). (N.D.: not detected)

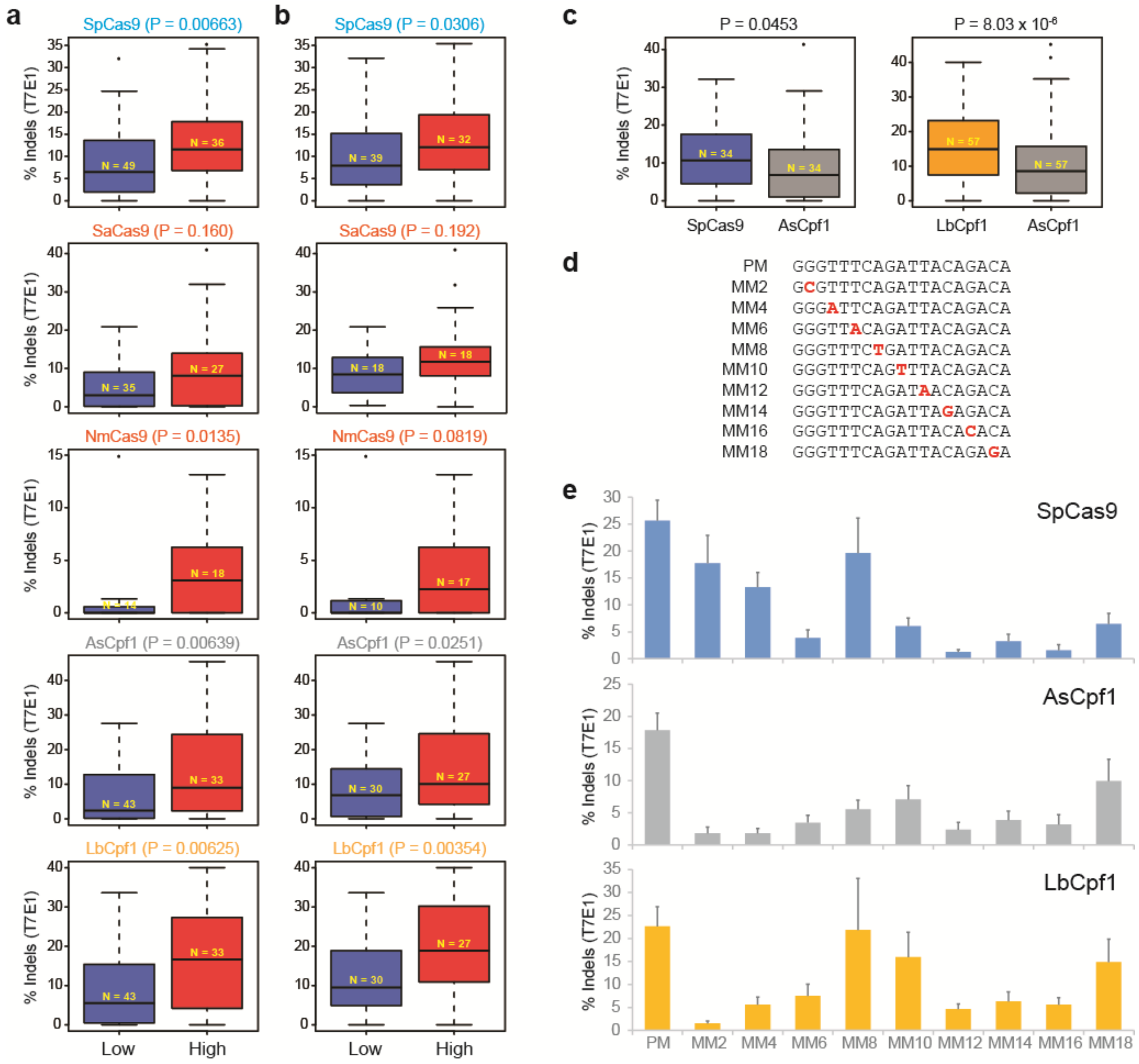

Figure S11 Relationship of DNA cleavage efficiency with gene expression and target specificity.

a Impact of gene expression on editing efficiency. We divided the target sites into those that occur in lowly expressed genes (FPKM < 25, blue boxplots) and those that occur in highly expressed genes (FPKM ≥ 25, red bloxplots) using our RNA-seq data. The FPKM value of 25 was chosen to divide the target sites into two groups of roughly equal sizes for the five Cas nucleases. Here, all sgRNAs were considered in the analysis. Overall, we found from our T7E1 assays that SpCas9, AsCpf1, and LbCpf1 were able to edit highly expressed genes more efficiently than lowly expressed genes ($P < 0.05$, Wilcoxon rank sum test). In contrast, the performance of SaCas9 was not influenced by gene expression. For NmCas9, its activity appeared to be dependent on gene expression as well, but this may be due to the numerous zero values resulting from non-optimal sgRNAs.

b Similar analysis to **a**, except that only sgRNAs of optimal lengths were considered. In the current study, we set the optimal lengths of SpCas9 as 17-22nt inclusive, SaCas9 as ≥ 21nt, NmCas9 as ≥ 19nt, AsCpf1 as ≥ 19nt, and LbCpf1 as ≥ 19nt. Again, the activity of SpCas9, AsCpf1, and LbCpf1 showed a significant dependence on gene expression ($P < 0.05$, Wilcoxon rank sum test). In contrast, the performance of the two smaller Cas enzymes, SaCas9 and NmCas9, was less affected by expression levels of the targeted genes.

c Comparison of AsCpf1 with either SpCas9 (left boxplot) or LbCpf1 (right boxplot). Only sgRNAs of the optimal lengths for SpCas9 and the Cpf1 nucleases (19-22nt inclusive) were considered. From T7E1 assays, we found that the editing activity of AsCpf1 was significantly lower than both SpCas9 and LbCpf1 ($P < 0.05$, Wilcoxon rank sum test).

d To assess the specificities of SpCas9, AsCpf1, and LbCpf1, we examined the tolerance of these enzymes to single mismatches along the spacer targeting the A17 site in the NF1 gene. Red letters indicate the mutated bases.

e Using the spacers indicated in **d**, we determined the editing activities of SpCas9, AsCpf1, and LbCpf1 by T7E1 assays. The cells were harvested 24 hours after transfection. For all three nucleases, we observed an increased tolerance to mismatches around the middle of the spacer. Importantly, while SpCas9 and LbCpf1 exhibited higher cleavage efficiencies than AsCpf1 with a perfect matched (PM) spacer, they also showed an overall higher tolerance to mismatches between the spacer and the target DNA. Data represent mean ± s.e.m ($n \geq 5$).

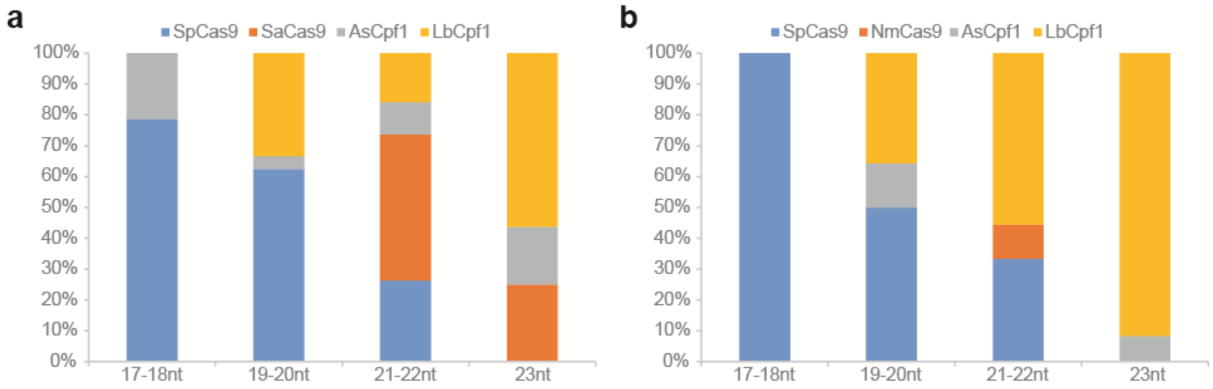

Figure S12 Identification of Cas enzymes with the highest cleavage efficiencies at different spacer lengths. At each target site, we asked which of the four nucleases exhibited the highest editing activity. We then determined the total number of sites that each nuclease emerged as the best performing enzyme.

a Percentages of the set of Group A target sites (of the specified lengths), including those in the CACNA1D gene, whereby each indicated enzyme generated the largest amount of indels. SpCas9 was the best performing nuclease for spacers that were 17-20nt long, while SaCas9 and LbCpf1 were the best performing nucleases for spacers that were 21-23nt long.

b Percentages of the set of Group B target sites (of the specified lengths), including those in the PPP1R12C gene, whereby each indicated enzyme generated the largest amount of indels. SpCas9 was again the best performing nuclease for 17-20nt long spacers, while LbCpf1 remained the best performing nuclease for 21-23nt long spacers. Notably, there was no target site of any length whereby NmCas9 exhibited the highest cleavage efficiency.

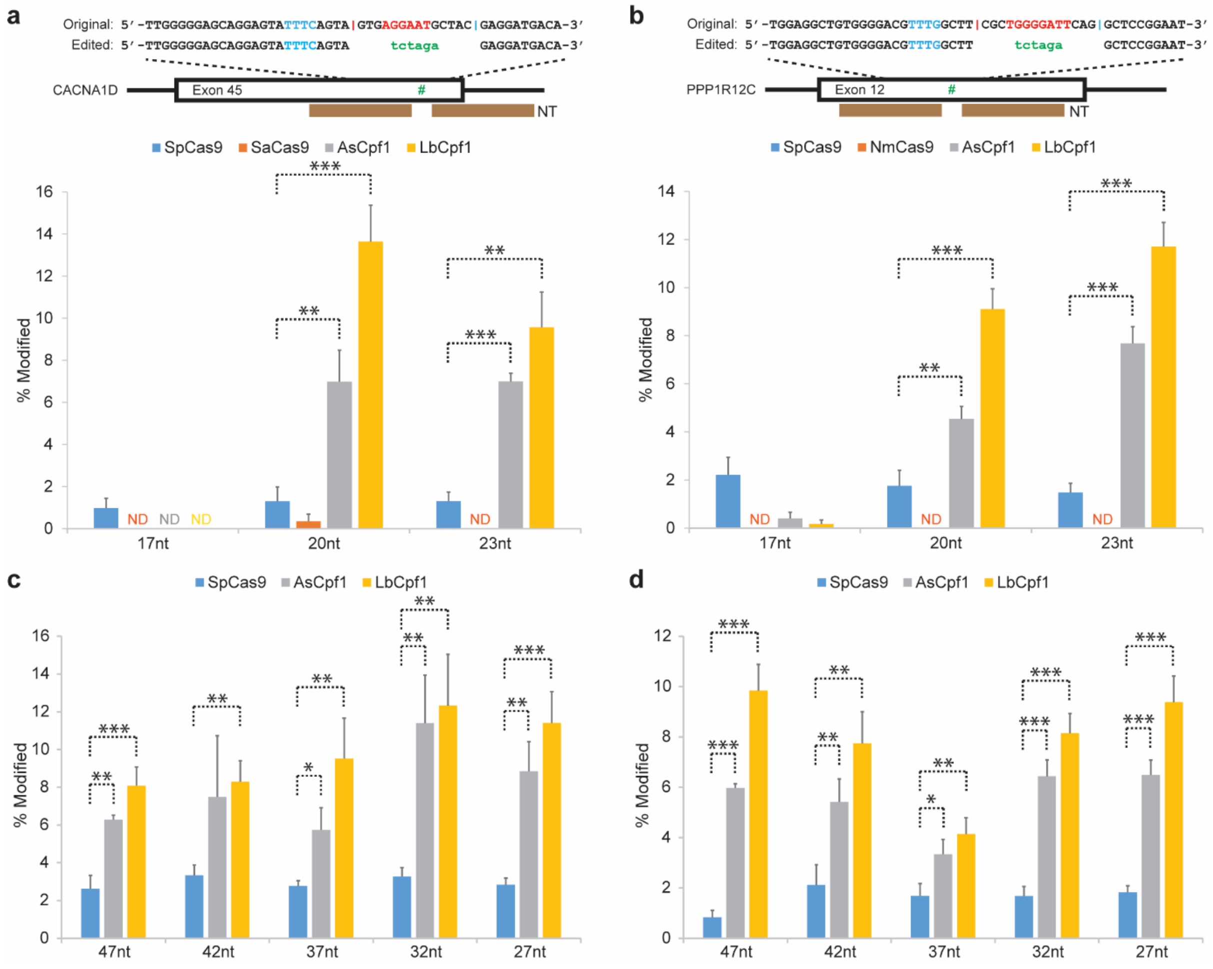

Figure S13 Evaluation of various CRISPR-Cas systems in HDR-mediated genome editing using ssODN donor templates.

a, b Extent of XbaI restriction site (depicted in green) insertion into a coding exon of the **a** CACNA1D or **b** PPP1R12C gene. The brown horizontal bars represent the 47nt homology arms of the donor template and NT indicates that the donor is of the non-target strand sequence. Three different spacer lengths (17nt, 20nt, and 23nt) were tested. The cells were harvested 72 hours after transfection and the gene targeting efficiencies were determined by RFLP analysis. Data represent mean ± s.e.m (n = 6). (N.D.: not detected)

c, d Extent of precise gene editing by SpCas9, AsCpf1, and LbCpf1 when ssODNs of different homology arm lengths (27-47nt) were used together with 20nt spacers targeting **c** CACNA1D or **d** PPP1R12C. The cells were harvested 72 hours after transfection and the gene targeting efficiencies were determined by RFLP analysis. Data represent mean ± s.e.m ($n \geq 6$). (* $P < 0.05$, ** $P < 0.01$, *** $P < 0.0001$; Student's t-test)

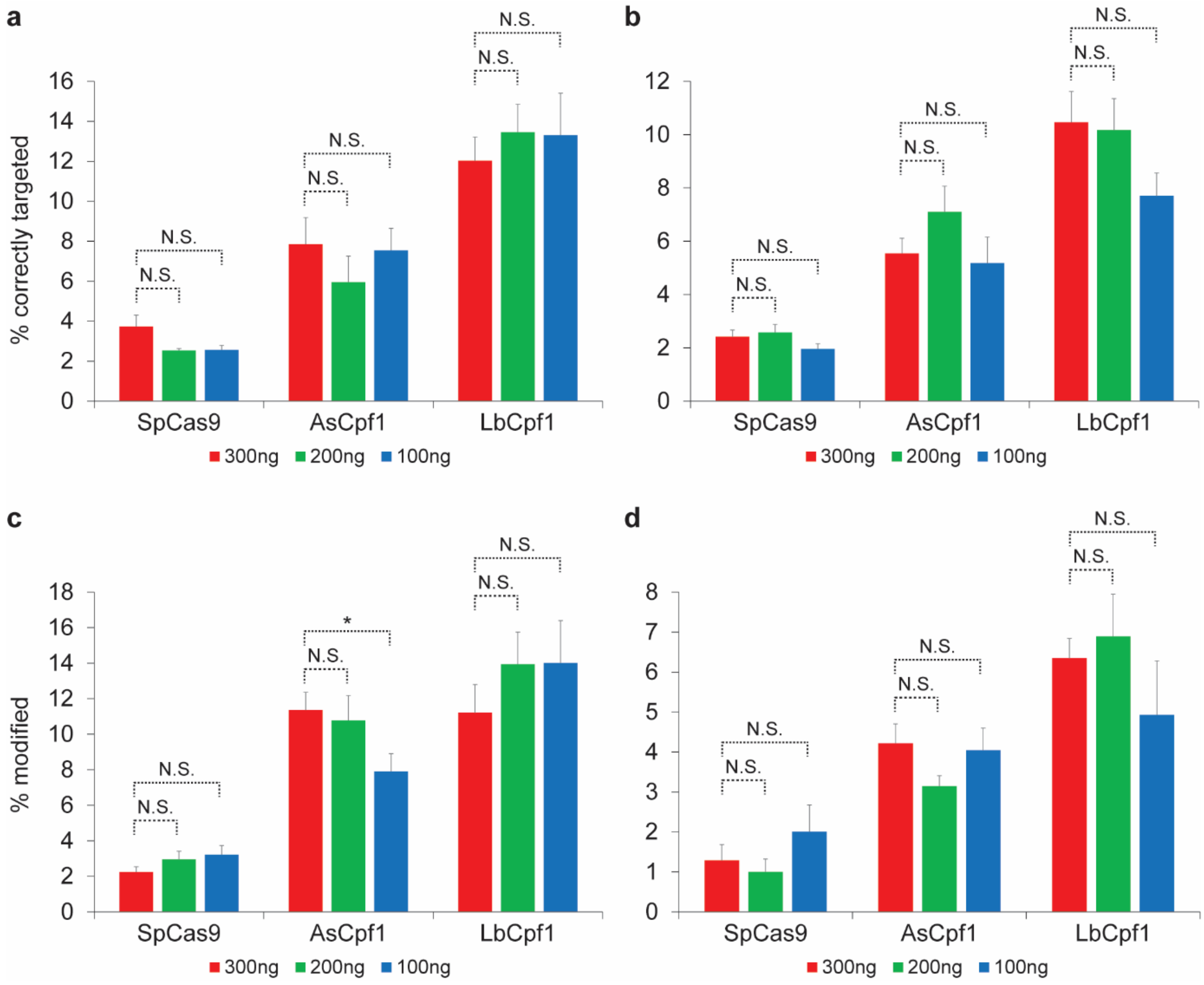

Figure S14 Extent of precise gene targeting with different amounts of ssODN donors. We sought to determine whether the amount of donor templates transfected into the cell may affect the rate of restriction site integration at the **a, c** CACNA1D and **b, d** PPP1R12C genomic loci by SpCas9, AsCpf1, and LbCpf1. 100-300ng of single-stranded DNA were introduced into the cell together with the relevant CRISPR plasmids. The HDR rates were quantified by either **a, b** deep sequencing experiments or **c, d** RFLP analysis. Overall, we observed similar HDR frequencies for all three quantities of ssODNs tested. (* $P < 0.05$, N.S.: not significant; Student's t-test)

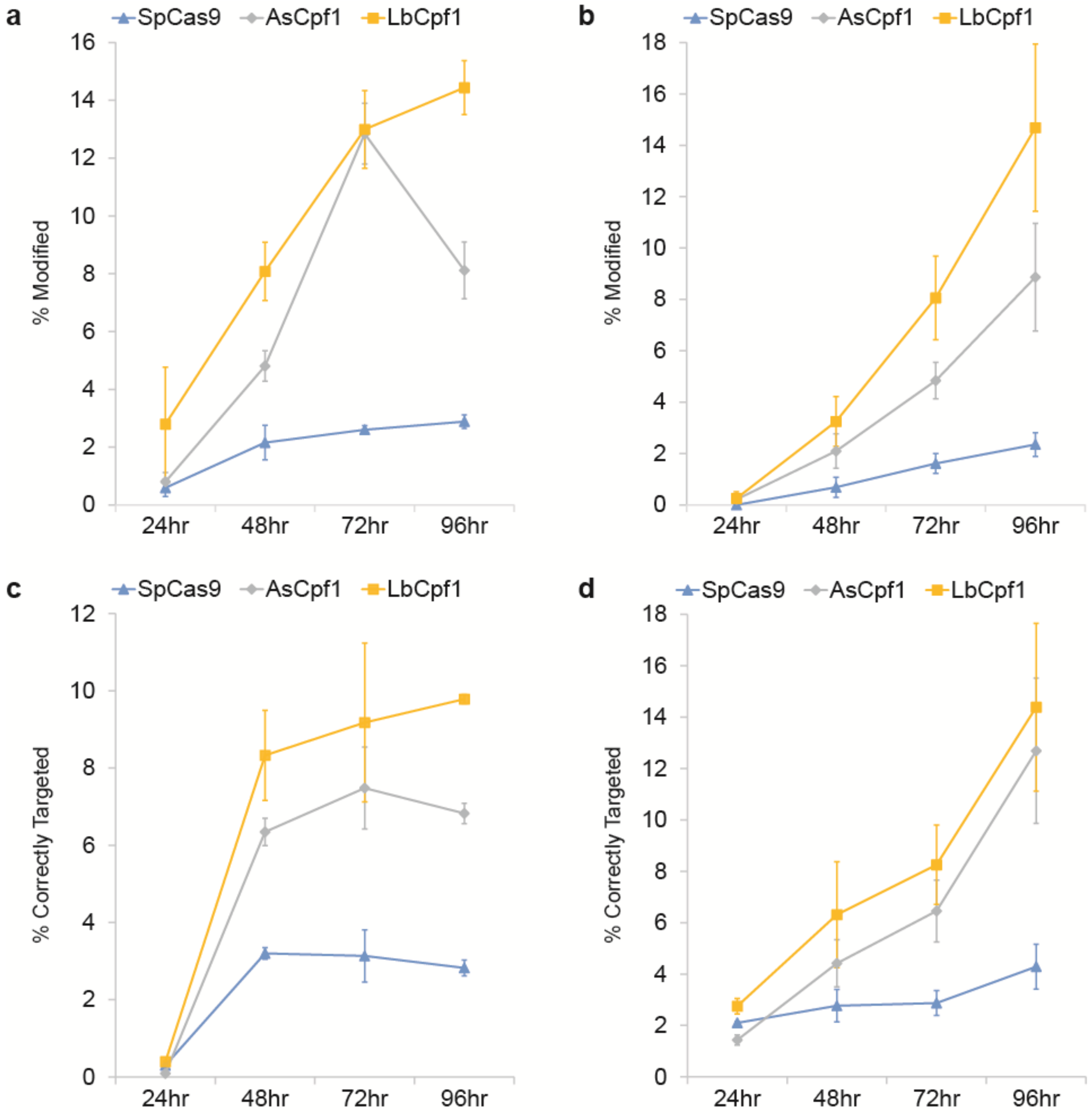

Figure S15 Timecourse study of precise genome editing activity at two exemplary sites. Extent of precise gene targeting over time as determined by **a, b** RFLP analysis or by **c, d** Illumina deep sequencing at the two genomic loci with overlapping seed regions for Cas9 and Cpf1 nucleases, namely **a, c** CACNA1D and **b, d** PPP1R12C. Donor ssODNs with 47nt homology arm lengths were used together with 20nt spacers. Overall, the amount of genomic DNA with XbaI integrated into either the **a, c** CACNA1D or **b, d** PPP1R12C gene increased with time. Data represent mean ± s.e.m ($n \geq 2$).

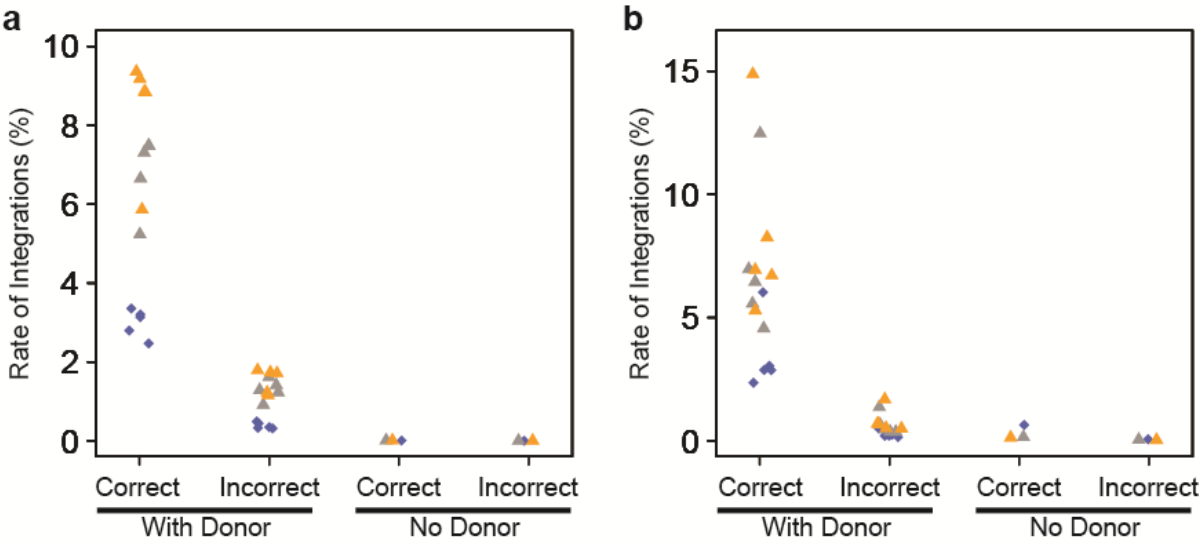

Figure S16 Correct versus incorrect integrations of restriction sites.

We utilized our deep sequencing data to investigate the rate of erroneous incorporations of XbaI into the **a** CACNA1D or **b** PPP1R12C genomic locus. As a baseline, we also determined the extent of XbaI integrations in NHEJ-mediated editing experiments when no donor template was provided. Blue diamond data points indicate SpCas9, gray triangle data points indicate AsCpf1, and orange triangle data points indicate LbCpf1.

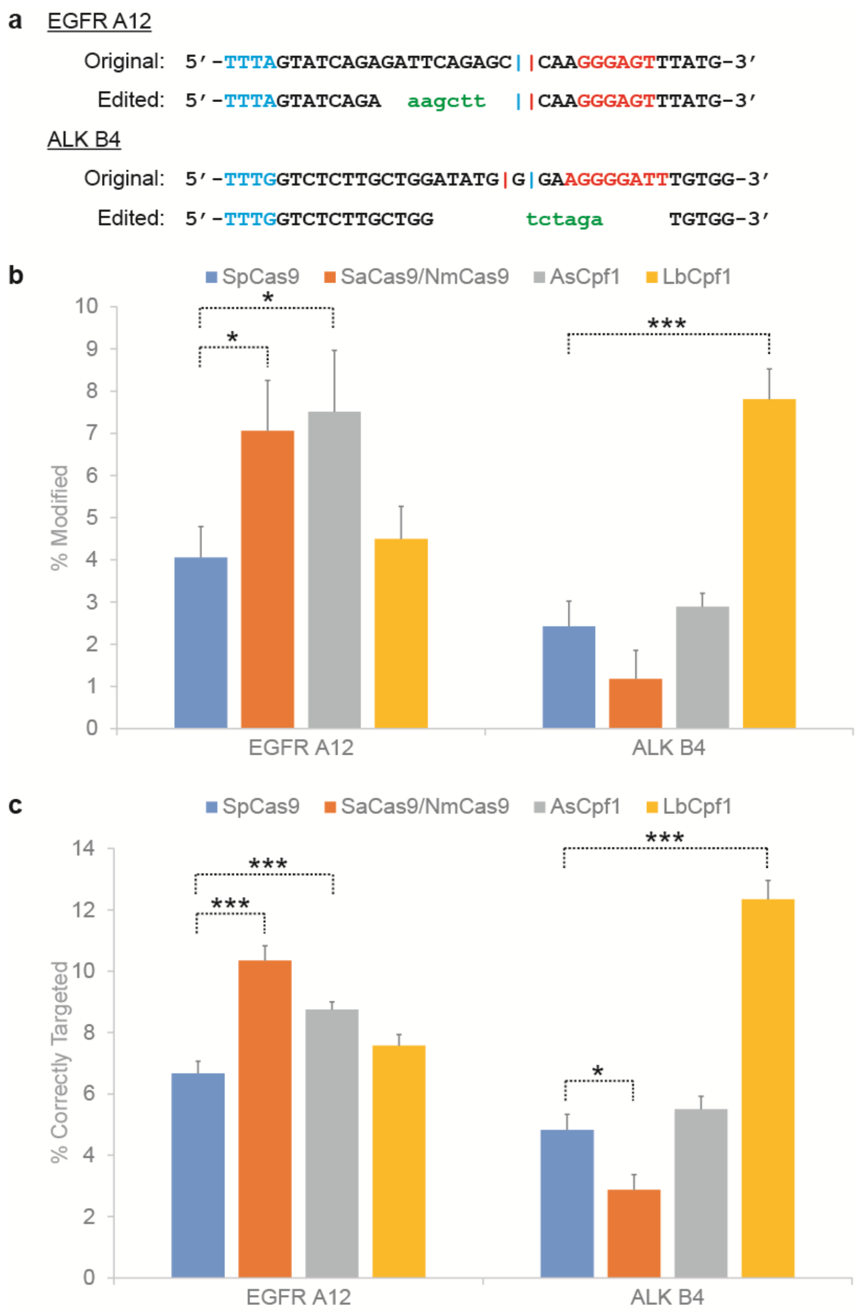

Figure S17 Evaluation of various CRISPR-Cas systems in HDR-mediated editing of intronic regions using ssODNs with 47nt homology arms.

a Intended DNA changes at the A12 and B4 target sites in the EGFR and ALK genes respectively. Each red vertical line indicates the cleavage site of Cas9 nucleases, which occurs 3 basepairs (bp) upstream of their PAM. Each blue vertical line indicates the cleavage site of Cpf1 nucleases on one DNA strand, which occurs 18nt downstream of their PAM.

b, c Extent of inserting a HindIII or XbaI restriction site into the A12 or B4 target site respectively. Donor ssODNs with 47nt homology arm lengths were used. The cells were harvested for either **b** RFLP or **c** deep sequencing analysis 72hr post-transfection. Data represent mean ± s.e.m (n ≥ 6). (* $P < 0.05$, ** $P < 0.01$, *** $P < 0.0001$; Student’s t-test)

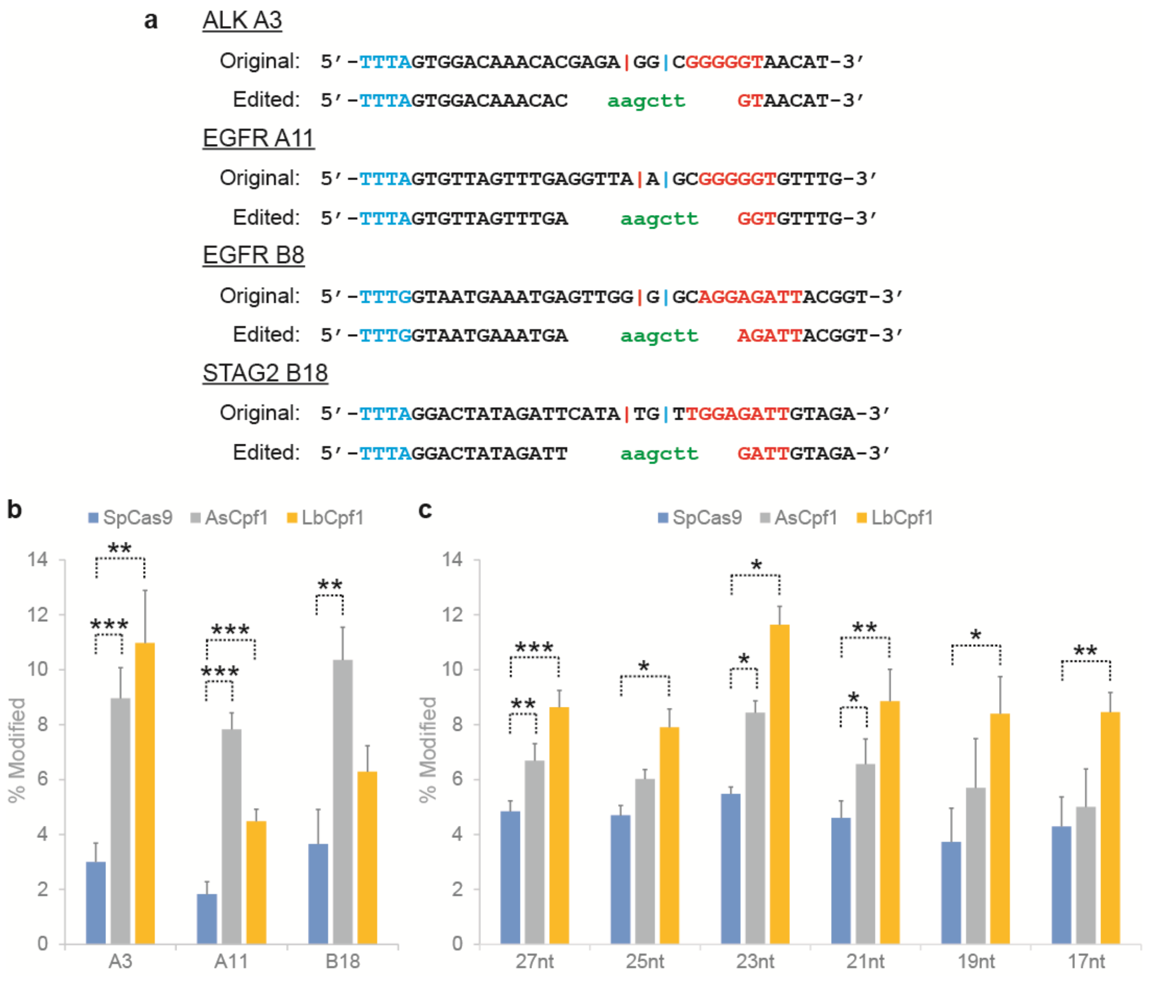

Figure S18 Evaluation of various CRISPR-Cas systems in HDR-mediated editing of intronic regions using ssODNs with 27nt or shorter homology arms.

a Intended DNA changes at the A3 (in ALK), A11 (in EGFR), B8 (in EGFR), and B18 (in STAG2) target sites. Each red vertical line indicates the cleavage site of Cas9 nucleases, which occurs 3bp upstream of their PAM. Each blue vertical line indicates the cleavage site of Cpf1 nucleases on one DNA strand, which occurs 18nt downstream of their PAM. The HindIII restriction site is indicated in green.

b Extent of incorporating the HindIII recognition sequence into the A3, A11, or B8 target sites. Donor ssODNs with 27nt homology arm lengths were used. The cells were harvested for RFLP analysis 72hr post-transfection. The Cpf1 nucleases consistently gave more digested products than SpCas9. Data represent mean ± s.e.m ($n \geq 6$). (** $P < 0.01$, *** $P < 0.001$; Student's t-test)

c Extent of precise gene editing by SpCas9, AsCpf1, and LbCpf1 at the B8 locus when ssODNs of different homology arm lengths (17-27nt inclusive) were used. The cells were harvested for RFLP analysis 72hr post-transfection. Data represent mean ± s.e.m ($n \geq 2$). (* $P < 0.05$, ** $P < 0.01$, *** $P < 0.001$; Student's t-test)

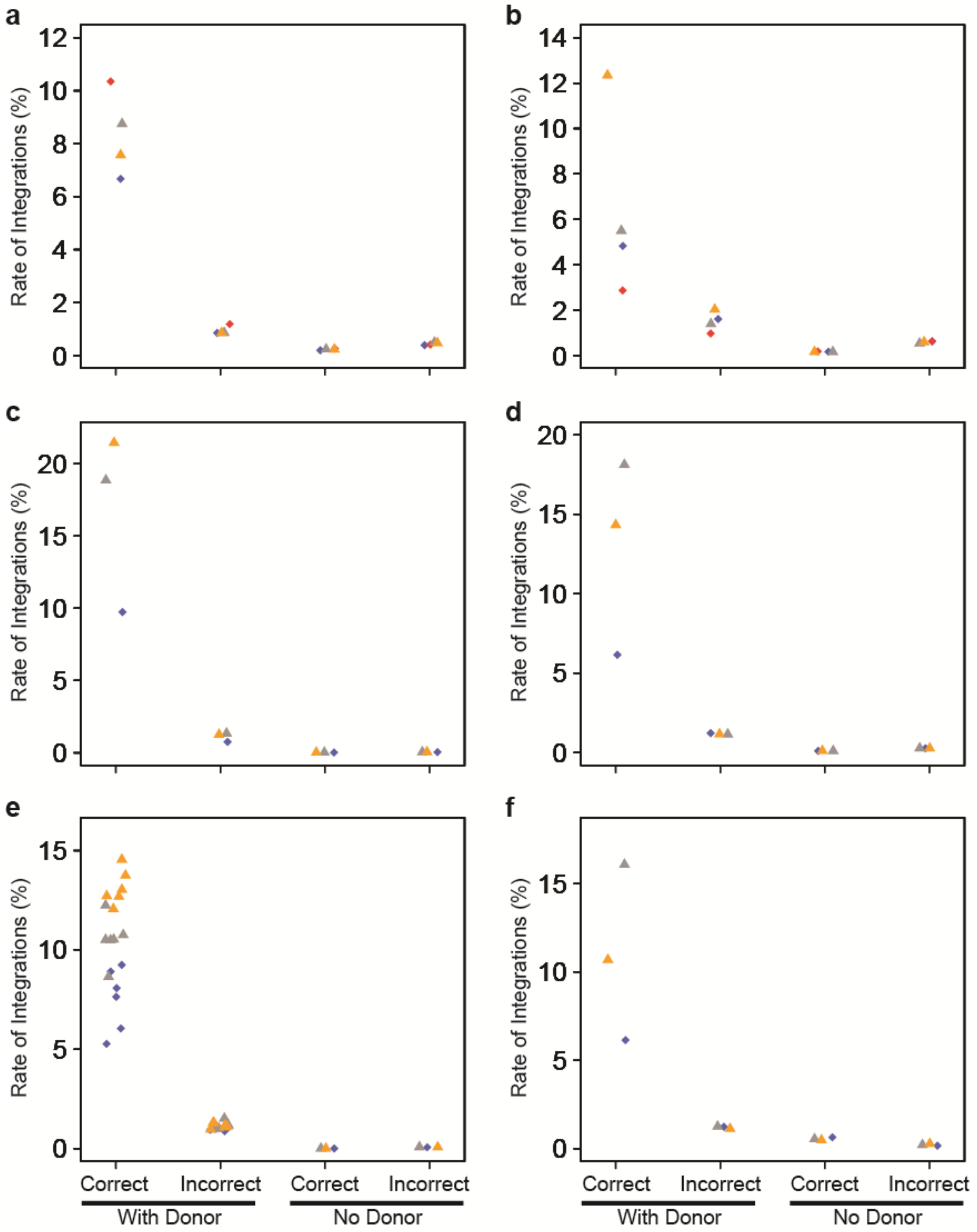

Figure S19 Correct versus incorrect integrations of restriction sites.

We utilized our deep sequencing data to investigate the rate of erroneous restriction site incorporations into the **a** A12 (in EGFR), **b** B4 (in ALK), **c** A3 (in ALK), **d** A11 (in EGFR), **e** B8 (in EGFR), or **f** B18 (in STAG2) target locus. As a baseline, we also determined the extent of restriction site integrations in NHEJ-mediated editing experiments when no donor template was provided. Blue diamond data points indicate SpCas9, red diamond data points indicate either SaCas9 or NmCas9, gray triangle data points indicate AsCpf1, and orange triangle data points indicate LbCpf1.

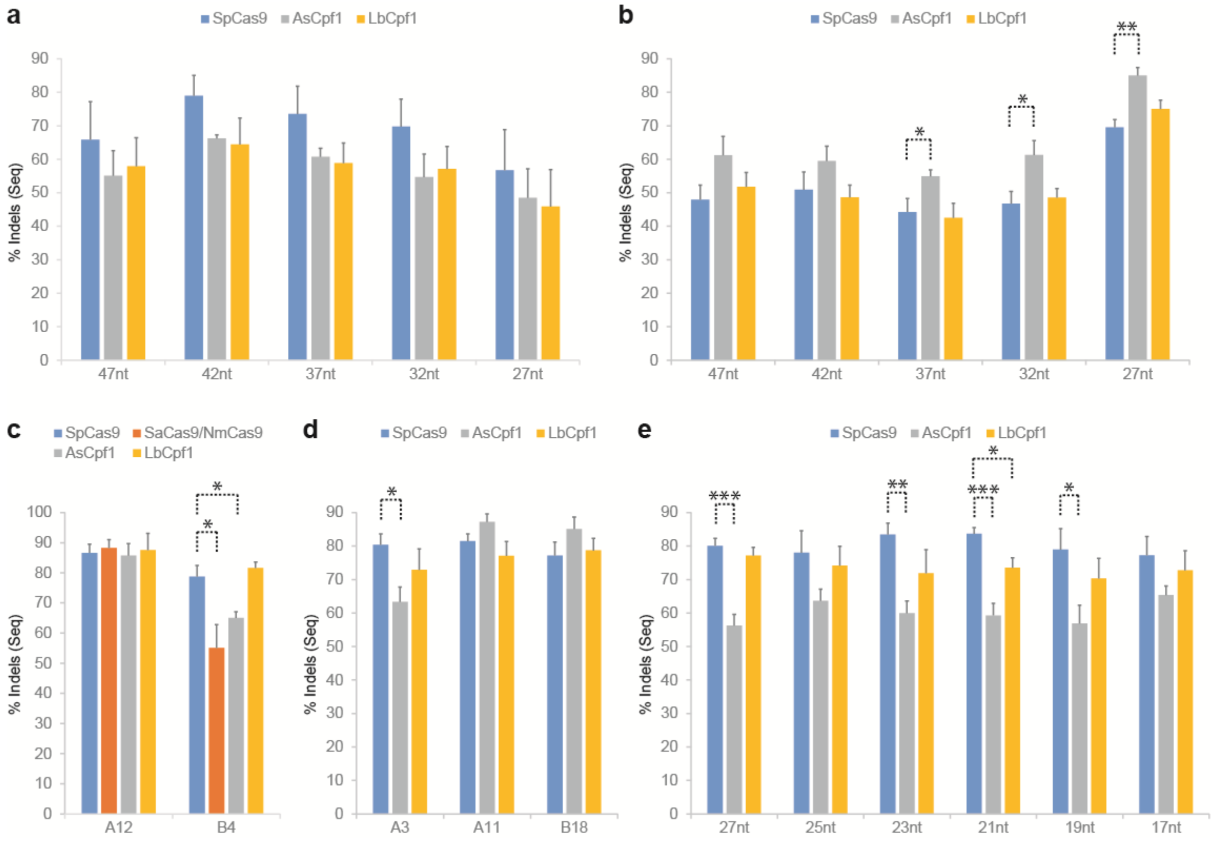

Figure S20 Measurements of cleavage rates in editing experiments with ssODNs used. Extent of indel formation at **a** CACNA1D, **b** PPP1R12C, **c** A12 (in EGFR) and B4 (in ALK), **d** A3 (in ALK), A11 (in EGFR), and B18 (in STAG2), and **e** B8 (in EGFR) target loci, as quantified by Illumina deep sequencing. Overall, we found that SpCas9 was as efficient as the other Cas endonucleases at cleaving the selected target sites in the human genome. Data represent mean ± s.e.m ($n \geq 3$). (* $P < 0.05$, ** $P < 0.01$, *** $P < 0.001$; Student's t-test)

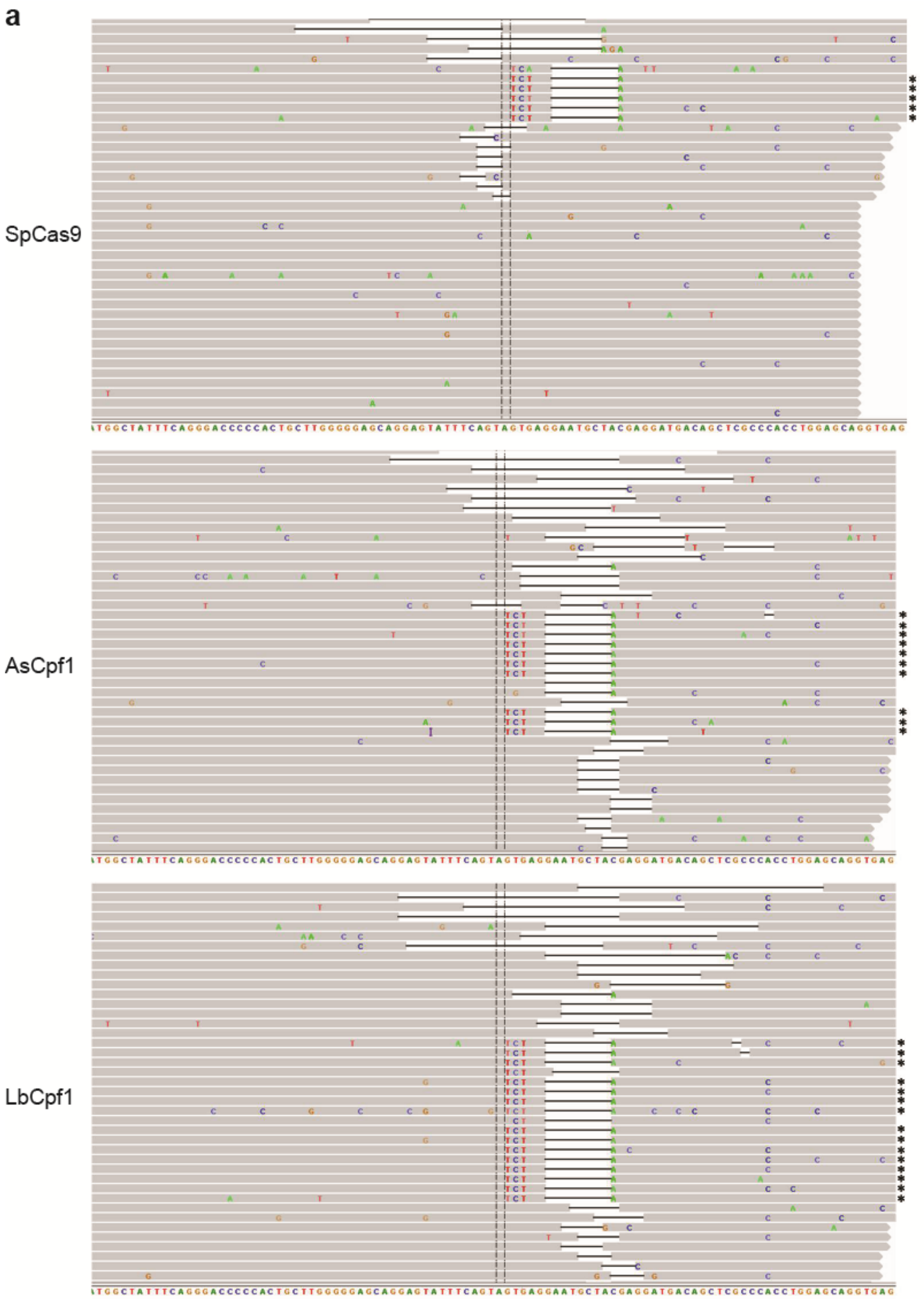
a
SpCas9
AsCpf1
LbCpf1

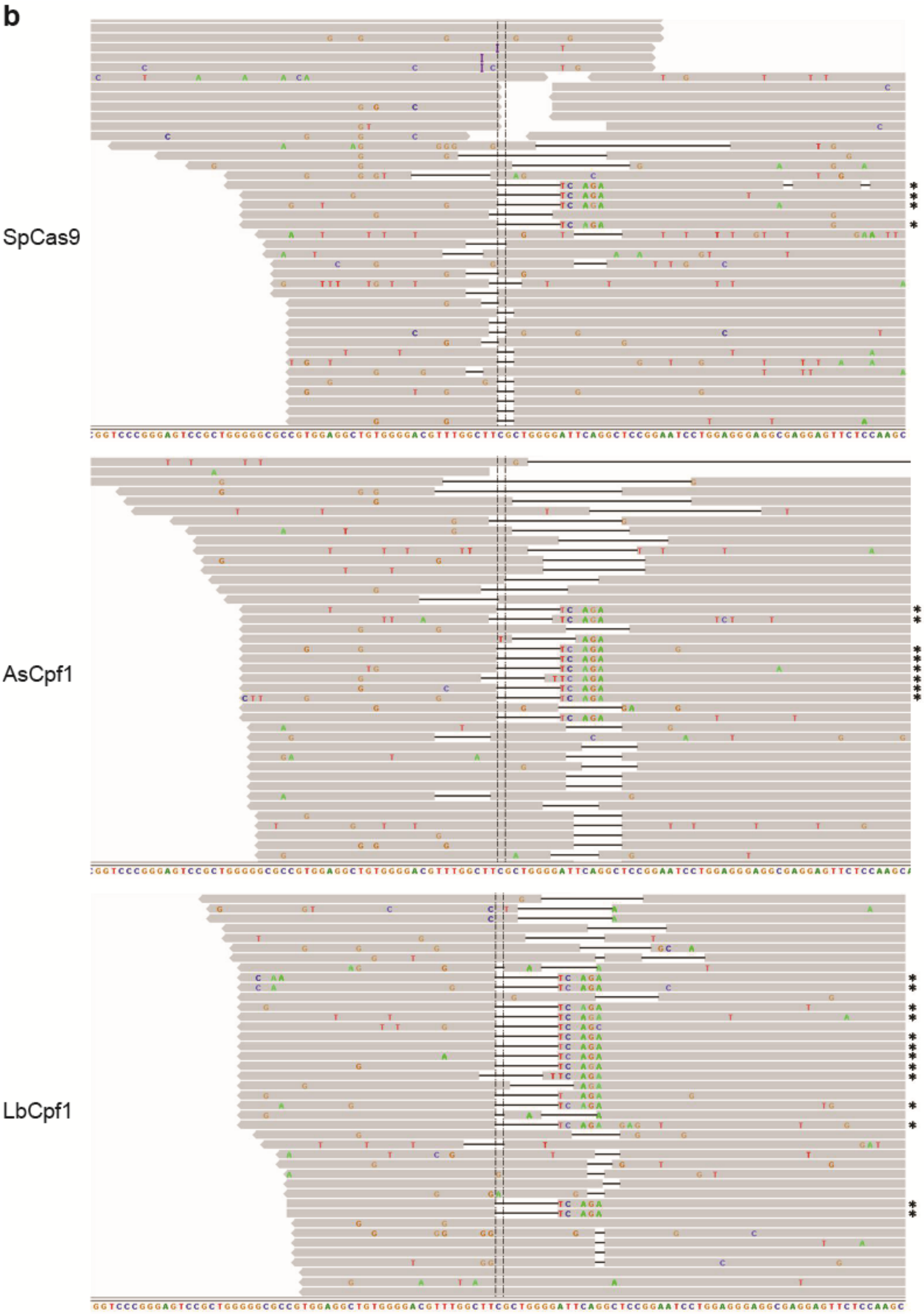
b
SpCas9
AsCpf1
LbCpf1

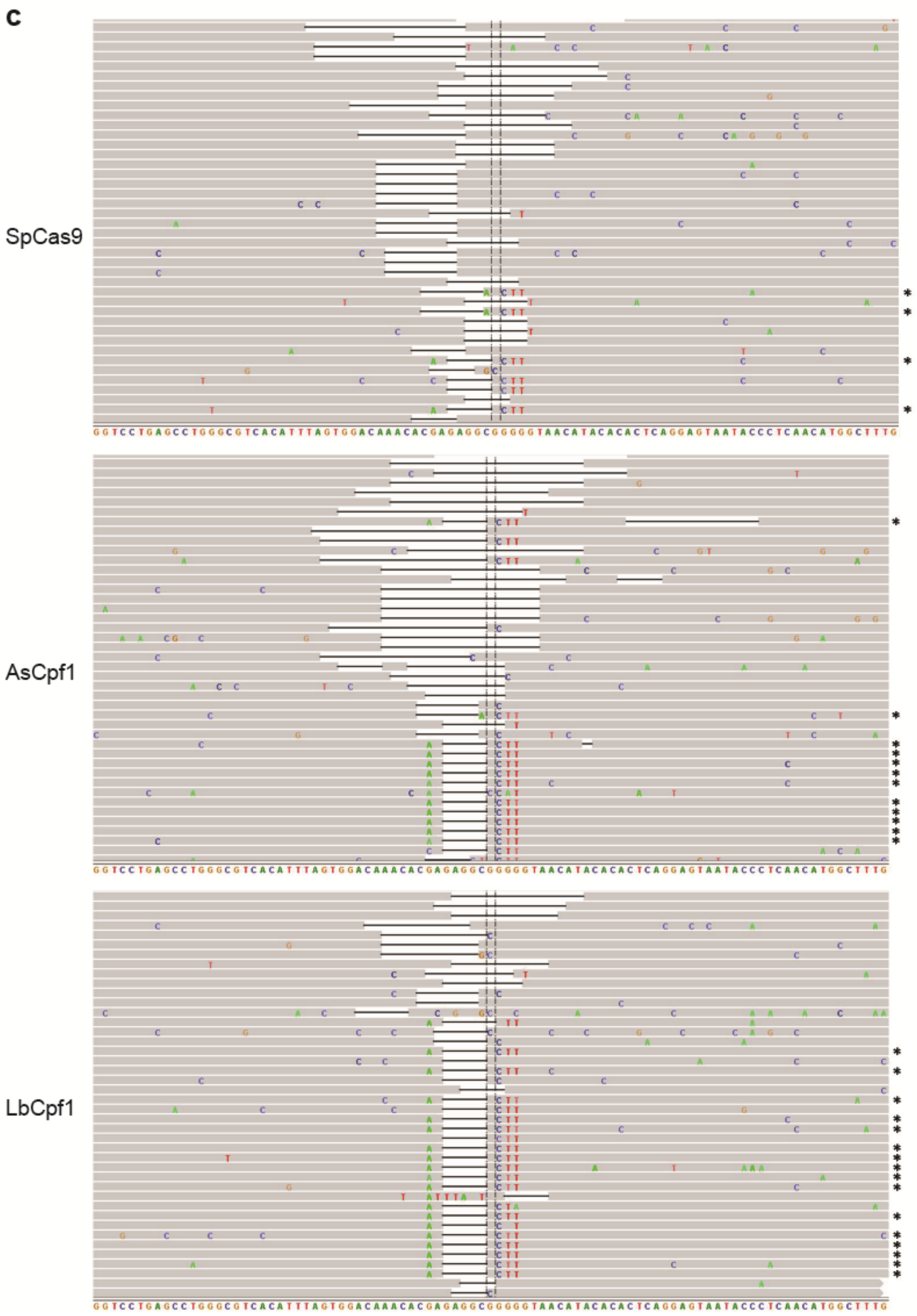
c
SpCas9
AsCpf1
LbCpf1

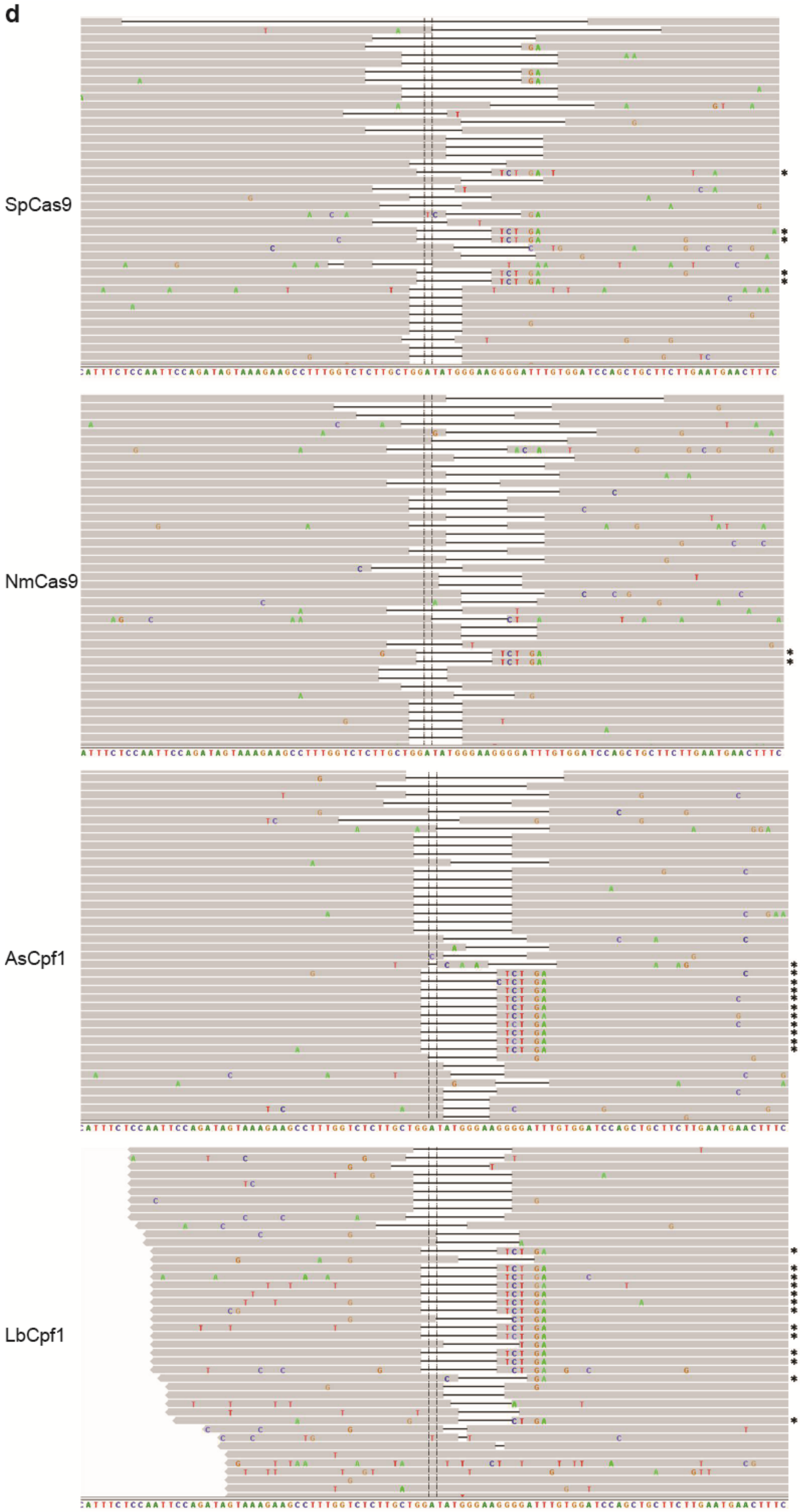
d
SpCas9
NmCas9
AsCpf1
LbCpf1

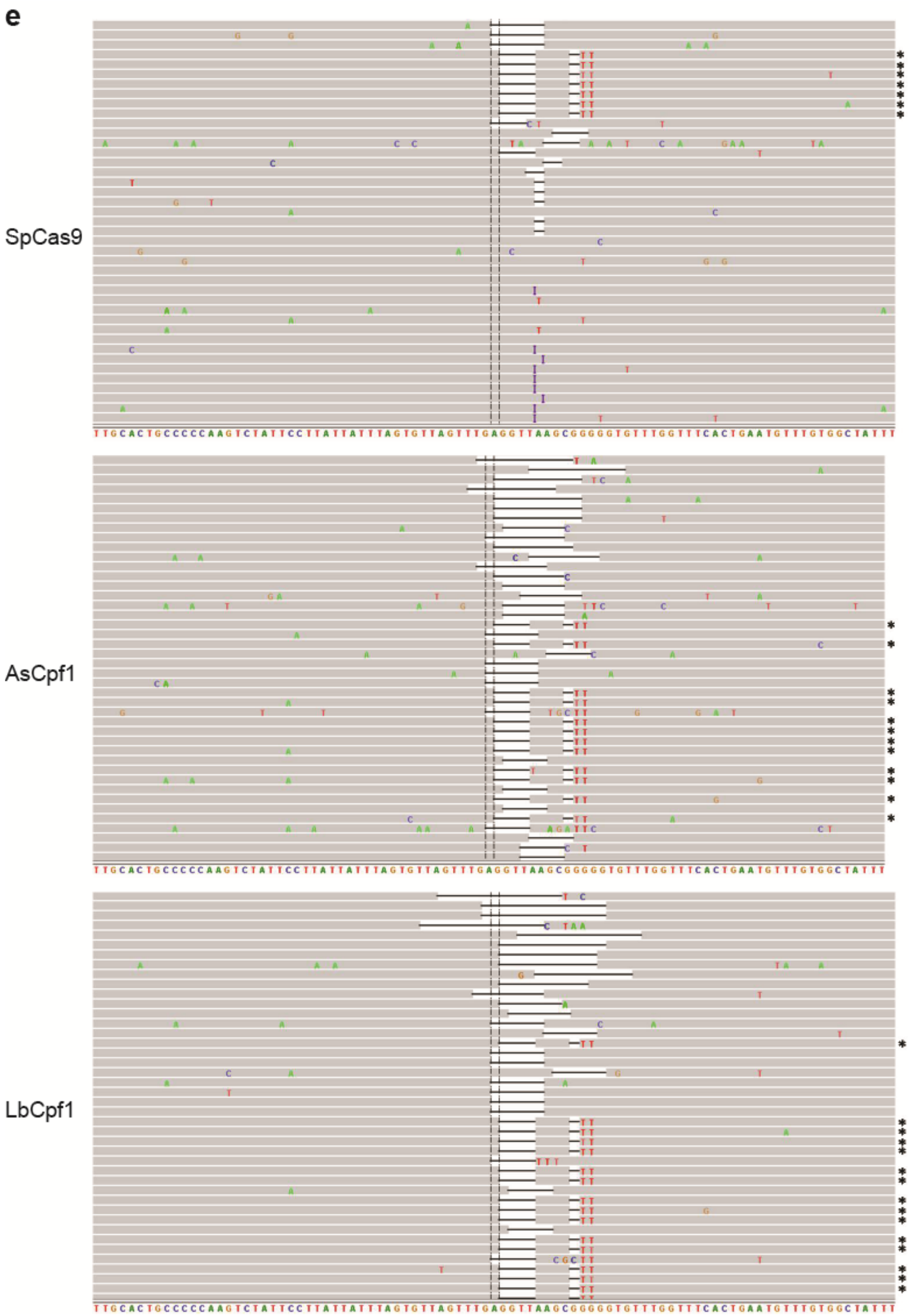
e
SpCas9
AsCpf1
LbCpf1

f

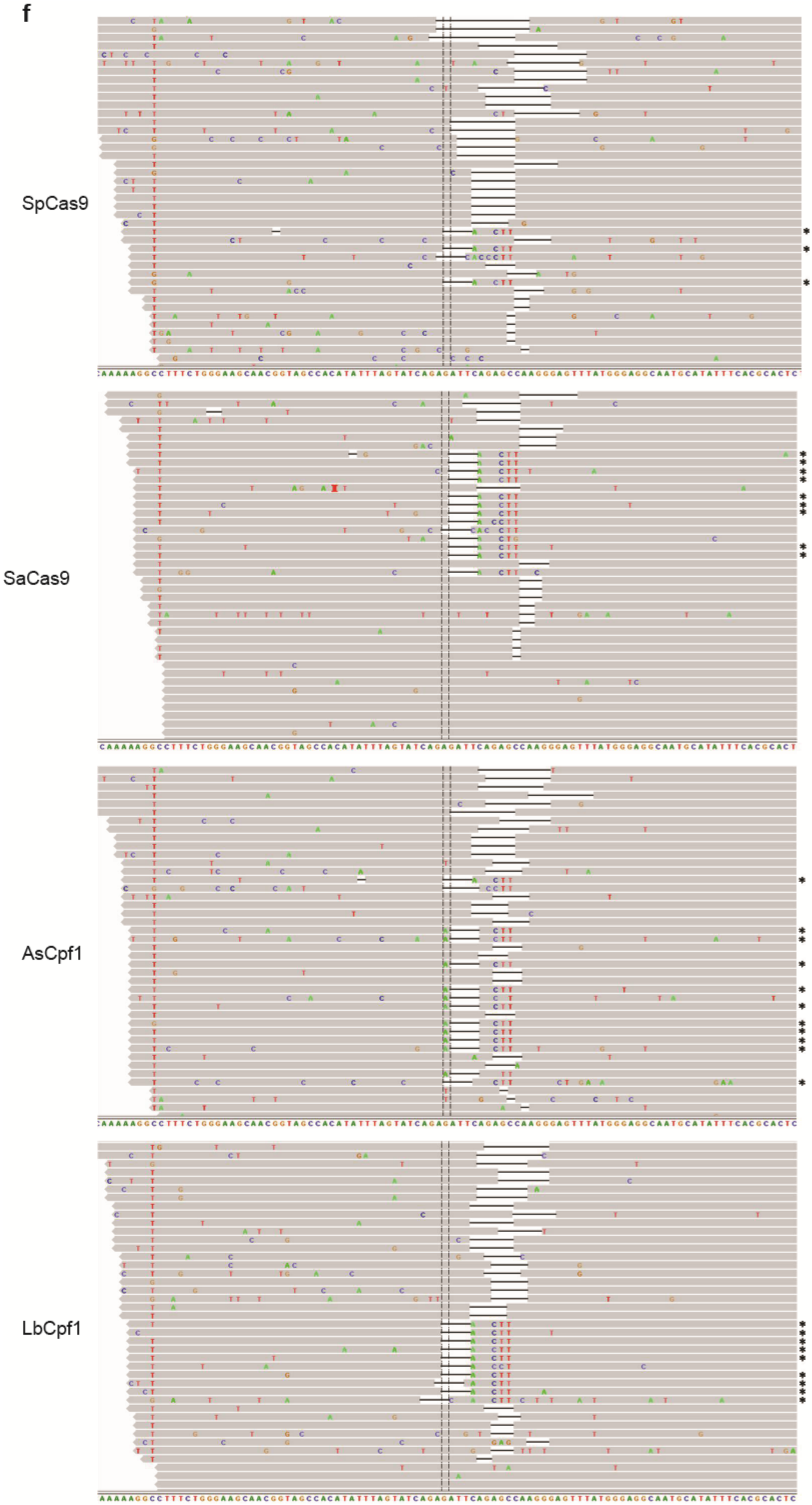

g

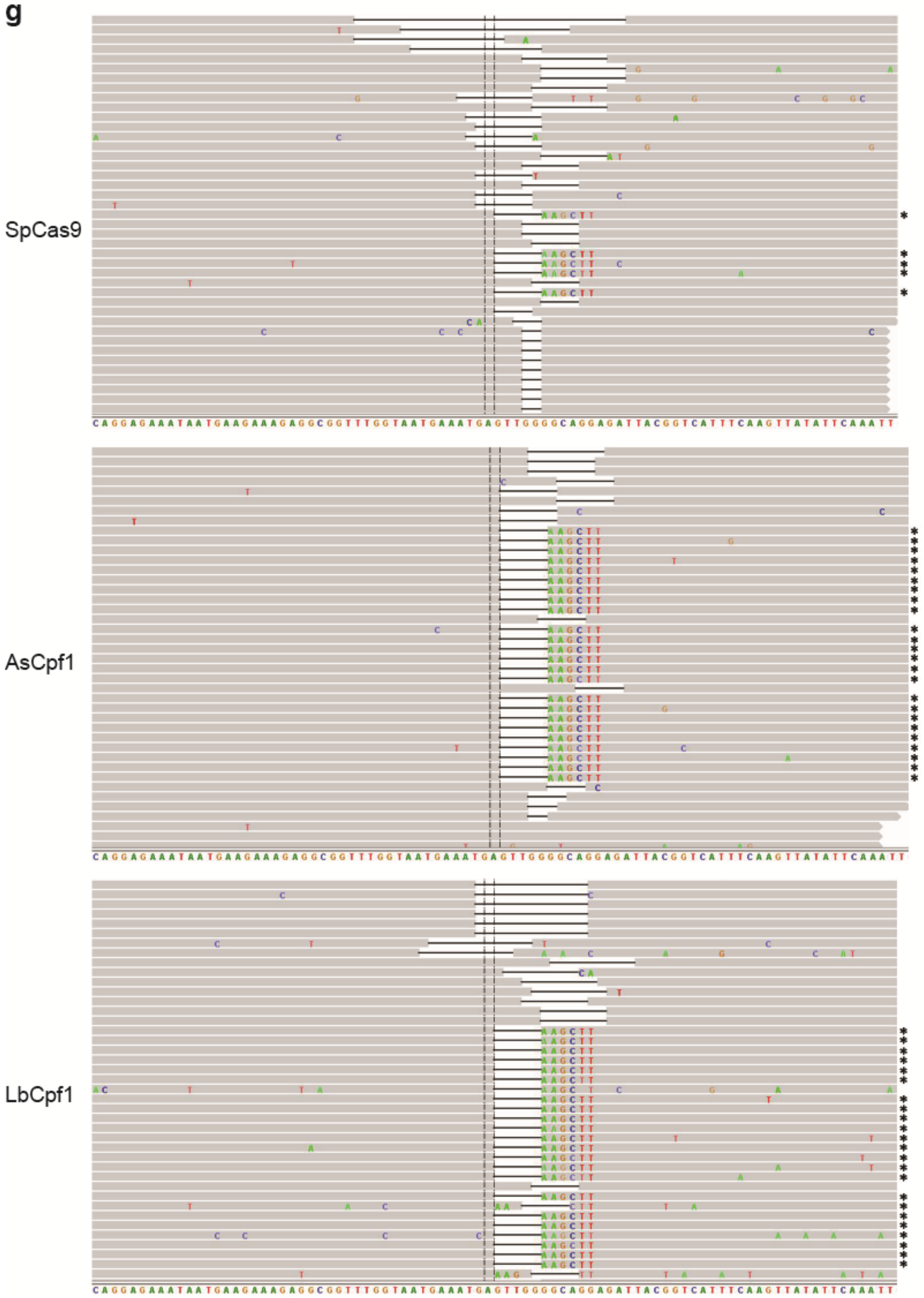
SpCas9
AsCpf1
LbCpf1
CAGGAGAAATAATGAAGAAAGAGGCGGTTTGGTAATGAAATGAGTTGGGGCAGGAGATTACGGTCATTTCAAGTTATATTCAAATT

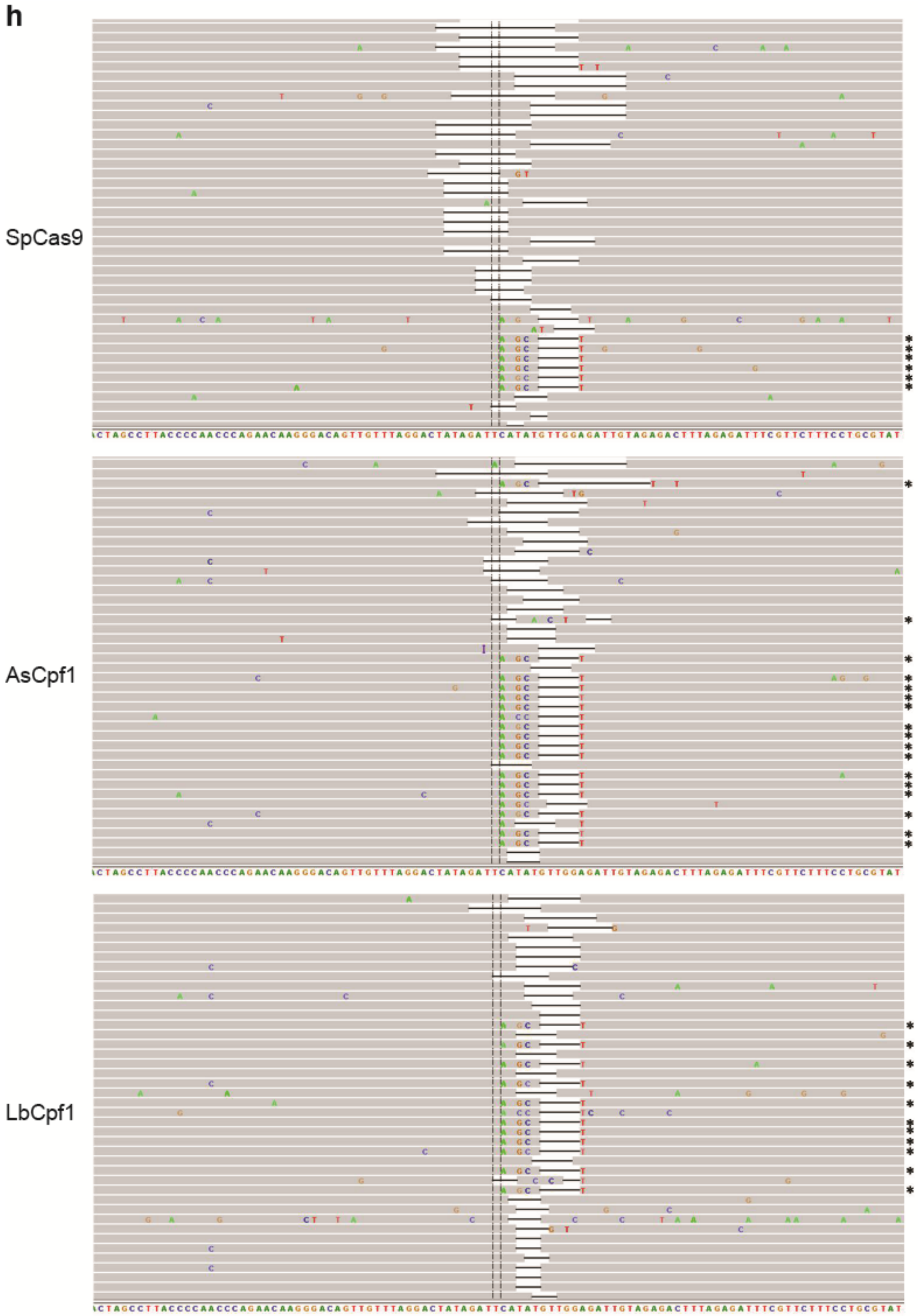
h
SpCas9
AsCpf1
LbCpf1

Figure S21 Illumina sequencing reads of targeted genomic loci.

Representative Integrative Genomics Viewer (IGV) screenshots of some of the sequencing reads covering target sites located in the **a** CACNA1D, **b** PPP1R12C, **c** ALK (A3), **d** ALK (B4), **e** EGFR (A11), **f** EGFR (A12), **g** EGFR (B8), and **h** STAG2 (B18) genes. Asterisks indicate the reads where the relevant restriction site was correctly incorporated into the targeted locus using ssODNs as donors. We could visually observe that SpCas9 was able to produce as many random indels as the Cpf1 nucleases, but smaller fractions of the reads for SpCas9 contained the desired genome modifications.

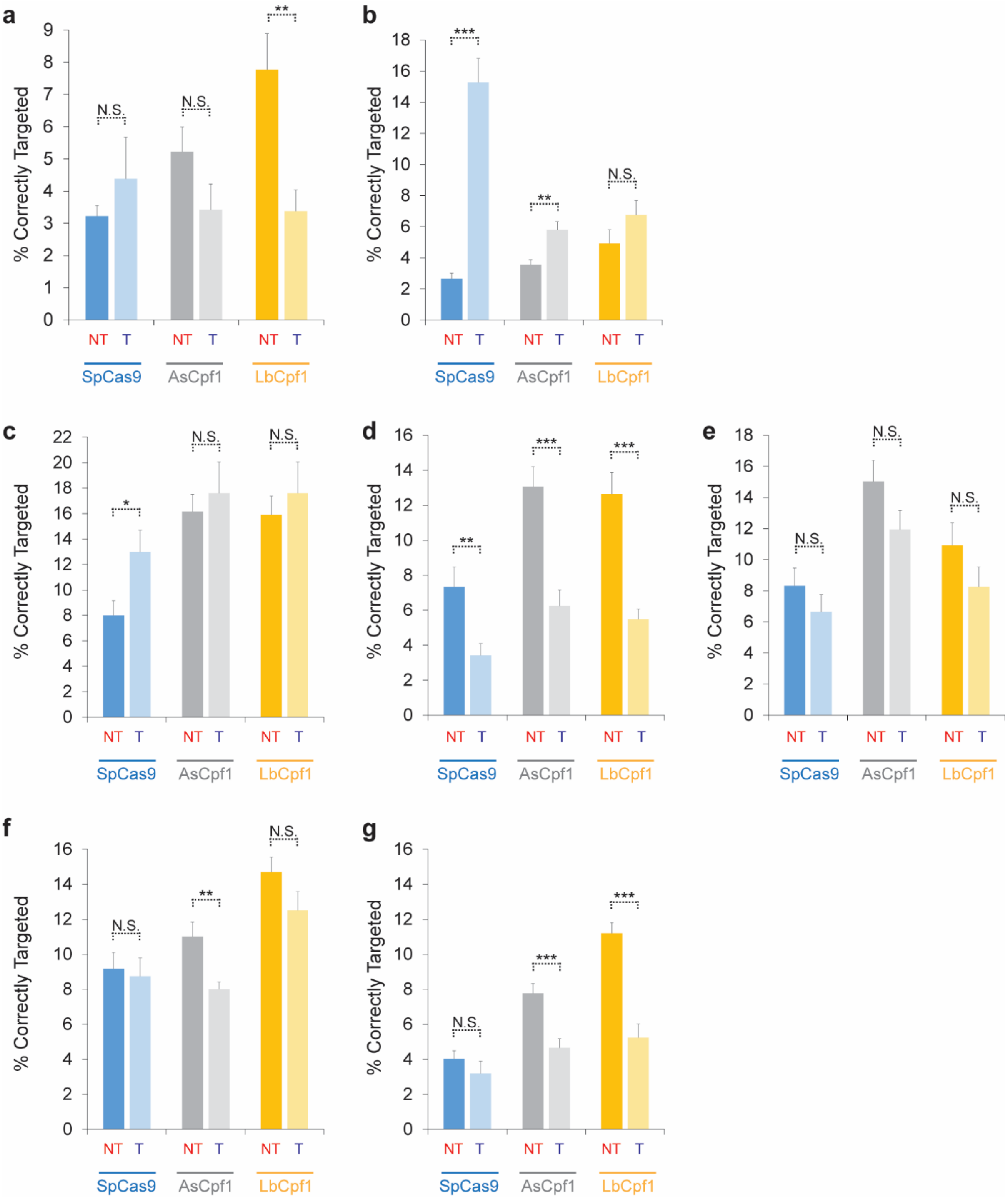
a
% Correctly Targeted
0 1 2 3 4 5 6 7 8 9
N.S.
N.S.
**
NT T NT T NT T
SpCas9 AsCpf1 LbCpf1
b
% Correctly Targeted
0 2 4 6 8 10 12 14 16 18
***
**
N.S.
NT T NT T NT T
SpCas9 AsCpf1 LbCpf1
c
% Correctly Targeted
0 2 4 6 8 10 12 14 16 18 20 22
*
N.S.
N.S.
NT T NT T NT T
SpCas9 AsCpf1 LbCpf1
d
% Correctly Targeted
0 2 4 6 8 10 12 14 16
**
***
***
NT T NT T NT T
SpCas9 AsCpf1 LbCpf1
e
% Correctly Targeted
0 2 4 6 8 10 12 14 16 18
N.S.
N.S.
N.S.
NT T NT T NT T
SpCas9 AsCpf1 LbCpf1
f
% Correctly Targeted
0 2 4 6 8 10 12 14 16
N.S.
**
N.S.
NT T NT T NT T
SpCas9 AsCpf1 LbCpf1
g
% Correctly Targeted
0 2 4 6 8 10 12 14 16
N.S.
***
***
NT T NT T NT T
SpCas9 AsCpf1 LbCpf1

Figure S22 Evaluation of symmetric ssODN donors with different orientations by Illumina deep sequencing.

We tested single-stranded DNA donors that are complementary to either the target strand (and hence are of the non-target strand sequence and are denoted NT) or the non-target strand (and hence are of the target strand sequence and are denoted T). Each ssODN donor contains 27nt or 17nt homology arms flanking a restriction site. Cells were harvested 72 hours after transfection for analysis. The edited genomic loci are **a** CACNA1D (27nt), **b** PPP1R12C (27nt), **c** A3 (27nt), **d** A11 (27nt), **e** B18 (27nt), **f** B8 (27nt), and **g** B8 (17nt), with the length of the homology arms indicated in parenthesis. Data represent mean $\pm$ s.e.m ($n \geq 3$). (* $P < 0.05$, ** $P < 0.01$, *** $P < 0.001$, N.S.: not significant; Student's t-test)

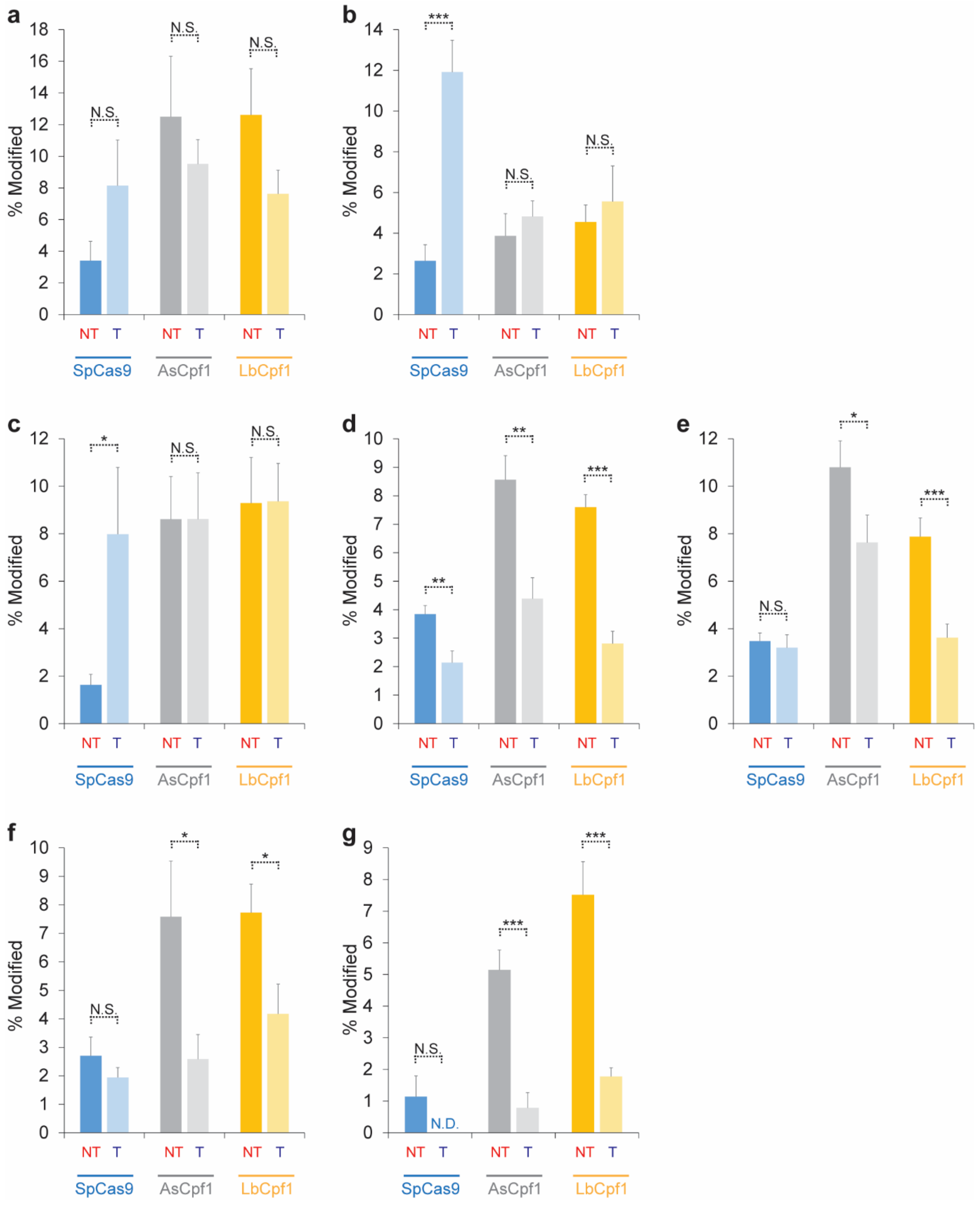

Figure S23 Evaluation of symmetric ssODN donors with different orientations by RFLP analysis. Details of the experiments are as given in Figure S22. Data represent mean ± s.e.m ($n \geq 2$). (* $P < 0.05$, ** $P < 0.01$, *** $P < 0.001$, N.S.: not significant; Student's t-test)

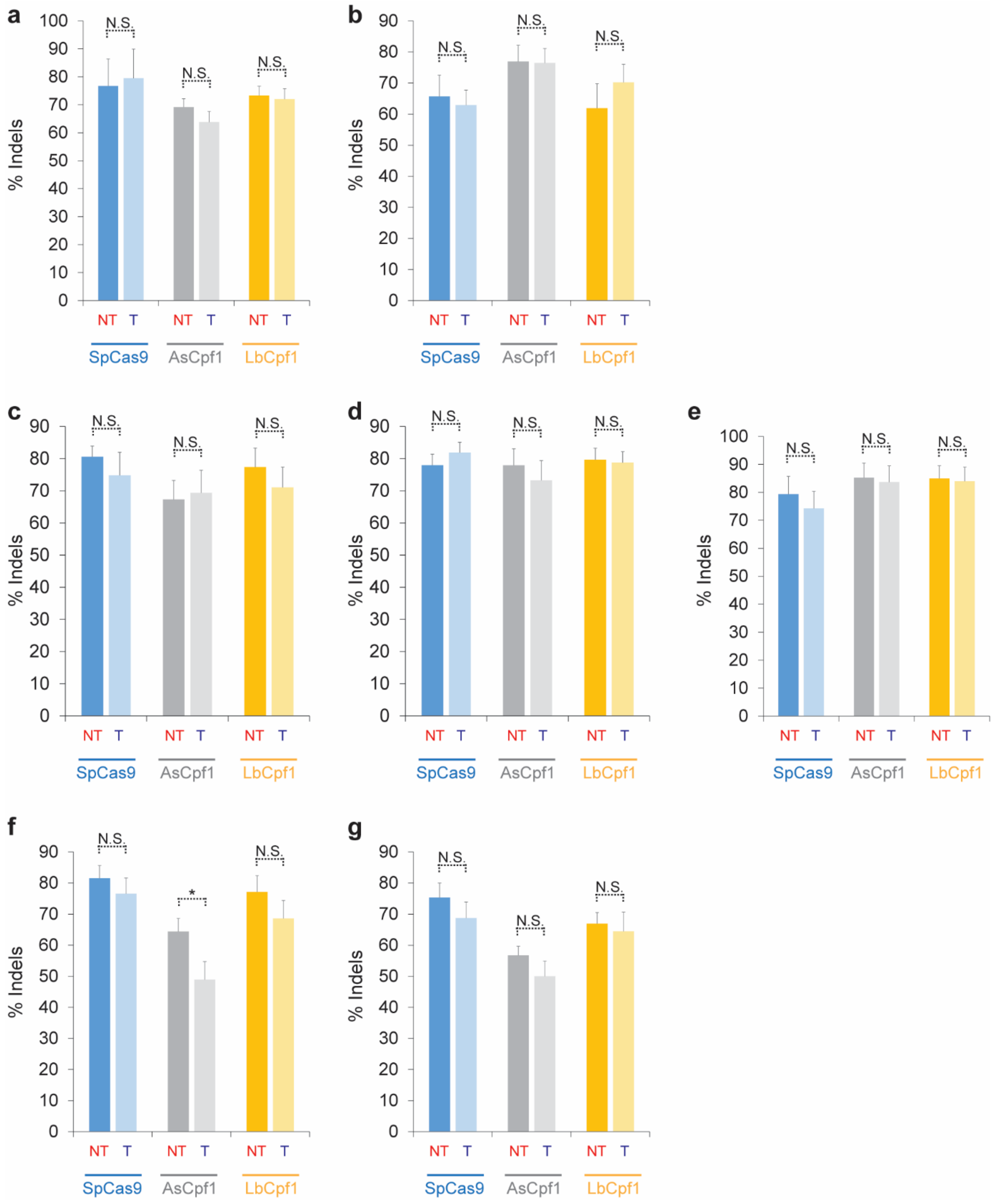

Figure S24 Measurements of cleavage rates in editing experiments with NT or T ssODNs. The extent of indel formation was quantified by Illumina deep sequencing at **a** CACNA1D (27nt), **b** PPP1R12C (27nt), **c** A3 (27nt), **d** A11 (27nt), **e** B18 (27nt), **f** B8 (27nt), and **g** B8 (17nt) loci, with the length of ssODN homology arms indicated in parenthesis. Data represent mean ± s.e.m ($n \geq 3$). (* $P < 0.05$, ** $P < 0.01$, *** $P < 0.001$, N.S.: not significant; Student's t-test)

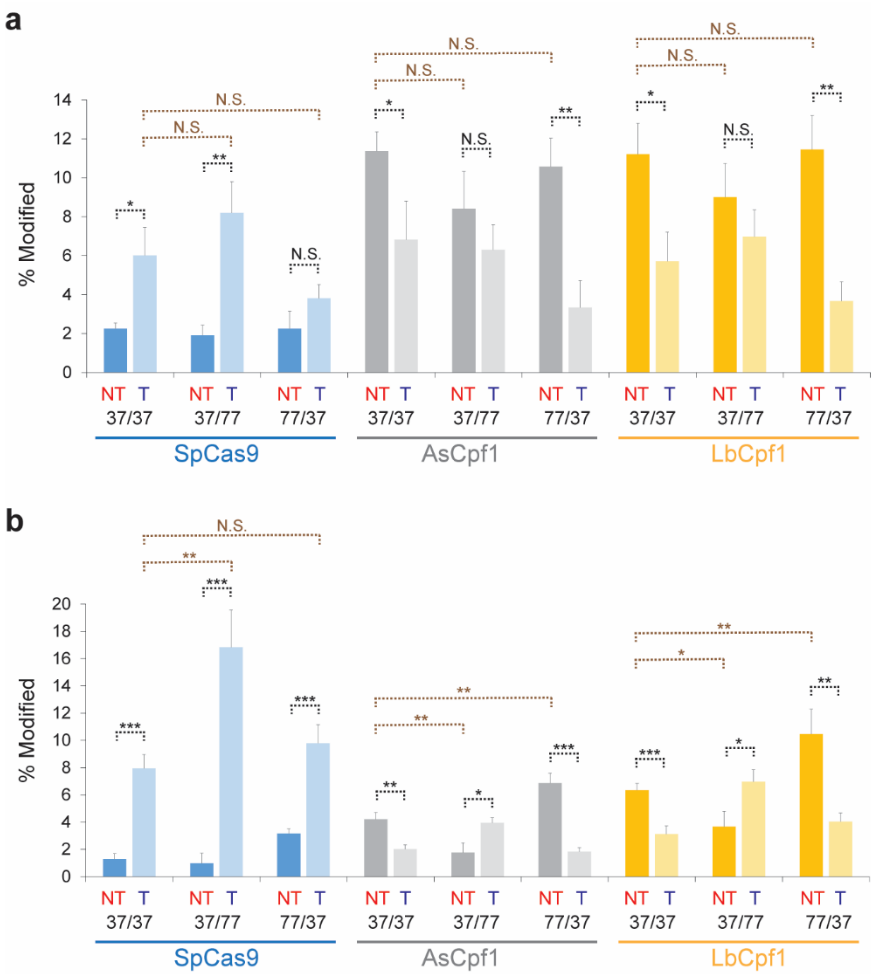

Figure S25 Evaluation of multiple symmetric and asymmetric ssODN donor designs used in combination with different CRISPR-Cas systems.

a, b Extent of precise gene editing by SpCas9, AsCpf1, and LbCpf1 with single-stranded DNA at the **a** CACNA1D or **b** PPP1R12C locus. Details of the various ssODN donors are given in Fig. 5a. Cells were harvested 72 hours after transfection and the gene targeting efficiencies were determined by RFLP analysis. Data represent mean ± s.e.m ($n \geq 5$). (* $P < 0.05$, ** $P < 0.01$, *** $P < 0.001$, N.S.: not significant; Student's t-test)

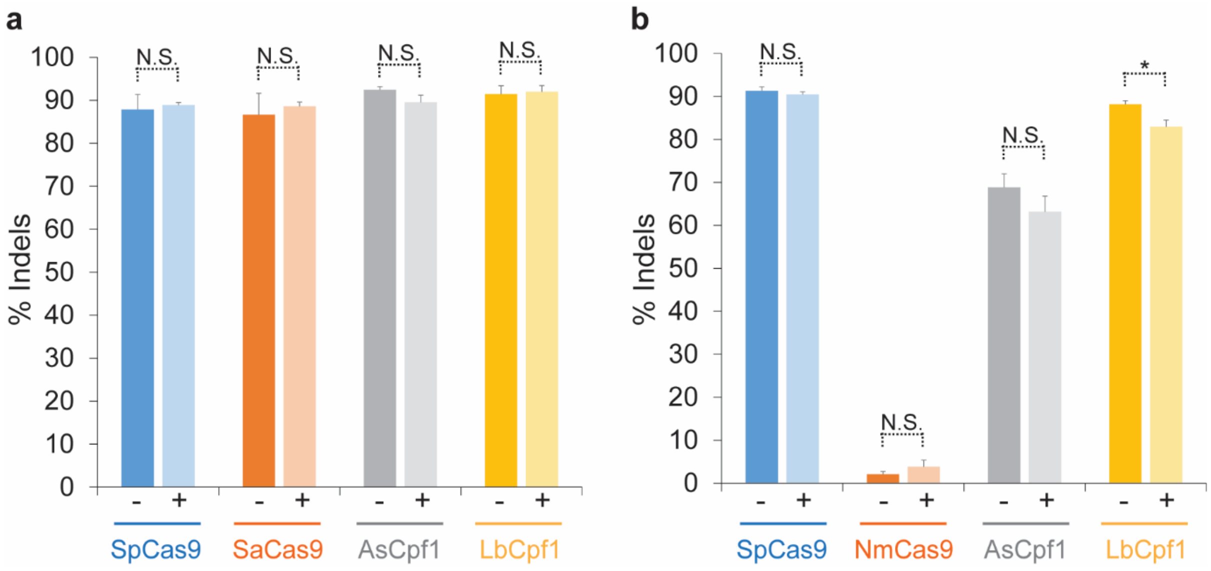

Figure S26 Measurements of cleavage rates in the absence or presence of ssODNs. Different CRISPR-Cas systems were transfected into cells without (-) or with (+) additional single-stranded DNA. Symmetric ssODNs with 47nt homology arms were used at the **a** A12 locus, while symmetric ssODNs with 27nt homology arms were used at the **b** B8 locus. The cells were harvested at 72 hours after transfection and the rate of indel formation was quantified by Illumina deep sequencing. Overall, we found that addition of single-stranded DNA of length 100nt or less did not increase the frequency of error-prone repair outcomes significantly. Data represent mean ± s.e.m ($n \geq 3$). (* $P < 0.05$, N.S.: not significant; Student's t-test)

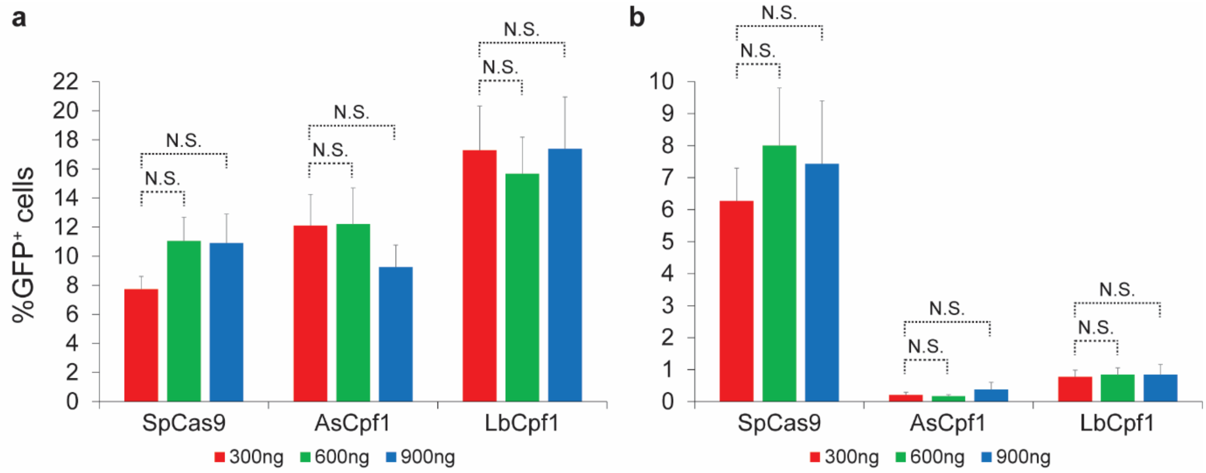

Figure S27 Extent of precise gene targeting with different amounts of plasmid donors. We sought to determine whether the amount of donor templates transfected into the cell may affect the rate of eGFP integration at the **a** CLTA or **b** GLUL genomic locus by SpCas9, AsCpf1, and LbCpf1. 300-900ng of linearized plasmid donors were introduced into the cell together with various CRISPR-Cas systems. The percentages of green fluorescent cells were quantified by flow cytometry. Overall, we observed similar HDR frequencies for all three quantities of plasmids tested. Data represent mean ± s.e.m (n = 4). (N.S.: not significant; Student's t-test)

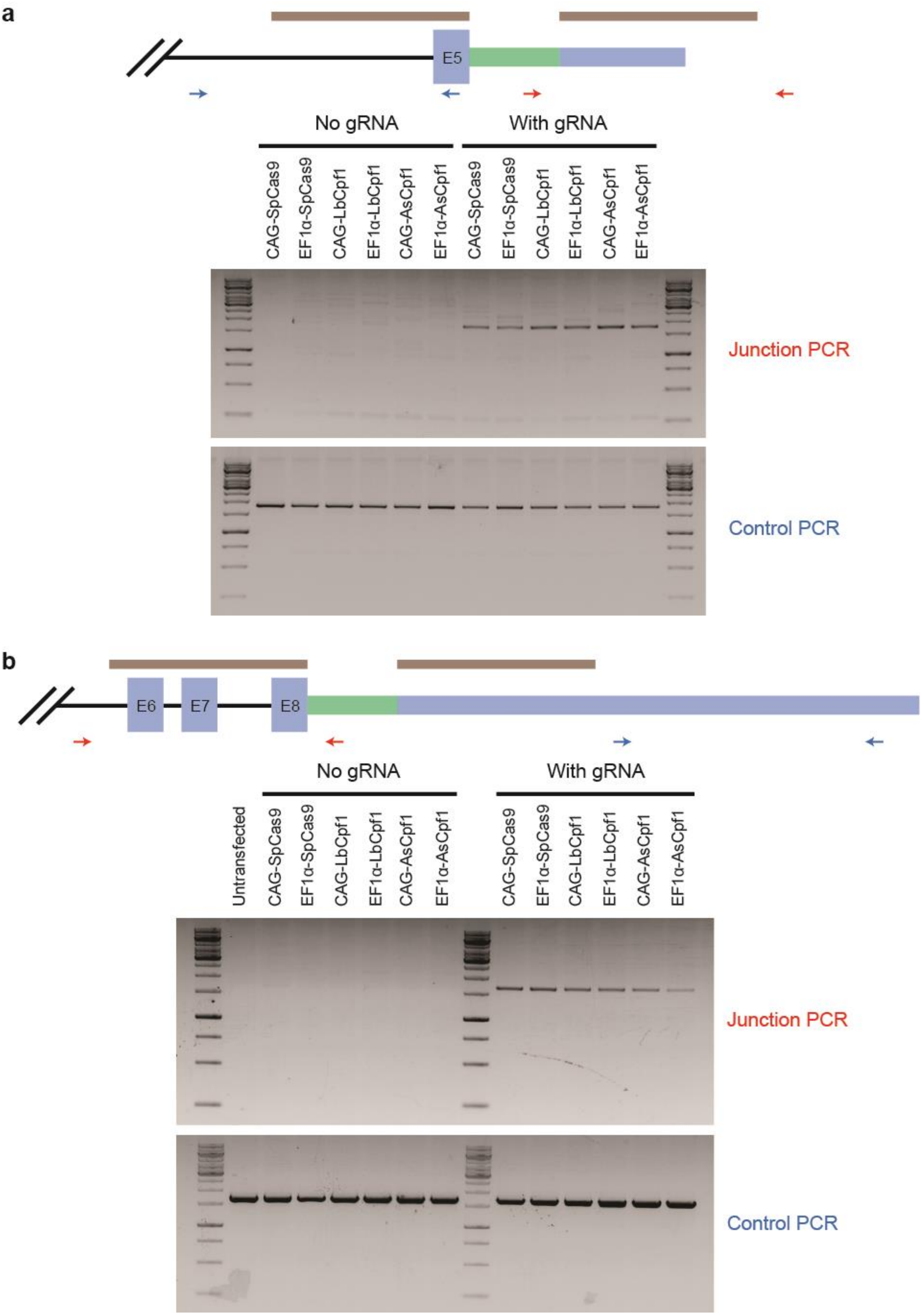
a
E5
No gRNA
With gRNA
CAG-SpCas9
EF1α-SpCas9
CAG-LbCpf1
EF1α-LbCpf1
CAG-AsCpf1
EF1α-AsCpf1
CAG-SpCas9
EF1α-SpCas9
CAG-LbCpf1
EF1α-LbCpf1
CAG-AsCpf1
EF1α-AsCpf1
Junction PCR
Control PCR
b
E6
E7
E8
No gRNA
With gRNA
Untransfected
CAG-SpCas9
EF1α-SpCas9
CAG-LbCpf1
EF1α-LbCpf1
CAG-AsCpf1
EF1α-AsCpf1
CAG-SpCas9
EF1α-SpCas9
CAG-LbCpf1
EF1α-LbCpf1
CAG-AsCpf1
EF1α-AsCpf1
Junction PCR
Control PCR

Figure S28 Correct integration of eGFP at endogenous genes.

We performed PCR to check whether the P2A-eGFP cassette was correctly integrated at the C-terminal end of **a** CLTA and **b** GLUL. Red arrows indicate the primers for the junction PCR, while blue arrows indicate the primers for the control PCR. In the absence of a sgRNA, no band was observed in the junction PCR. However, in the presence of the relevant sgRNA, bands of the expected size were observed for all the Cas nucleases, indicating that the cassette was targeted to the right genomic loci.

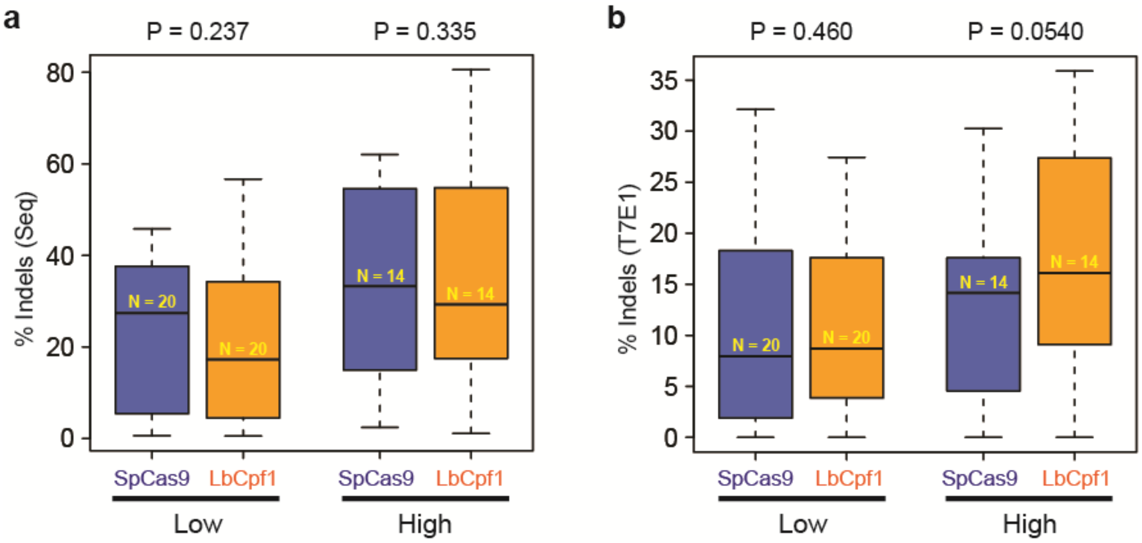

Figure S29 Pairwise comparison of SpCas9 and LbCpf1.

We compared the ability of SpCas9 and LbCpf1 to edit genes of different expression levels. Only sgRNAs of the optimal lengths for both SpCas9 and LbCpf1 (19-22nt inclusive) were considered. Overall, from **a** deep sequencing analysis and **b** T7E1 assays, we found that the editing activities of SpCas9 and LbCpf1 were not significantly different in both lowly expressed and highly expressed genes ($P > 0.05$, Wilcoxon rank sum test).

Table S1 Sites targeted for the initial testing of our CRISPR constructs.

Enzyme	sgRNA	Genomic locus (hg38)	Sequence
SpCas9	EMX1 Site 1	chr2:72,933,853-72,933,875	GAGTCCGAGCAGAAGAAGAAggg
	FANCF Site 1	chr11:22,625,786-22,625,808	GGAATCCCTTCTGCAGCACCtgg
	TAT	chr16:71,575,893-71,575,915	TCCTCCTGAGACTCCATACCtgg
	WAS Site 1	chrX:48,686,151-48,686,173	CCCATCCATCCAGAGACACAggg
SaCas9	EMX1 Site 2	chr2:72,933,960-72,933,986	GCAACCACAAACCCACGAGGGcagagt
	EMX1 Site 3	chr2:72,933,828-72,933,856	GCAGAAGCTGGAGGAGGAAGGGCctgagt
	FANCF Site 2	chr11:22,625,705-22,625,731	GCAAGGCCCGGCGCACGGTGGcggggt
	FANCF Site 3	chr11:22,625,804-22,625,830	GTAGGGCCTTCGCGCACCTCAtggaat
NmCas9	FANCF Site 4	chr11:22,625,776-22,625,806	GAAAAGCGATCCAGGTGCTGCAGaagggatt
	WAS Site 2	chrX:48,686,209-48,686,237	GCGTAAGTGGGTGAATGGATAggtagatt
AsCpf1/ LbCpf1	DNMT1 Site 1	chr19:10,133,942-10,133,968	tttcCCTCACTCCTGCTCGGTGAATTT
	DNMT1 Site 2	chr19:10,133,850-10,133,876	tttgAGGAGTGTTCAGTCTCCGTGAAC
	DNMT1 Site 3	chr19:10,133,767-10,133,793	tttcCTGATGGTCCATGTCTGTTACTC
	DNMT1 Site 4	chr19:10,133,690-10,133,716	tttaTTTCCCTTCAGCTAAAATAAAGG
	EMX1 Site 4	chr2:72,933,754-72,933,780	tttcTCATCTGTGCCCCTCCCTCCCTG
	EMX1 Site 5	chr2:72,933,792-72,933,818	tttgTCCTCCGGTTCTGGAACCACACC

Table S2 PCR primers used in T7E1 assays for initial validation of constructs.

**Primer Name**	**Primer Sequence**
EMX1_T7E1_Set1_FOR	GCC CCT AAC CCT ATG TAG CC
EMX1_T7E1_Set1_REV	GGA GAT TGG AGA CAC GGA GA
EMX1_T7E1_Set2_FOR	CTG TGT CCT CTT CCT GCC CT
EMX1_T7E1_Set2_REV	CTC TCC GAG GAG AAG GCC AA
FANCF_T7E1_FOR	ACC TCT TTG TGT GGC GAA AG
FANCF_T7E1_REV	CCA GGC TCT CTT GGA GTG TC
TAT_T7E1_FOR	GAC AAC ATG AAG GTG AAA CCA A
TAT_T7E1_REV	GTC AAA GAA AGC CAG GAA AGA A
WAS_T7E1_Set1_FOR	CAG CCA ATG AAG GTG AGT CC
WAS_T7E1_Set1_REV	GTG GAT CCC ACA AAC CAT TC
WAS_T7E1_Set2_FOR	AGG AAT CAG AGG CAA AGT GG
WAS_T7E1_Set2_REV	TCC CAT CAA TTC ATC CCT CT
DNMT1_T7E1_FOR	ACT CAG GCG GGT CAC CTA C
DNMT1_T7E1_REV	AAG CGA ACC TCA CAC AAC AG

Table S3 Perfectly matched sites for evaluating various CRISPR-Cas systems.

**Enzyme**	**sgRNA**	**Genomic locus (hg38)**	**Sequence**
SpCas9/ SaCas9/ AsCpf1/ LbCpf1	ALK Site A1	chr2:29,729,659-29,729,685	tttaGAGCCAGGCGATTTGGAgggggt
	ALK Site A2	chr2:29,663,048-29,663,075	tttaGGAAGTGTGTTCAGTGGAaggagt
	ALK Site A3	chr2:29,222,693-29,222,721	tttaGTGGACAAACACGAGAGGCgggggt
	ALK Site A4	chr2:29,533,045-29,533,074	ttttGAGTAATGCTTTGCCTTGGTaggaat
	ALK Site A5	chr2:29,590,027-29,590,057	tttgGCTGTGGGTGGGCAGTGTCCCaggggt
	ALK Site A6	chr2:29,518,143-29,518,174	tttaGATGCGTTATTTAAAATGGGCCtggagt
	ALK Site A7	chr2:29,369,614-29,369,646	ttttGTGAAGGGAAGAAACCTGGGCCGtggaat
	ALK CDS 19nt	chr2:29,320,777-29,320,805	tttaGGGTCCTGACCTGCCATTGaggagt
	EGFR Site A8	chr7:55,170,010-55,170,036	tttgGTGTCTGTAGGAGGTAGtggggt
	EGFR Site A9	chr7:55,096,225-55,096,252	ttttGAAGAGAAAAGGCTTAGGtggggt
	EGFR Site A10	chr7:55,093,727-55,093,755	ttttGTTTTGTCTTAGTTTTGCAaggagt
	EGFR Site A11	chr7:55,126,943-55,126,972	tttaGTGTTAGTTTGAGGTTAAGCgggggt
	EGFR Site A12	chr7:55,158,474-55,158,504	tttaGTATCAGAGATTCAGAGCCAAgggagt
	EGFR Site A13	chr7:55,149,505-55,149,536	ttttGCTTTTAAACACCTGGGTCAGCtggggt
	EGFR Site A14	chr7:55,020,227-55,020,259	tttcGGCGCGGGCGCCCAGGAGGGAGCtggggt
	NF1 Site A15	chr17:31,270,967-31,270,993	tttaGTAATAAATGAAACACTtggagt
	NF1 Site A16	chr17:31,107,276-31,107,303	tttgGCCTCTGATAAGTGAATCtggagt
	NF1 Site A17	chr17:31,276,468-31,276,496	tttgGGGTTTCAGATTACAGACAagggat
	NF1 Site A18	chr17:31,317,858-31,317,887	tttaGCTCTCTCTATCTATGCATTaggaat
	NF1 Site A19	chr17:31,216,707-31,216,737	ttttGCACGACCCTGTAGATCTATTtggagt
	NF1 Site A20	chr17:31,209,121-31,209,152	tttgGTTTTCATGACTTGGAGGGCTGgggggt
	NF1 Site A21	chr17:31,233,504-31,233,536	tttaGTTAGTTGTTGCAATGCTGGAATtggaat
	KDM6A Site A22	chrX:44,952,570-44,952,596	ttttGTCAAAAGAGTCAGCAGaggggt
	KDM6A Site A23	chrX:45,104,627-45,104,654	tttaGAGAGAGAGAGGCTCTTAtggagt

	KDM6A Site A24	chrX:45,008,430-45,008,458	tttaGGCAACTGCCTGGACAGGGtggagt
	KDM6A Site A25	chrX:44,981,264-44,981,293	ttttGAATGACTGGCTATATAAATcggggt
	KDM6A Site A26	chrX:45,019,875-45,019,905	ttttGATCACCAATAGAACAAGTTCaggggt
	KDM6A Site A27	chrX:44,962,077-44,962,108	ttttGATGATAGTAAACATTTTGATGtggagt
	KDM6A Site A28	chrX:44,874,138-44,874,170	tttcGCAAGGGAGCAAGCAGGGTACCCgggggt
	STAG2 Site A29	chrX:124,092,832-124,092,858	ttttGTAGAGGCAACCGTGAAgggaat
	STAG2 Site A30	chrX:124,093,026-124,093,053	ttttGGCAACTTTCTTGACTGTgggggt
	STAG2 Site A31	chrX:123,995,891-123,995,919	tttgGTTGATGTTTTGAGTAACAagggat
	STAG2 Site A32	chrX:123,989,177-123,989,206	tttaGAGCATACTACTTCAAAAGAaggaat
	STAG2 Site A33	chrX:124,017,589-124,017,619	ttttGTGTGTGCTTTTATTTTGTTGgggggt
	STAG2 Site A34	chrX:124,076,081-124,076,112	tttaGCCTGACTTCAGTAGGTGAACAtggaat
	STAG2 Site A35	chrX:123,962,988-123,963,020	tttaGTCCTAGTGGTTAGTGTCGAAAGaggaat
	APC CDS 17nt	chr5:112,842,765-112,842,791	tttgGAGGCAGACTCACTTCTtggaat
	APC CDS 23nt	chr5:112,843,762-112,843,794	tttgGTCAGGGGCATCCACCTGAATAAaggagt
	ATM CDS 17nt	chr11:108,333,897-108,333,923	tttaGTAATTGGCTGGTCTGCtggaat
	ATM CDS 23nt	chr11:108,284,371-108,284,403	ttttGCCTATGAGGGTACCAGAGACAGtgggat
	KDM5C CDS 18nt	chrX:53,211,855-53,211,882	tttgGCTTTGAGCAGGCTACCCgggaat
SpCas9/ NmCas9/ AsCpf1/ LbCpf1	ALK Site B1	chr2:29,503,974-29,504,002	ttttGCACCAACCTAATAGTGaggtgatt
	ALK Site B2	chr2:29,852,735-29,852,763	tttgGAAGTGAGACCTTTGGGaggtgatt
	ALK Site B3	chr2:29,323,732-29,323,762	ttttGCTCAGCAATTGGCCTTAGtggtgatt
	ALK Site B4	chr2:29,770,055-29,770,086	tttgGTCTCTTGCTGGATATGGGAaggggatt
	ALK Site B5	chr2:29,851,771-29,851,803	tttgGTAACAACAGAGTCGTAGAAAtggggatt
	ALK Site B6	chr2:29,881,738-29,881,772	ttttGACTTGGAAAAGATGAAGAGGGTgggggatt
	EGFR Site B7	chr7:55,113,736-55,113,765	tttaGCTTTGACAGAGATTCCAaggtgatt

	EGFR Site B8	chr7:55,049,319-55,049,350	tttgGTAATGAAATGAGTTGGGGCaggagatt
	EGFR Site B9	chr7:55,188,477-55,188,510	ttttGAAATACAGCACTGAGGTTAAAaggggatt
	EGFR Site B10	chr7:55,045,993-55,046,027	tttgGGACATATTGAGTTAAATAAAACaggagatt
	NF1 Site B11	chr17:31,195,474-31,195,502	ttttGGAAGATGAACAGGTTTtggagatt
	NF1 Site B12	chr17:31,312,026-31,312,058	tttaGTAAGTTTACTGAAAATTTTGtggtgatt
	NF1 Site B13	chr17:31,360,186-31,360,220	tttgGAAAATTGGCTAGACATCATTCCtggtgatt
	NF1 Site B14	chr17:31,235,310-31,235,344	ttttGTGATGTAATTTGAGCTGAATCTtggagatt
	KDM6A Site B15	chrX:45,021,480-45,021,510	tttaGAATCAAGATAGTCTGCAAtggagatt
	KDM6A Site B16	chrX:44,970,630-44,970,662	ttttGTGCAGTCACCATATCAAACCtggagatt
	KDM6A Site B17	chrX:44,966,452-44,966,485	ttttGGTTATGAAGGTAATGAATCCAaggagatt
	STAG2 Site B18	chrX:124,025,089-124,025,119	tttaGGACTATAGATTCATATGTtggagatt
	STAG2 Site B19	chrX:124,097,858-124,097,888	ttttGTTGGTGTTCTCATCCAAAtggggatt
	STAG2 Site B20	chrX:123,969,297-123,969,331	tttaGGTCATTTATGCTCTATTTAAAAtggagatt

Table S4 Target sites with overlapping seed regions for Cas9 and Cpf1.

Enzyme	sgRNA	Genomic locus (hg38)	Sequence
SpCas9/ SaCas9	CACNA1D Cas9 17nt	chr3:53,803,516-53,803,538	GGAGTATTTCAGTAGTGaggaat
	CACNA1D Cas9 20nt	chr3:53,803,513-53,803,538	GCAGGAGTATTTCAGTAGTGaggaat
	CACNA1D Cas9 23nt	chr3:53,803,510-53,803,538	GGAGCAGGAGTATTTCAGTAGTGaggaat
AsCpf1/ LbCpf1	CACNA1D Cpf1 17nt	chr3:53,803,522-53,803,542	tttcAGTAGTGAGGAATGCTA
	CACNA1D Cpf1 20nt	chr3:53,803,522-53,803,545	tttcAGTAGTGAGGAATGCTACGA
	CACNA1D Cpf1 23nt	chr3:53,803,522-53,803,548	tttcAGTAGTGAGGAATGCTACGAGGA
SpCas9/ NmCas9	PPP1R12C Cas9 17nt	chr19:55,094,706-55,094,730	GGGACGTTTGGCTTCGCtggggatt
	PPP1R12C Cas9 20nt	chr19:55,094,703-55,094,730	GTGGGGACGTTTGGCTTCGCtggggatt
	PPP1R12C Cas9 23nt	chr19:55,094,700-55,094,730	GCTGTGGGGACGTTTGGCTTCGCtggggatt
AsCpf1/ LbCpf1	PPP1R12C Cpf1 17nt	chr19:55,094,712-55,094,732	tttgGCTTCGCTGGGGATTCA
	PPP1R12C Cpf1 20nt	chr19:55,094,712-55,094,735	tttgGCTTCGCTGGGGATTCAGGC
	PPP1R12C Cpf1 23nt	chr19:55,094,712-55,094,738	tttgGCTTCGCTGGGGATTCAGGCTCC

Table S5 Longer perfectly matched sites for NmCas9, AsCpf1, and LbCpf1.

Enzyme	sgRNA	Genomic locus (hg38)	Sequence
NmCas9/ AsCpf1/ LbCpf1	WDR5 Site C1	chr9:134,138,757-134,138,792	tttcGCGTTAGTGTTACAATCAGCCTGTgcaagatt
	COPA Site C2	chr1:160,289,337-160,289,373	tttgGTTTTCCAAAATGCACACTGCGGGTtattgatt
	STAG2 Site C3	chrX:124,091,896-124,091,931	tttaGAGCCAAAGCTAATGAAGATTTTAgaaagatt
	STAG2 Site C4	chrX:124,097,817-124,097,852	tttgGGAAGCACAACAGTAGAAGACAGCatgagatt
	HDAC2 Site C5	chr6:113,968,517-113,968,552	ttttGTGGGAGGGAATTAAAATATTTTGcagagatt
	HDAC2 Site C6	chr6:113,948,106-113,948,141	ttttGCTTTGGCCTAGCCTTCAATCATAtgctgatt
	GLUL Site C7	chr1:182,382,829-182,382,865	tttaGGAAATAAAGAAATATAATGGCACAtagggatt
	GLUL Site C8	chr1:182,381,657-182,381,693	ttttGTTCATTGTAATCAATTCCATGACAaaatgatt
	PARK7 Site C9	chr1:7,981,033-7,981,068	tttaGAATAAGTCAGTTATAACTGTAATgtgtgatt

Table S6 Additional 21nt perfectly matched sites for SpCas9 and SaCas9.

Enzyme	sgRNA	Genomic locus (hg38)	Sequence
SpCas9/ SaCas9	HNF4A Site D1	chr20:44,428,351-44,428,377	GGGTGCAGGGGGTGGTGGGCAtggggt
	HNF4A Site D2	chr20:44,430,757-44,430,783	GCTGGAAGCTGGGAGGTCAGGtggggt
	ADARB2 Site D3	chr10:1,363,118-1,363,144	GGGCGCAGCAAGAAGCTGGCCcggggt
	ASCL2 Site D4	chr11:2,268,594-2,268,620	GGCCCCCGTGACAGCCCACCCtggggt
	KCNA1 Site D5	chr12:4,912,165-4,912,191	GGTGCCCAGCGTGATGAAATAaggaat
	KCNA1 Site D6	chr12:4,912,362-4,912,388	GCTCATCTTTTTCCTCTTCATcggggt
	WDR5 Site D7	chr9:134,158,548-134,158,574	GTAAAGAGCCGTTTGTGTCTTgggagt
	WDR5 Site D8	chr9:134,158,970-134,158,996	GAGTCCCACTGGCATCTGACCtggagt
	HDAC2 Site D9	chr6:113,953,296-113,953,322	GGAAAGTATTCCCCATATTTAtggaat
	HDAC2 Site D10	chr6:113,940,149-113,940,175	GTCTGCCCATTTTAAGACTGTaggagt
	GLUL Site D11	chr1:182,385,459-182,385,485	GCATCGTGTGTGTGAAGACTTtggagt
	GLUL Site D12	chr1:182,385,407-182,385,433	GCCTGCACCATTCCAGTTCCCaggaat
	SRSF1 Site D13	chr17:58,006,416-58,006,442	GGAACAGGCCGAGGCGGCGGCgggggt
	SRSF1 Site D14	chr17:58,005,450-58,005,476	GTGATCCTCTGCTTCTCCTTGgggagt
	SOD1 Site D15	chr21:31,659,695-31,659,721	GTCGTAGTCTCCTGCAGCGTCtggggt
	SOD1 Site D16	chr21:31,668,581-31,668,607	GGGGCCTCAGACTACATCCAAgggaat
	VIM Site D17	chr10:17,229,626-17,229,652	GCGCCTGCGGAGCAGCGTGCCcggggt
	VIM Site D18	chr10:17,233,612-17,233,638	GTCTTGACCTTGAACGCAAAGtggaat

Table S7 PCR primers used in T7E1 assays.

Primer Name	Primer Sequence
A1_ALK_T7E1_FOR	AAA TCT CAT GGG TGC AGA GG
A1_ALK_T7E1_REV	CCA CGG TAA AAA GGC CAT AA
A2_ALK_T7E1_FOR	TGG TTG CTC AGG AAG ATG AA
A2_ALK_T7E1_REV	ACA CGT GAA GGC ATT TTT CC
A3_ALK_T7E1_FOR	TCG TCC TGT TCA GAG CAC AC
A3_ALK_T7E1_REV	TGT GTC CCT GGC AAA TAT CA
A4_ALK_T7E1_FOR	ACA GAG GGT TCA CGT TCT CG
A4_ALK_T7E1_REV	GTT CCA GGC ATT CCT TCT GA
A5_ALK_T7E1_FOR	CCA TGC ATG ATT TGG GTA GA
A5_ALK_T7E1_REV	AGC ACT TTG GCA GAA AGG AA
A6_ALK_T7E1_FOR	CCC CAG CTT TCA CAT CAT CT
A6_ALK_T7E1_REV	GTG TGT GCA TGG TGT GTG AC
A7_ALK_T7E1_FOR	CCT GCC ATT CTT CCA CTG AT
A7_ALK_T7E1_REV	GTG TAG CCG ATC CAA CCA TT
B1_ALK_T7E1_FOR	AGC AGG GGC TGG ATT TAT TT
B1_ALK_T7E1_REV	CTC AGC CTA AAG CCC TGT GT
B2_ALK_T7E1_FOR	CTT TCA AAG GTG TGG GGA AG
B2_ALK_T7E1_REV	GCA GAT GGC TGT CTT CTG GT
B3_ALK_T7E1_FOR	AAC TGG CTA CAG CCC AAG AA
B3_ALK_T7E1_REV	GCC AGT GGA CAA TTG ATG TG
B4_ALK_T7E1_FOR	TTG TTG TTG GGA CGT GTC AT
B4_ALK_T7E1_REV	AGA TAC TGG GCA GCA AAT GG
B5_ALK_T7E1_FOR	GGG GCT TCG TTT CTT ATT CC
B5_ALK_T7E1_REV	TTT CTG TCC AGC TCC CAA GT
B6_ALK_T7E1_FOR	AAA GTC ACA CCC CAT TCT GC
B6_ALK_T7E1_REV	CTG TGT CTT CCA GGA TGC AA
CDS_ALK_19nt_T7E1_FOR	GAAAGCCCAAGGTGTGAAGA
CDS_ALK_19nt_T7E1_REV	TGAGCCTCTGCTTTGTCAGA
A8_EGFR_T7E1_FOR	CTA TGG TTG CCC AAA AGC AT
A8_EGFR_T7E1_REV	GCC TGG AGA AAG ATG GAC AA
A9_EGFR_T7E1_FOR	TTG GCT TCC TAG ATC CCT GA
A9_EGFR_T7E1_REV	GGC ACA CAC GTG CAG ATA AG
A10_EGFR_T7E1_FOR	TGG TGA CTG TGT GAG CGA AT
A10_EGFR_T7E1_REV	TGC TTT ACG AGG CCA ATT TC
A11_EGFR_T7E1_FOR	CCC TGC CAC TCA TCA AAA AT
A11_EGFR_T7E1_REV	GGA GAG AAA TGC TCC TGC AC
A12_EGFR_T7E1_FOR	CAG TGA GTC ACA CCC TGG AA
A12_EGFR_T7E1_REV	GTG GAG GAG GAG ATG GGA AT
A13_EGFR_T7E1_FOR	TAA AAA CCT GGC CCA GAA CA
A13_EGFR_T7E1_REV	ATA GCA TGG CTG CTG CAT AA
A14_EGFR_T7E1_FOR	TGA GCC CCA TTT TGA AAC AC
A14_EGFR_T7E1_REV	AGG GAA GCT GAG GAA GGA AC

B7_EGFR_T7E1_FOR	TTT TGG TTC CTC CAT CTT TGA
B7_EGFR_T7E1_REV	TCC AGA GTG CCC ATG TCT TAC
B8_EGFR_T7E1_FOR	GGA GCA TGA AGC AGT CAT CA
B8_EGFR_T7E1_REV	CGA AGG ACT TCG ATT TTG CT
B9_EGFR_T7E1_FOR	CAG CTT TGG GAC AAG GAG AG
B9_EGFR_T7E1_REV	CGC CTC TCA CTC TGA ACT CC
B10_EGFR_T7E1_FOR	ATT GTT GGC TGT TCG GTG TT
B10_EGFR_T7E1_REV	ATG CCA AGC CTT CAG AGA AA
A15_NF1_T7E1_FOR	CGC TAA GAA AAG GTG CCA AT
A15_NF1_T7E1_REV	TGC ATC AGG AAA CTG TGC AT
A16_NF1_T7E1_FOR	TAT CGG TGC CTT CTA TTC CTG T
A16_NF1_T7E1_REV	TTC AGA GAC AGA ATC TCG GTT G
A17_NF1_T7E1_FOR	CTG TGC CCA GAA GGA AGA AG
A17_NF1_T7E1_REV	TCG GAG GGC TGG TAT CAT TA
A18_NF1_T7E1_FOR	CGT GGT CAC AGG TTT ATT CTG A
A18_NF1_T7E1_REV	AAG CAG GTG AGA CAG GGA GA
A19_NF1_T7E1_FOR	GGA AAA GTG TCC GAC TCA GG
A19_NF1_T7E1_REV	TTT GCT TTG CTG CCA TCT AGT
A20_NF1_T7E1_FOR	GGA TCA GTG ATG GCT GTA AGT G
A20_NF1_T7E1_REV	AAC TCA CTA CAG GTG GCA TGG
A21_NF1_T7E1_FOR	GCC AAT GTG GTT CCT TGT TC
A21_NF1_T7E1_REV	ACC CGT CTC AAC GTT TTA GC
B11_NF1_T7E1_Set1_FOR	TTC AAG GCA ACA TGA ATG GA
B11_NF1_T7E1_Set1_REV	TCA GGA AGG GAA TTC AGT GG
B11_NF1_T7E1_Set2_FOR	AAA TCC TGT CTT TCA AGG CAA C
B11_NF1_T7E1_Set2_REV	TTG AGC TCC CTT ATG GAT CTG T
B12_NF1_T7E1_FOR	GAC ACC ACG ATG TTG GGT AA
B12_NF1_T7E1_REV	TAC AGG CAG GAA CCA CTG C
B13_NF1_T7E1_FOR	CAA CAT GCA AAG GTT GTC ACT T
B13_NF1_T7E1_REV	GTG TCA ATC AAG GCA TCA AGA A
B14_NF1_T7E1_FOR	TGA ATG CCC AAT TCC TTT TC
B14_NF1_T7E1_REV	GCA TGG AGT CTG CCA ATT CT
A22_KDM6A_T7E1_FOR	TTA ACA CAC ACA AAA AGG ATG GA
A22_KDM6A_T7E1_REV	TTT GAG ACG TAG TTT TGC TGT CA
A23_KDM6A_T7E1_FOR	CCT GCA AAT AAC AAG GGG TCT
A23_KDM6A_T7E1_REV	GTC TGG GGC TAA TCA AAG CA
A24_KDM6A_T7E1_FOR	CTT CCC CTG CTC TCA AGA AG
A24_KDM6A_T7E1_REV	TAT AGG GTG GTG GGG ATT GA
A25_KDM6A_T7E1_FOR	CCC TCT TCC CCC TAC TTC AT
A25_KDM6A_T7E1_REV	ATA GCT GCA CAA GCG GAA GT
A26_KDM6A_T7E1_FOR	GAG ACG GTC TTC ATA TTT TCC AA
A26_KDM6A_T7E1_REV	TGT GGC TAT GAT GTT TCG AAC T
A27_KDM6A_T7E1_FOR	GAT GAG CCT TGG ATA AAA CCA G
A27_KDM6A_T7E1_REV	CAC AAT CTG AAA ATC CAT GAG G

A28_KDM6A_T7E1_FOR	CCG ACC TAA ACT CCG TGA AA
A28_KDM6A_T7E1_REV	CCT CTT TGG GTT CGT GAG AT
B15_KDM6A_T7E1_FOR	ACT ACC TGC ACC CTG CAC TT
B15_KDM6A_T7E1_REV	GTC TTG CTG GGC TTT TTC TG
B16_KDM6A_T7E1_FOR	GCA CAG AAG AAA CAG ACT GAA AAG
B16_KDM6A_T7E1_REV	ACA TCT TTT CAC ATC ACA TGG ACT
B17_KDM6A_T7E1_FOR	GGA GTG CAG TGG CAG AAA C
B17_KDM6A_T7E1_REV	TGC CCA GGT CAT CTA CAC AA
A29_STAG2_T7E1_FOR	AAG ACA CCC CCA CAG TCA AG
A29_STAG2_T7E1_REV	CTC CCG GCA ACT ACA AGA AA
A30_STAG2_T7E1_FOR	TTC ACG GTT GCC TCT ACA AA
A30_STAG2_T7E1_REV	TGC CTC AGG AAG AAA TGA GG
A31_STAG2_T7E1_FOR	TGC AAG CAG CAT TTA TGA AAA
A31_STAG2_T7E1_REV	AAA CAT GAT TGA AGG CAT TGG
A32_STAG2_T7E1_FOR	TGT CTC TCC CAC CAC CTT CT
A32_STAG2_T7E1_REV	TTC TAA AAG CCA TGG GGA TG
A33_STAG2_T7E1_FOR	CCA CTG TGC CCA GCT AAT TT
A33_STAG2_T7E1_REV	AAC AGA TGA TCG CGT TAT TTC TAA
A34_STAG2_T7E1_FOR	TTG GGG TTT GAG GTA AGG TG
A34_STAG2_T7E1_REV	GGC TTC ATC CTC TTG CTG AC
A35_STAG2_T7E1_FOR	TAA ATT GAG GGC CAT GGT GT
A35_STAG2_T7E1_REV	AAA AGT GCT GCG AGG ACA GT
B18_STAG2_T7E1_FOR	CAC ATT TAG GGT GGA AAA ACC T
B18_STAG2_T7E1_REV	GGC ATT GGA GGA ACT TTA TTA GG
B19_STAG2_T7E1_FOR	GCC CCT CAG TTG TAA CAT TCA
B19_STAG2_T7E1_REV	TCA GCA ACC GTG TGA GAA AG
B20_STAG2_T7E1_FOR	GAT AAG TTG CTG CCT GAG GTC T
B20_STAG2_T7E1_REV	ACT TGG GGC CCT GAT ATT AAC T
CDS_APC_17nt_T7E1_FOR	GAG TTT GTG CCT GGG ACC TA
CDS_APC_17nt_T7E1_REV	AGC CAA GCC ATC TGT GAA AT
CDS_APC_23nt_T7E1_FOR	AAG TGC TAT CTG CGC TGC TT
CDS_APC_23nt_T7E1_REV	CCA AGT ATC CGC AAA AGG AA
CDS_ATM_17nt_T7E1_FOR	GGC ATA AGC ACA CGG AAA CT
CDS_ATM_17nt_T7E1_REV	TCC TCA GGT GGA ATC TGG TC
CDS_ATM_23nt_T7E1_FOR	TTT GAT TCC ACA TCT GGT GAT T
CDS_ATM_23nt_T7E1_REV	GGA GAC AAC ACG ACA TAA CCA A
CDS_KDM5C_18nt_T7E1_FOR	AGC CGA AGT GGT AAG AGC AA
CDS_KDM5C_18nt_T7E1_REV	GCC AAA TTC TTT GGA ATG GA
CACNA1D_T7E1_FOR	ACA GAC ACA CAC ACG GTG CT
CACNA1D_T7E1_REV	TGG AGT TTC TGC TCC CAT TT
PPP1R12C_T7E1_FOR	AAG TTC TGT GGG AGG GGA CT
PPP1R12C_T7E1_REV	TCT CAG TTC TCG CAC TGC TG
CLTA_T7E1_FOR	AGG CAG TTG CTT GTG TAG CA
CLTA_T7E1_REV	TTA GTT CAA GGC AGG GCT GT

GLUL_T7E1_FOR	GGC TCC ATA CCT GGA GAC AA
GLUL_T7E1_REV	CCT CTA TCC CAG CCA AAC AA
C1_WDR5_T7E1_FOR	GTA CTC GGC CCT AGA TGC AG
C1_WDR5_T7E1_REV	CTG CAG TTC AAT CGG TTT CA
C2_COPA_T7E1_FOR	CCA CAG CAG CTT TCT TTC CT
C2_COPA_T7E1_REV	GCA ATC CTC TGC CTC AGC
C3_STAG2_T7E1_FOR	ATT TAT GCA GGC CAC CAC TC
C3_STAG2_T7E1_REV	GGG ACC ACA TTC ATT GCC TA
C4_STAG2_T7E1_FOR	GCC CCT CAG TTG TAA CAT TCA
C4_STAG2_T7E1_REV	TCA GCA ACC GTG TGA GAA AG
C5_HDAC2_T7E1_FOR	GGT AGT GAT GGG CTC TGA GG
C5_HDAC2_T7E1_REV	TTC CCA ATC CAA TCC ATG TT
C6_HDAC2_T7E1_FOR	CCA ATT CCA TTA AGA CCA GCA
C6_HDAC2_T7E1_REV	GGT GGC TCT GTT CTC TGT CC
C7_GLUL_T7E1_FOR	ACT CAG GGG AGC AAA GGA AG
C7_GLUL_T7E1_REV	TCT GCT CTT GGA GGA GAT GG
C8_GLUL_T7E1_FOR	GGC GTG GTC CTA GTT TAT GC
C8_GLUL_T7E1_REV	CCC TAG ACA ACA CCC ATC CA
C9_PARK7_T7E1_FOR	TCA CTA TGT TGC CCA AGC AG
C9_PARK7_T7E1_REV	TGC CAT TTA GTG GCT GTC TG
D1_HNF4A_T7E1_FOR	ATG CAC CTT GTT CCT TTC AAC T
D1_HNF4A_T7E1_REV	CCA ACA ATG GCT TCA TTC AGT A
D2_HNF4A_T7E1_FOR	GCT GGT AGA GCA GGT GAG ATG
D2_HNF4A_T7E1_REV	AGT GCC TGG GAG TAA GGA AGA
D3_ADARB2_T7E1_FOR	CAA GAA GAA GGC CAA GAT GC
D3_ADARB2_T7E1_REV	GCA CCT GTT CTC CCA TCA AT
D4_ASCL2_T7E1_FOR	CTC CCC ACA GCT TCT CGA C
D4_ASCL2_T7E1_REV	GGC TGC ACT CCA GAT CTC A
D5_D6_KCNA1_T7E1_FOR	GTC TCC GTC ATG GTC ATC CT
D5_D6_KCNA1_T7E1_REV	AGG GCA ATT GTT AGC ACA CC
D7_WDR5_T7E1_FOR	TTG CTG GTG ACA TTT CTT GC
D7_WDR5_T7E1_REV	AGC CTC AGC ACC TCC TGT C
D8_WDR5_T7E1_FOR	TGT GAA TGG TTG TGG CAA GT
D8_WDR5_T7E1_REV	TAA CTG CTG TGC AGG TGA GC
D9_HDAC2_T7E1_FOR	GCT CAA AAA TGG GTT TCC TG
D9_HDAC2_T7E1_REV	GAG AAG GGC TTC ATG CTT TG
D10_HDAC2_T7E1_FOR	AAT TCT ACC ACC TTG CCC TCT
D10_HDAC2_T7E1_REV	AGA ATC AAT GTG GGC CTG AC
D11_D12_GLUL_T7E1_FOR	TGG GAG CAG ACA GAG CCT AT
D11_D12_GLUL_T7E1_REV	TTG CAA GTC ATC CTG CAA AG
D13_SRSF1_T7E1_FOR	CAA CTG AGC GAG CTT CTC CT
D13_SRSF1_T7E1_REV	GGT AAA TCA CCA CAG CAG CA
D14_SRSF1_T7E1_FOR	ATG TTT ACC GAG ATG GCA CTG
D14_SRSF1_T7E1_REV	TCA AAG ACA CGA AGG GAA TGT

D15_SOD1_T7E1_FOR	TTG CCA ATT TCG CGT ACT G
D15_SOD1_T7E1_REV	ACC CGC TCC TAG CAA AGG T
D16_SOD1_T7E1_FOR	TTG GGT ATT GTT GGG AGG A
D16_SOD1_T7E1_REV	TCA CAG GCT TGA ATG ACA AAG
D17_VIM_T7E1_FOR	CCT CCT ACC GCA GGA TGT T
D17_VIM_T7E1_REV	GGC TTT GTC GTT GGT TAG C
D18_VIM_T7E1_FOR	GCA TAA GCC ACC ATG ACC A
D18_VIM_T7E1_REV	TCT TGG CAG CCA CAC TTT C

Table S8 PCR primers used for constructing Illumina sequencing libraries.

Primer Name	Primer Sequence
A1_ALK_Adapter_FOR	GCG TTA TCG AGG TCT TCT GAA AGA TGC ACT CAA GAT GT
A1_ALK_Adapter_REV	GTG CTC TTC CGA TCT ATA AAA CAC CTT GGG GAA AAC A
A2_ALK_Adapter_FOR	GCG TTA TCG AGG TCT AGG CCC GTG GTT TAG TCT G
A2_ALK_Adapter_REV	GTG CTC TTC CGA TCT CCA GGC CTA GAA GAA TGT CC
A3_ALK_Adapter_FOR	GCG TTA TCG AGG TCC CCT GTG CAA AGG AGA AGA C
A3_ALK_Adapter_REV	GTG CTC TTC CGA TCT AGT CCC TGG TTG GAA TCC TT
A4_ALK_Adapter_FOR	GCG TTA TCG AGG TCC GGT CTT ATG CTT CAT CCT GA
A4_ALK_Adapter_REV	GTG CTC TTC CGA TCT AAC TAC TCC TGG CAC TTC CAA
A5_ALK_Adapter_FOR	GCG TTA TCG AGG TCT CTT AGT GTG CCT CTG GAA GAA
A5_ALK_Adapter_REV	GTG CTC TTC CGA TCT ATT CTA AAA GGG CTT TGG AGG T
A6_ALK_Adapter_FOR	GCG TTA TCG AGG TCG TTC CCC TAA TTG CCA GAG AC
A6_ALK_Adapter_REV	GTG CTC TTC CGA TCT AAT AAA ACA GGG TTG GTG GTG
A7_ALK_Adapter_FOR	GCG TTA TCG AGG TCC TTC TGT GTG CCT CTC CAC A
A7_ALK_Adapter_REV	GTG CTC TTC CGA TCT ACC ATT TCA GCC ACC TTG TC
B1_ALK_Adapter_FOR	GCG TTA TCG AGG TCT CCA TAA AGT TCT TGC CCT CA
B1_ALK_Adapter_REV	GTG CTC TTC CGA TCT CAC CTC AGG AAT TCC CAC TG
B2_ALK_Adapter_FOR	GCG TTA TCG AGG TCA TGG TAC ACA ATC TAA TGG GTA TGC
B2_ALK_Adapter_REV	GTG CTC TTC CGA TCT GAG AGA GAG AAA GCT CTG GTC TTT T
B3_ALK_Adapter_FOR	GCG TTA TCG AGG TCT TGA AGT CTT CCA GTG TGC TG
B3_ALK_Adapter_REV	GTG CTC TTC CGA TCT TAA ACT CAT CAG GGC AGC TTG
B4_ALK_Adapter_FOR	GCG TTA TCG AGG TCC TTG GTA GTT TAC CCT CCT CCT C
B4_ALK_Adapter_REV	GTG CTC TTC CGA TCT AGG ATT AGG CAT TGA TTG TGC TA
B5_ALK_Adapter_FOR	GCG TTA TCG AGG TCA TCT TGG CTG GTC CAT CTG TAA
B5_ALK_Adapter_REV	GTG CTC TTC CGA TCT AGG ACC ACT GTC TAG ACC AAG C
B6_ALK_Adapter_FOR	GCG TTA TCG AGG TCA GGT TTA AAA TGA GGG AAA ATG G

B6_ALK_Adapter_REV	GTG CTC TTC CGA TCT AAG GAA ATT TCA CAG GCA AGA AT
CDS_ALK_19nt_Adapter_FOR	GCG TTA TCG AGG TCG TGA AGT CCA GCC CTG AGT C
CDS_ALK_19nt_Adapter_REV	GTG CTC TTC CGA TCT GAT GGT GCC TCT CTG CTC TC
A8_EGFR_Adapter_FOR	GCG TTA TCG AGG TCG GGT GTT CCC ATT GTA TTG C
A8_EGFR_Adapter_REV	GTG CTC TTC CGA TCT TCT TAC CCC TGT GGT GCT TT
A9_EGFR_Adapter_FOR	GCG TTA TCG AGG TCG GGT ACA CAT CAC GGA ATT CTT
A9_EGFR_Adapter_REV	GTG CTC TTC CGA TCT TGA AGA TTC GAT TTG AGG TGA G
A10_EGFR_Adapter_FOR	GCG TTA TCG AGG TCA CAA GGA AGT CAT TTT TGA GGT G
A10_EGFR_Adapter_REV	GTG CTC TTC CGA TCT TAC CAG TGA CAG TTA CGA CAG GA
A11_EGFR_Adapter_FOR	GCG TTA TCG AGG TCA TTA GTT TTC ACT GGG GAA TGG
A11_EGFR_Adapter_REV	GTG CTC TTC CGA TCT TTG TCA GGG GAT GAG TGA GAT A
A12_EGFR_Adapter_FOR	GCG TTA TCG AGG TCG CCA TCC AAG AAA ATT GCT C
A12_EGFR_Adapter_REV	GTG CTC TTC CGA TCT TGC TGC CCC TAC TTG ACT CT
A13_EGFR_Adapter_FOR	GCG TTA TCG AGG TCC ACA CAC AAA TAT GGC CAA GA
A13_EGFR_Adapter_REV	GTG CTC TTC CGA TCT ATT GCA GGC ATA TTG CCA AG
A14_EGFR_Adapter_FOR	GCG TTA TCG AGG TCG AAG GGC TGC AGG TGG A
A14_EGFR_Adapter_REV	GTG CTC TTC CGA TCT GTT GGC AAA AGG CAG GTG
B7_EGFR_Adapter_FOR	GCG TTA TCG AGG TCC ACT ATT TCA TCC AAA AGC ATA GC
B7_EGFR_Adapter_REV	GTG CTC TTC CGA TCT AGG CAC CTA CTT TCT AGA GGT CTG
B8_EGFR_Adapter_FOR	GCG TTA TCG AGG TCG CCT AAC CCT TTC ACT GCT G
B8_EGFR_Adapter_REV	GTG CTC TTC CGA TCT CAG AAA GCT CTA CCG CCT GT
B9_EGFR_Adapter_FOR	GCG TTA TCG AGG TCC TTC TTC TCC GAG ACC CTC A
B9_EGFR_Adapter_REV	GTG CTC TTC CGA TCT GGG GTA CCC TGC CAG AAT AA
B10_EGFR_Adapter_FOR	GCG TTA TCG AGG TCC ACA CTG GAT TGT GAA GAC TTA GG
B10_EGFR_Adapter_REV	GTG CTC TTC CGA TCT AAC CTC CCC ACA GAT ACT ACC C
A15_NF1_Adapter_FOR	GCG TTA TCG AGG TCG AGC TTT GGC TTA GGT TTG AA
A15_NF1_Adapter_REV	GTG CTC TTC CGA TCT GAA ACC CAA ACA TGG CTA TCA

A16_NF1_Adapter_FOR	GCG TTA TCG AGG TCC TTG GGT CTG GGA AAA TGT G
A16_NF1_Adapter_REV	GTG CTC TTC CGA TCT GCA CCT GTG GGT AGT TTG TG
A17_NF1_Adapter_FOR	GCG TTA TCG AGG TCG CTA AAA ACC TCC CAT CTA TGC
A17_NF1_Adapter_REV	GTG CTC TTC CGA TCT CAT CTC TAC TTT CCT GGC TGC T
A18_NF1_Adapter_FOR	GCG TTA TCG AGG TCC GTG TGC CAT ATG GAA AGA TAA
A18_NF1_Adapter_REV	GTG CTC TTC CGA TCT TTC CCT ATG TAG CAA CAG TGG TT
A19_NF1_Adapter_FOR	GCG TTA TCG AGG TCT GAA GGC ATG TGG TTA GAA AGA
A19_NF1_Adapter_REV	GTG CTC TTC CGA TCT CCA CAC TGA AGT TAT GGC AGT C
A20_NF1_Adapter_FOR	GCG TTA TCG AGG TCG AGG GAC AAC CCA CTG AAC A
A20_NF1_Adapter_REV	GTG CTC TTC CGA TCT GTA CTG CGC ATT GTG GGA TA
A21_NF1_Adapter_FOR	GCG TTA TCG AGG TCC CAT TTA AAC GCA TTA AAA ATC AC
A21_NF1_Adapter_REV	GTG CTC TTC CGA TCT TAG CCA TAT GAC GCT TTC AGT T
B11_NF1_Adapter_FOR	GCG TTA TCG AGG TCA AGG ACG GTT GCC AGG AG
B11_NF1_Adapter_REV	GTG CTC TTC CGA TCT GCG GTA AAA TGC ACG TAA CA
B12_NF1_Adapter_FOR	GCG TTA TCG AGG TCC CTG AAA ACC CAT TTT AGT CCT
B12_NF1_Adapter_REV	GTG CTC TTC CGA TCT CCT GTG TTT TCC AGC TAA TTC C
B13_NF1_Adapter_FOR	GCG TTA TCG AGG TCC AGT AAC CCT CTG AGC CCT TTA
B13_NF1_Adapter_REV	GTG CTC TTC CGA TCT GCT TAA AGC TTC CAG ATG AAC C
B14_NF1_Adapter_FOR	GCG TTA TCG AGG TCT CTG CTC TGT GCA AAT GCT T
B14_NF1_Adapter_REV	GTG CTC TTC CGA TCT TGC TAA ATT AAG GCA AAT GAT GA
A22_KDM6A_Adapter_FOR	GCG TTA TCG AGG TCC CCA GTT AAT CGT CAC CCA TA
A22_KDM6A_Adapter_REV	GTG CTC TTC CGA TCT GGC CTG GTT GGT CTC AAA
A23_KDM6A_Adapter_FOR	GCG TTA TCG AGG TCC TGT GAT TTC TGC TTG CCT TTA
A23_KDM6A_Adapter_REV	GTG CTC TTC CGA TCT TCC TTT CCC TAA ATC ACT TTG C
A24_KDM6A_Adapter_FOR	GCG TTA TCG AGG TCG AAA GAC AAT GGG GGT GTG
A24_KDM6A_Adapter_REV	GTG CTC TTC CGA TCT GCT GTT CAG GAG GCT GAG
A25_KDM6A_Adapter_FOR	GCG TTA TCG AGG TCT GCT GGT GTC CTC TAA TTC AGT

A25_KDM6A_Adapter_REV	GTG CTC TTC CGA TCT AAA CCC TAA CTG TTT GCT GAG G
A26_KDM6A_Adapter_FOR	GCG TTA TCG AGG TCT GTG TCC TAA ATC AAA TAC CCA TA
A26_KDM6A_Adapter_REV	GTG CTC TTC CGA TCT ATT TTT GAG TAG GCA ATA CAG TCA
A27_KDM6A_Adapter_FOR	GCG TTA TCG AGG TCG TTA AAA GCA CCC GGA AAC C
A27_KDM6A_Adapter_REV	GTG CTC TTC CGA TCT CCT GAA ACT ATC TGC CAG GTG
A28_KDM6A_Adapter_FOR	GCG TTA TCG AGG TCA GTT GTG TCG ACC CAG TTG A
A28_KDM6A_Adapter_REV	GTG CTC TTC CGA TCT GGG TCT GTG CTC ATT GTG G
B15_KDM6A_Adapter_FOR	GCG TTA TCG AGG TCT TTT TAT TTT TAA CCT TCA GTA GTT CC
B15_KDM6A_Adapter_REV	GTG CTC TTC CGA TCT CCT GCC CAA CAG AGG AAT
B16_KDM6A_Adapter_FOR	GCG TTA TCG AGG TCC CAC CTA TGA ACT TCC TAT TAT GTG A
B16_KDM6A_Adapter_REV	GTG CTC TTC CGA TCT AAA GAG AAT TTT ATG AAG CCA GAT G
B17_KDM6A_Adapter_FOR	GCG TTA TCG AGG TCT TCC CAG GCT AGT CTT GAA CC
B17_KDM6A_Adapter_REV	GTG CTC TTC CGA TCT TCT CAC CAG CTA GTT TGC TAA CAC
A29_STAG2_Adapter_FOR	GCG TTA TCG AGG TCC GAT CTC TGA TCC ATG TTA CCT
A29_STAG2_Adapter_REV	GTG CTC TTC CGA TCT TGC CCT ATA ACT AAA AAT ACT TCC AA
A30_STAG2_Adapter_FOR	GCG TTA TCG AGG TCA GGT AAC ATG GAT CAG AGA TCG
A30_STAG2_Adapter_REV	GTG CTC TTC CGA TCT CAA ATT AGC CCA GAT CAG CA
A31_STAG2_Adapter_FOR	GCG TTA TCG AGG TCT CCC TTC TTA GAC CTG TTT GGT
A31_STAG2_Adapter_REV	GTG CTC TTC CGA TCT TTT TGG CAA TCT GCT AAG TGA A
A32_STAG2_Adapter_FOR	GCG TTA TCG AGG TCG AAT GTT TGC CTT TCT TGA GTT GT
A32_STAG2_Adapter_REV	GTG CTC TTC CGA TCT CTT GGA ATG GAA GAA ATG AAA TCT
A33_STAG2_Adapter_FOR	GCG TTA TCG AGG TCC CTG GCC CCT AAA TTA AAG AC
A33_STAG2_Adapter_REV	GTG CTC TTC CGA TCT AAC AAG AAA CAC CTG TCA GAT ACC
A34_STAG2_Adapter_FOR	GCG TTA TCG AGG TCT CAG GAA CGT AGG AGC CTT AAC T
A34_STAG2_Adapter_REV	GTG CTC TTC CGA TCT TTG AAA AGG GAA AAA CAT TAA AA
A35_STAG2_Adapter_FOR	GCG TTA TCG AGG TCA GCA GCC ACA CCC TGT ATT T
A35_STAG2_Adapter_REV	GTG CTC TTC CGA TCT ACA GCC CGG AGT AGG AAA AA

B18_STAG2_Adapter_FOR	GCG TTA TCG AGG TCT AAA TCG GTC GGC TTC CAT A
B18_STAG2_Adapter_REV	GTG CTC TTC CGA TCT GGT GAT GAG CTA TAG TCA GGA GAA
B19_STAG2_Adapter_FOR	GCG TTA TCG AGG TCG AAG AAC ACC TTT TTG CAA ATC T
B19_STAG2_Adapter_REV	GTG CTC TTC CGA TCT TTG ATG CCA GTT CCT TTT GA
B20_STAG2_Adapter_FOR	GCG TTA TCG AGG TCT GAG CAT TGG TTT TGT TTG C
B20_STAG2_Adapter_REV	GTG CTC TTC CGA TCT AAA GAA CCA GAG CGC TGA ATT A
CDS_APC_17nt_Adapter_FOR	GCG TTA TCG AGG TCT TGA TGA AAG TTG ACT GGC GTA
CDS_APC_17nt_Adapter_REV	GTG CTC TTC CGA TCT CCT CAG GTT CTG GAA AAA TGT C
CDS_APC_23nt_Adapter_FOR	GCG TTA TCG AGG TCT GTT TGC TTG AGC TGC TAG AAC
CDS_APC_23nt_Adapter_REV	GTG CTC TTC CGA TCT AGG CAA AAC AAA ATG TGG GTA A
CDS_ATM_17nt_Adapter_FOR	GCG TTA TCG AGG TCG CCA ATA TTT AAC CAA TTT TGA CC
CDS_ATM_17nt_Adapter_REV	GTG CTC TTC CGA TCT CCT GCT TGA CCT TCA ATG CT
CDS_ATM_23nt_Adapter_FOR	GCG TTA TCG AGG TCG ATG AGG TGA AGT CCA TTG CT
CDS_ATM_23nt_Adapter_REV	GTG CTC TTC CGA TCT GCC ATA CCT GTT TTC CCA AT
CDS_KDM5C_18nt_Adapter_FOR	GCG TTA TCG AGG TCC CAC TTG GGA GGA TTC TTC A
CDS_KDM5C_18nt_Adapter_REV	GTG CTC TTC CGA TCT CAG CCC TCC ATC ACC TAC AT
CACNA1D_Adapter_FOR	GCG TTA TCG AGG TCG GTC CGA CTC AGG AGA TGA A
CACNA1D_Adapter_REV	GTG CTC TTC CGA TCT TTT TTA GGG GAA GCC CAA GT
PPP1R12C_Adapter_FOR	GCG TTA TCG AGG TCG GAC TCC TGC TTC ACA TCG T
PPP1R12C_Adapter_REV	GTG CTC TTC CGA TCT CAC AAT GCA CCT GGG TAA TG
C1_WDR5_Adapter_FOR	GCG TTA TCG AGG TCT CTC TGC AAA GTG GGT GTT G
C1_WDR5_Adapter_REV	GTG CTC TTC CGA TCT CAT CGG AAT CCC AGA AGC TA
C2_COPA_Adapter_FOR	GCG TTA TCG AGG TCT GTG ACT AGG CCA CAC CAA C
C2_COPA_Adapter_REV	GTG CTC TTC CGA TCT CTG CAT GGG TGA ACA AGA AC
C3_STAG2_Adapter_FOR	GCG TTA TCG AGG TCT TTC CCT GTA TAC CAA TCA AGA CA
C3_STAG2_Adapter_REV	GTG CTC TTC CGA TCT TGG CAC GTA AAA GGT GCT AAG TA
C4_STAG2_Adapter_FOR	GCG TTA TCG AGG TCT GGT GAT CCT AAC TGC CCA TA

C4_STAG2_Adapter_REV	GTG CTC TTC CGA TCT CCA TAG CAA GCT GAG ACT GAA G
C5_HDAC2_Adapter_FOR	GCG TTA TCG AGG TCT GGC CAG CAA CAA GTG AAT A
C5_HDAC2_Adapter_REV	GTG CTC TTC CGA TCT TTT GGA TGA GGT CAG CAC AC
C6_HDAC2_Adapter_FOR	GCG TTA TCG AGG TCA ACC TCT AAA ATC ATA CAC AAA CAC TC
C6_HDAC2_Adapter_REV	GTG CTC TTC CGA TCT TGA AGT AGC TTC CTG ATA AAA AGT G
C7_GLUL_Adapter_FOR	GCG TTA TCG AGG TCG CTG GTC AGC AAC ATC ACT C
C7_GLUL_Adapter_REV	GTG CTC TTC CGA TCT CTG TGT AAC TGC CCA AAG CA
C8_GLUL_Adapter_FOR	GCG TTA TCG AGG TCC CTT ACA GCC TCT TGG ATG G
C8_GLUL_Adapter_REV	GTG CTC TTC CGA TCT GGG ATT GCA CTG TGA CCT GT
C9_PARK7_Adapter_FOR	GCG TTA TCG AGG TCA ACT CCT GGG CTC AAA TGA C
C9_PARK7_Adapter_REV	GTG CTC TTC CGA TCT CCA GAG ACC CTG CTC TTC AG
D1_HNF4A_Adapter_FOR	GCG TTA TCG AGG TCT ATT GGA TGG GCT GGT TGA T
D1_HNF4A_Adapter_REV	GTG CTC TTC CGA TCT TCA CAC ATC TGT CCG TTG CT
D2_HNF4A_Adapter_FOR	GCG TTA TCG AGG TCG GTC CTG ATC AGC TTC AAG G
D2_HNF4A_Adapter_REV	GTG CTC TTC CGA TCT CAG TGC CTG GGA GTA AGG AA
D3_ADARB2_Adapter_FOR	GCG TTA TCG AGG TCT GCG CTA CGT GTG TCT GG
D3_ADARB2_Adapter_REV	GTG CTC TTC CGA TCT GCA TTG GCG TCC TCC TG
D4_ASCL2_Adapter_FOR	GCG TTA TCG AGG TCC CTG GGG CGT AAT AAA GAT G
D4_ASCL2_Adapter_REV	GTG CTC TTC CGA TCT AGT TCA CGC TCC CTT GAA GA
D5_KCNA1_Adapter_FOR	GCG TTA TCG AGG TCT CTG GTT CTC CTT CGA GCT G
D5_KCNA1_Adapter_REV	GTG CTC TTC CGA TCT GGA TCT GGA GGC CCT TAG AG
D6_KCNA1_Adapter_FOR	GCG TTA TCG AGG TCC CGC TTG GTA AGG GTT TTT AG
D6_KCNA1_Adapter_REV	GTG CTC TTC CGA TCT TCG GGG ATA CTG GAG AAG TG
D7_WDR5_Adapter_FOR	GCG TTA TCG AGG TCC GCC AGT AGC TGT TCC TAG TG
D7_WDR5_Adapter_REV	GTG CTC TTC CGA TCT CAT TCA CAA AAC CCC ACC TT
D8_WDR5_Adapter_FOR	GCG TTA TCG AGG TCT TAG TTG CGA AGG AGC CAA G
D8_WDR5_Adapter_REV	GTG CTC TTC CGA TCT CAC ACT GCC AGG AGC ACA G

D9_HDAC2_Adapter_FOR	GCG TTA TCG AGG TCT CAT CAT GGT GAT GGT GTT GA
D9_HDAC2_Adapter_REV	GTG CTC TTC CGA TCT TTC AAT TCT GCA TAC AAG TTT CA
D10_HDAC2_Adapter_FOR	GCG TTA TCG AGG TCT GGC ATC CAG GTT CCA TTA T
D10_HDAC2_Adapter_REV	GTG CTC TTC CGA TCT TAC CCC CAT TTG TCT GCT TC
D11_D12_GLUL_Adapter_FOR	GCG TTA TCG AGG TCG GCC AAG CTT CTC GTT TCT A
D11_D12_GLUL_Adapter_REV	GTG CTC TTC CGA TCT CCC CAG CAG AAG GTA CTC AC
D13_SRSF1_Adapter_FOR	GCG TTA TCG AGG TCG TCG CGA CGG CTA TGA TTA C
D13_SRSF1_Adapter_REV	GTG CTC TTC CGA TCT TCC CTT CAC ATC AAT CCA CA
D14_SRSF1_Adapter_FOR	GCG TTA TCG AGG TCC CTA CAT CCG GGT TAA AGT TGA
D14_SRSF1_Adapter_REV	GTG CTC TTC CGA TCT GGG TTC TAC AAA AAG TGT CAC CA
D15_SOD1_Adapter_FOR	GCG TTA TCG AGG TCG CGG AGG TCT GGC CTA TAA
D15_SOD1_Adapter_REV	GTG CTC TTC CGA TCT TGC CTT CTG CTC GAA ATT G
D16_SOD1_Adapter_FOR	GCG TTA TCG AGG TCC AGA TGA CTT GGG CAA AGG T
D16_SOD1_Adapter_REV	GTG CTC TTC CGA TCT GCA ACT CTG AAA AAG TCA CAC AA
D17_VIM_Adapter_FOR	GCG TTA TCG AGG TCG GAG CTA CGT GAC TAC GTC CA
D17_VIM_Adapter_REV	GTG CTC TTC CGA TCT GCT CCA CCT TCT CGT TGG T
D18_VIM_Adapter_FOR	GCG TTA TCG AGG TCC ATC CTC CAT GTC CTG TCT TT
D18_VIM_Adapter_REV	GTG CTC TTC CGA TCT AGA ACA GAC AGA CGG TCA GC

Appendix S1 Sequences of donor ssODNs.

CACNA1D

47nt homology arms (NT, symmetric):
CTATTTCAGGGACCCCCACTGCTTGGGGGAGCAGGAGTATTTCAGTA**tctaga**GAGGATGAC
AGCTCGCCCACCTGGAGCAGGTGAGCTGCTCTGGCTCC

42nt homology arms (NT, symmetric):
TCAGGGACCCCCACTGCTTGGGGGAGCAGGAGTATTTCAGTA**tctaga**GAGGATGACAGCTC
GCCCACCTGGAGCAGGTGAGCTGCTCTG

37nt homology arms (NT, symmetric):
GACCCCCACTGCTTGGGGGAGCAGGAGTATTTCAGTA**tctaga**GAGGATGACAGCTCGCCCA
CCTGGAGCAGGTGAGCTG

37nt homology arms (T, symmetric):
CAGCTCACCTGCTCCAGGTGGGCGAGCTGTCATCCTC**tctaga**TACTGAAATACTCCTGCTC
CCCCAAGCAGTGGGGGTC

32nt homology arms (NT, symmetric):
CCACTGCTTGGGGGAGCAGGAGTATTTCAGTA**tctaga**GAGGATGACAGCTCGCCCACCTGG
AGCAGGTG

27nt homology arms (NT, symmetric):
GCTTGGGGGAGCAGGAGTATTTCAGTA**tctaga**GAGGATGACAGCTCGCCCACCTGGAGC

27nt homology arms (T, symmetric):
GCTCCAGGTGGGCGAGCTGTCATCCTC**tctaga**TACTGAAATACTCCTGCTCCCCCAAGC

37/77nt homology arms (NT, asymmetric):
GACCCCCACTGCTTGGGGGAGCAGGAGTATTTCAGTA**tctaga**GAGGATGACAGCTCGCCCA
CCTGGAGCAGGTGAGCTGCTCTGGCTCCTGTGGAGAGCGGGAGGCCGCCCTGCCCTGG

37/77nt homology arms (T, asymmetric):
CCAGGGCAGGGCGGCCTCCCGCTCTCCACAGGAGCCAGAGCAGCTCACCTGCTCCAGGTGGG
CGAGCTGTCATCCTC**tctaga**TACTGAAATACTCCTGCTCCCCCAAGCAGTGGGGGTC

77/37nt homology arms (NT, asymmetric):
TATTTGCCGGGAAGACCCAGAGATACATGGCTATTTCAGGGACCCCCACTGCTTGGGGGAGC
AGGAGTATTTCAGTA**tctaga**GAGGATGACAGCTCGCCCACCTGGAGCAGGTGAGCTG

77/37nt homology arms (T, asymmetric):
CAGCTCACCTGCTCCAGGTGGGCGAGCTGTCATCCTC**tctaga**TACTGAAATACTCCTGCTC
CCCCAAGCAGTGGGGGTCCCTGAAATAGCCATGTATCTCTGGGTCTTCCCGGCAAATA

PPP1R12C

47nt homology arms (NT, symmetric):
CGGGAGTCCGCTGGGGGCGCCGTGGAGGCTGTGGGGACGTTTGGCTT**tctaga**GCTCCGGAA
TCCTGGAGGGAGGCGAGGAGTTCTCCAAGCAAGGAGGA

42nt homology arms (NT, symmetric):
GTCCGCTGGGGGCGCCGTGGAGGCTGTGGGGACGTTTGGCTT**tctaga**GCTCCGGAATCCTG
GAGGGAGGCGAGGAGTTCTCCAAGCAAG

37nt homology arms (NT, symmetric):
CTGGGGGCGCCGTGGAGGCTGTGGGGACGTTTGGCTT**tctaga**GCTCCGGAATCCTGGAGGG
AGGCGAGGAGTTCTCCAA

37nt homology arms (T, symmetric):
TTGGAGAACTCCTCGCCTCCCTCCAGGATTCCGGAGC**tctaga**AAGCCAAACGTCCCCACAG
CCTCCACGGCGCCCCCAG

32nt homology arms (NT, symmetric):
GGCGCCGTGGAGGCTGTGGGGACGTTTGGCTT**tctaga**GCTCCGGAATCCTGGAGGGAGGCG
AGGAGTTC

27nt homology arms (NT, symmetric):
CGTGGAGGCTGTGGGGACGTTTGGCTT**tctaga**GCTCCGGAATCCTGGAGGGAGGCGAGG

27nt homology arms (T, symmetric):
CCTCGCCTCCCTCCAGGATTCCGGAGC**tctaga**AAGCCAAACGTCCCCACAGCCTCCACG

37/77nt homology arms (NT, asymmetric):
CTGGGGGCGCCGTGGAGGCTGTGGGGACGTTTGGCTT**tctaga**GCTCCGGAATCCTGGAGGG
AGGCGAGGAGTTCTCCAAGCAAGGAGGAGGCTTGGTGACCTCAGACCTGCATCAATTC

37/77nt homology arms (T, asymmetric):
GAATTGATGCAGGTCTGAGGTCACCAAGCCTCCTCCTTGCTTGGAGAACTCCTCGCCTCCCT
CCAGGATTCCGGAGC**tctaga**AAGCCAAACGTCCCCACAGCCTCCACGGCGCCCCCAG

77/37nt homology arms (NT, asymmetric):
CTGCCCTGGCCTCTTCACCTCCGTCGGTCCCGGGAGTCCGCTGGGGGCGCCGTGGAGGCTGT
GGGGACGTTTGGCTT**tctaga**GCTCCGGAATCCTGGAGGGAGGCGAGGAGTTCTCCAA

77/37nt homology arms (T, asymmetric):
TTGGAGAACTCCTCGCCTCCCTCCAGGATTCCGGAGC**tctaga**AAGCCAAACGTCCCCACAG
CCTCCACGGCGCCCCCAGCGGACTCCCGGGACCGACGGAGGTGAAGAGGCCAGGGCAG

A12 (EGFR)

47nt homology arms (NT, symmetric):
AAAAGGCCTTTCTGGGAAGCAACGGTAGCCACATATTTAGTATCAGA**aagctt**CAAGGGAGT
TTATGGGAGGCAATGCATATTTCACGCACTCTTATTCT

B4 (ALK)

47nt homology arms (NT, symmetric):
CATTTCTCCAATTCCAGATAGTAAAGAAGCCTTTGGTCTCTTGCTGG**tctaga**TGTGGATCC
AGCTGCTTCTTGAATGAACTTTCATCAAGGCTTCTTTG

A3 (ALK)

27nt homology arms (NT, symmetric):
TGGGCGTCACATTTAGTGGACAAACAC**aagctt**GTAACATACACACTCAGGAGTAATACC

27nt homology arms (T, symmetric):
GGTATTACTCCTGAGTGTGTATGTTAC**aagctt**GTGTTTGTCCACTAAATGTGACGCCCA

A11 (EGFR)

27nt homology arms (NT, symmetric):
ATTCCTTATTATTTAGTGTTAGTTTGA**aagctt**GGTGTTTGGTTTCACTGAATGTTTGTG

27nt homology arms (T, symmetric):
CACAAACATTCAGTGAAACCAAACACC**aagctt**TCAAACTAACACTAAATAATAAGGAAT

B8 (EGFR)

27nt homology arms (NT, symmetric):
GAAAGAGGCGGTTTGGTAATGAAATGA**aagctt**AGATTACGGTCATTTCAAGTTATATTC

27nt homology arms (T, symmetric):
GAATATAACTTGAAATGACCGTAATCT**aagctt**TCATTTCATTACCAAACCGCCTCTTTC

25nt homology arms (NT, symmetric):
AAGAGGCGGTTTGGTAATGAAATGA**aagctt**AGATTACGGTCATTTCAAGTTATAT

23nt homology arms (NT, symmetric):
GAGGCGGTTTGGTAATGAAATGA**aagctt**AGATTACGGTCATTTCAAGTTAT

21nt homology arms (NT, symmetric):
GGCGGTTTGGTAATGAAATGA**aagctt**AGATTACGGTCATTTCAAGTT

19nt homology arms (NT, symmetric):
CGGTTTGGTAATGAAATGA**aagctt**AGATTACGGTCATTTCAAG

17nt homology arms (NT, symmetric):
GTTTGGTAATGAAATGA**aagctt**AGATTACGGTCATTTCA

17nt homology arms (T, symmetric):
TGAAATGACCGTAATCT**aagctt**TCATTTCATTACCAAAC

B18 (STAG2)

27nt homology arms (NT, symmetric):
AGGGACAGTTGTTTAGGACTATAGATT**aagctt**GATTGTAGAGACTTTAGAGATTTCGTT

27nt homology arms (T, symmetric):
AACGAAATCTCTAAAGTCTCTACAATC**aagctt**AATCTATAGTCCTAAACAACTGTCCCT
